# Spin Crossover Quenching by “Racemization” in a Family of *trans*-1,2-Di(tetrazol-1-yl)cyclopentane-Based Fe(II) 1D Coordination Polymers

**DOI:** 10.1021/acs.inorgchem.4c02671

**Published:** 2024-09-12

**Authors:** Vladyslav Maliuzhenko, Marek Weselski, Janusz Gregoliński, Maria Książek, Joachim Kusz, Robert Bronisz

**Affiliations:** † Faculty of Chemistry, University of Wrocław, F. Joliot-Curie 14, 50-383 Wrocław, Poland; ‡ Institute of Physics, University of Silesia, 75 Pułku Piechoty 1, 41-500 Chorzów, Poland

## Abstract

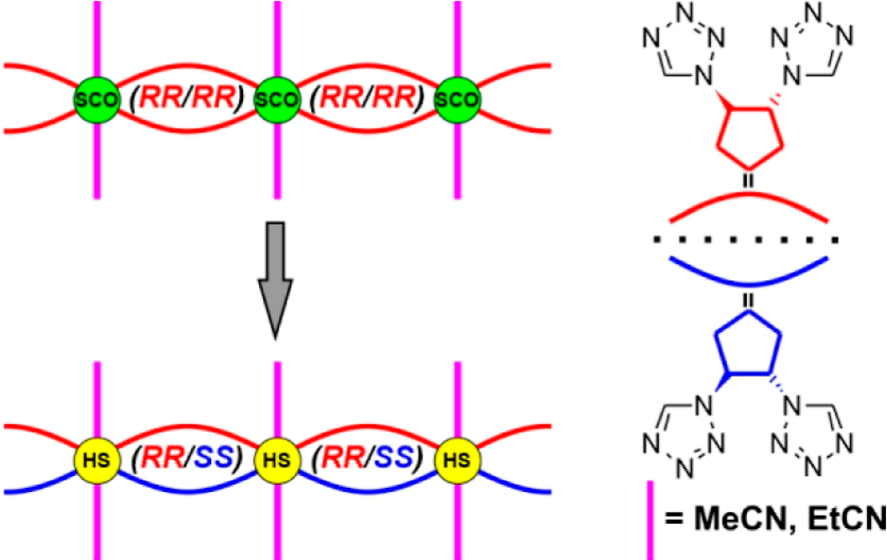

Optically pure (*RR*)- and racemic (*RR*/*SS*)-*trans*-1,2-di(tetrazol-1-yl)cyclopentane were synthesized and used to prepare homo- and heterochiral Fe(II) coordination compounds. [Fe((*RR*/*SS*)-C_7_H_10_N_8_)_2_(CH_3_CN)_2_](BF_4_)_2_ (**1A**), [Fe((*RR*/*SS*)-C_7_H_10_N_8_)_2_(C_2_H_5_CN)_2_](BF_4_)_2_ (**2A**), [Fe((*RR*)-C_7_H_10_N_8_)_2_(CH_3_CN)_2_](BF_4_)_2_·2CH_3_CN (**1B·solv**), and [Fe((*RR*)-C_7_H_10_N_8_)_2_(C_2_H_5_CN)_2_](BF_4_)_2_ (**2B**) form a family of one-dimensional coordination polymers. Fe(II) cations in these complexes are characterized by a heteroleptic coordination environment: the neighboring metal centers are bridged by two 1,2-di(tetrazol-1-yl)cyclopentane molecules, while the nitrile molecules (acetonitrile or propionitrile, respectively) occupy the axial positions. Independently of the kind of nitrile coligands, an ability to thermally induce spin crossover (SCO) is governed by chirality. **1B·solv** and **2B** exhibit abrupt and complete SCO occurring at *T*_1/2_ = 144 K and *T*_1/2_ = 228 K, respectively. Desolvated form, **1B** (of the same stoichiometry as **2B**), also exhibits SCO (*T*_1/2_ = 215 K). In contrast, an exchange within the polymeric chain of half of the *RR* molecules with the *SS* enantiomeric form results in formation of **1A** and **2A**, which remain in stable high-spin (HS) form down to 10 K. It has been shown that moving from a homochiral to a heterochiral system changes the structure of the polymeric unit (while maintaining the same polymer dimensionality and bridging fashion) that leads to the deep reorganization of the further coordination spheres, including the anion network.

## Abstract 2

An application of chiral and racemic *trans*-1,2-di(tetrazol-1-yl)cyclopentane ligands leads to formation of a family of heteroleptic 1D coordination polymers. Independently of the kind of axially coordinated nitrile coligands (acetonitrile and propionitrile), an ability to thermally induce spin crossover is governed by the chirality of bridging bis(azolyl)-type molecules. Coordination polymers based on heterochiral ligands exhibit spin crossover, whereas racemic analogues remain in HS form down to 10 K.

## Introduction

Among the physicochemical functions, an ability to switch the spin state of a metal ion by application of an external stimulus is particularly interesting from the point of view of potential applications. The most spectacular changes develop in Fe(II) (3d^6^) compounds due to the transition from high-spin (t_2g_^4^e_g_^2^, HS) to low-spin (t_2g_^6^, LS) state accompanied by the change of magnetic, optical, dielectric, and structural properties.^1^ The spin state can be switched by changing the temperature, applied pressure, light irradiation, or by the action of a chemical agent. Hence, a variety of switching possibilities in combination with read-out methods make spin crossover (SCO) systems interesting for potential applications.^2^ These compounds are considered as materials in medicinal diagnostic,^3^ in displays,^4^ memory devices,^5^ and as sensors.^6^ SCO-based materials can represent magneto-optical device applicability due to the significant redistribution of electronic states. It is expected that, in comparison to achiral photochromic SCO complexes, a combination of chirality and SCO properties can create fundament for the nondestructive reading of stored information.^7^ For this reason, chiral SCO materials are becoming of great importance.

The most commonly used strategy depends on the preparation of chiral complex molecules. It can be accomplished by exploiting spontaneous resolution phenomena or chiral ligand molecules. The most spectacular example representing the first approach is 3D network (+)-Fe_2_[Nb(CN)_8_](4-bromopyridine)_8_·2H_2_O in which light-induced switching between vertical and horizontal polarization planes can be realized for.^7^ Spontaneous resolution was also achieved by an application of an achiral tris{[2-{(imidazole-4-yl)methylidene}amino]ethyl}amine which resulted in a mixed-valence Fe(II)/Fe(III) system.^8^ The chiral metal–organic framework exhibiting SCO properties, [Fe^II^(mptpy)_2_]EtOH·0.2DMF [mptpy = 3-methyl-2-(5-(4-(pyridin-4-yl)phenyl)-4*H*-1,2,4-triazol-3-yl)-pyridine], has been solvothermally synthesized through spontaneous resolution, too.^9^ This complex undergoes a two-step spin transition; however, spontaneous resolution being an origin of the chiral product did not give the possibility to perform studies on the racemic form. In this case, the preparation of the corresponding species, based on racemic ligands, was unsuccessful because of the formation of chemically different species. The presented examples indicate that obtaining material based on spontaneous separation is a matter of chance. The complexes [Fe(bqen)(NCX)_2_] [bqen = *N*,*N*′-bis(8-quinolyl)ethane-1,2-diamine, X = S and Se] characterized by different SCO temperatures are important examples because they play a role of a bridge between uncontrolled formation of chiral species by spontaneous chiral resolution and controlled preparation of complexes by exploitation of chiral ligands.^10^ In this case, it has been shown that the controllable synthesis of achiral (orthorhombic system) or chiral (trigonal system) compounds is feasible and depends on the synthesis method.

A second strategy depends on the use of optically active ligand molecules. Chiral *R*-*N*2,*N*2′-bis(pyridin-2-ylmethyl)-1,1′-binaphthyl-2,2′-diamine (*R*-pab) forms magnetically and electrically bistable 1D coordination polymer [Co^II^((*R*)-pabn)][Fe^III^(tp)(CN)_3_](BF_4_)·MeOH·2H_2_O exhibiting the phenomenon of electron transfer coupled with spin transition.^11^ A strategy of exploiting optically pure ligands was applied also in the synthesis of [Fe(L)(bpz)_2_] [bpz = bis(1-pyrazolyl)borohydride, L = (*R*)/(*S*)-4,5-pinenepyridyl-2-pyrazine].^12^ In this case, chirality, SCO, and dielectric switching were combined in a single homochiral compound; however, studies on the coupling between chirality and SCO were not carried out. Some differences between SCO properties for homochiral and racemic species were found for mononuclear complexes, [Fe(L^sub^)_2_][ClO_4_]_2_ [L^sub^ = 2,6-bis(oxazolinyl)pyridine-type chiral ligands]^13^ and [Fe(L)_2_(NCS)_2_] [L = α-methyl-*N*-(2-pyridinylmethylene)cyclohexanemethanamine],^14^ and polynuclear systems, Fe_2_[Nb(CN)_8_](L)_8_·6H_2_O [L = *R*-, *S*-, or *rac*-1-(3-pyridyl)ethanol].^15^ Stronger differentiation of SCO properties, resulting from an application of chiral and racemic Schiff-based ligands, was observed in a mononuclear 7-coordination compound–chiral complex exhibiting SCO, whereas the racemic compound showed very gradual and incomplete SCO.^16^ The successful differentiation of SCO properties was achieved between optically pure and racemic mononuclear species [Fe(L)_3_](ClO_4_)_2_ [L = *R* or *S*-1-phenyl-*N*-(1-alkyl-1*H*-imidazole-2-ylmethylene)ethanamine]. In this case, the optically pure system remains in HS form down to 10 K, whereas the racemic system exhibits thermally induced SCO.^17^ In contrast, optically active system [Fe(L)(NCSe)_2_] [L = *N*1,*N*2-bis((pyridin-2-yl)methyl)cyclohexane-1,2-diamine] exhibits thermally induced SCO, whereas its racemic form remains in HS form down to 10 K.^18^

It should be noted that an incorporation of a chirality into the SCO system can be made in other ways including the usage of chiral anions as well as electrically neutral chiral guest molecules. This approach was applied for 4-amino-1,2,4-triazole-based systems containing chiral camphorosulfonate anions.^19^ Another example is an incorporation of chiral *S-* or *R*-2-butanol molecules into a crystal lattice.^20^ It leads to the formation of diastereomeric species, resulting in chiral recognition monitored by a shift of the SCO temperature. On the contrary, an application of a mononuclear system based on homochiral chelating Schiff-type ligands allowed separation of chiral nitriles; however, formation of diastereomeric products did not involve a change of SCO properties.^21^

It is visible that the introduction of chirality allows drastic differences in SCO properties. Concomitantly systematic research on the influence of chirality on the SCO properties is sparse. The reason for this is the fact that chemical modification of such ligands may lead to a loss of repeatability of the structural motifs in a series of homologous compounds. Studies on the coordination properties of di(tetrazol-2-yl)alkanes have shown that they can form heteroleptic systems in which the equatorial plane is formed by four tetrazole rings, and the axial positions are occupied by nitrile molecules. What is important in the families of ebtz (1,2-di(tetrazol-2-yl)ethane)^22^ and hbtz (1,6-di(tetrazol-2-yl)hexane)-based^23^ coordination compounds of Fe(II) is that the motif of the polymeric units is preserved regardless of the type of the nitrile coligand. From the point of view of studying the influence of chirality on SCO properties, maintaining the same structural motif is an extremely desirable feature. Recent studies using di(tetrazol-1-yl)methane have shown the possibility of forming systems of the [Fe(tetrazol-1-yl)_4_(RCN)_2_] type, thus allowing to conduct systematic modification using axially attached nitrile molecules.^24^ In this work, we present that the use of *trans*-1,2-di(tetrazol-1-yl)cyclopentane in the form of an *RR* enantiomer or *RR*/*SS* racemic mixture, as a bridging ligand, leads to the formation of the one-dimensional (1D) coordination polymer family. In these compounds, the structural motif of the polymeric chain is preserved, regardless of the type of axially coordinated nitrile molecules (R = –CH_3_ and –C_2_H_5_), and SCO properties are governed by the chirality that is transferred on the anion network and network of weak intermolecular interactions.

## Results and Discussion

### General Characterization

**Scheme 1:**
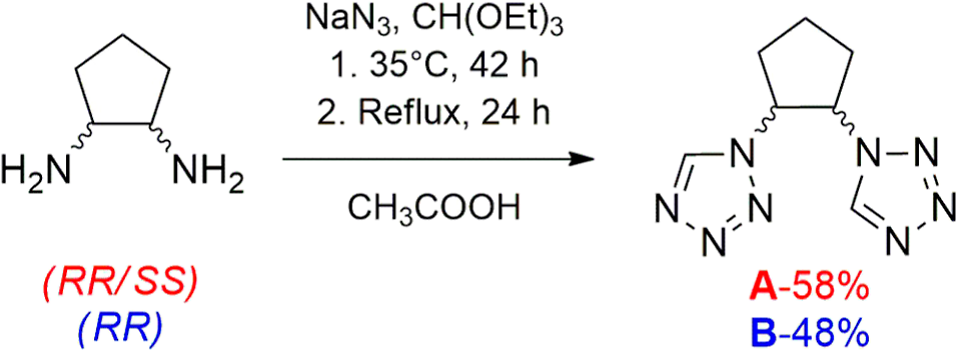
Synthetic Route for the Preparation of the Desired Ligands (**A** = Racemic *RR*/*SS* and **B** = Homochiral *RR*)

(*RR*)-Cyclopentane-1,2-diamine dihydrochloride and racemic (*RR*/*SS*)-cyclopentane-1,2-diamine dihydrochloride were prepared according to the modified literature procedures.^25^ The racemic and enantiomeric pure tetrazol-1-yl-based ligands were synthesized by heterocyclization reaction involving corresponding primary diamine dihydrochloride salt, triethyl orthoformate, and sodium azide (Scheme 1).^26^ The presence of the ν(CH) band on the FT-IR spectrum at 3140 cm^–1^ is characteristic of 1-substituted tetrazoles (Figures S5 and S6). The desired ligands were isolated as colorless crystals (soluble in methanol, acetonitrile, and propionitrile) from the corresponding crude reaction mixtures using flash chromatography (see the Experimental section for details). The structures of the obtained ligands were confirmed by single-crystal X-ray diffraction (SC-XRD) methods (Figure S15, see the Supporting Information for details).

The coordination compounds were prepared under a nitrogen atmosphere in a reaction of iron(II) tetrafluoroborate hexahydrate with the corresponding ligand in stoichiometry 1:2 (**A** denotes the racemic-base and **B** denotes the homochiral-based compounds) using acetonitrile or propionitrile (**1** denotes acetonitrile and **2** denotes propionitrile) as a solvent. Thus, four coordination compounds were obtained: [Fe((*RR*/*SS*)-C_7_H_10_N_8_)_2_(CH_3_CN)_2_](BF_4_)_2_ (**1A**), [Fe((*RR*/*SS*)-C_7_H_10_N_8_)_2_(C_2_H_5_CN)_2_](BF_4_)_2_ (**2A**), [Fe((*RR*)-C_7_H_10_N_8_)_2_(CH_3_CN)_2_](BF_4_)_2_·2CH_3_CN (**1B·solv**), and [Fe((*RR*)-C_7_H_10_N_8_)_2_(C_2_H_5_CN)_2_](BF_4_)_2_ (**2B**). Three of them apart from **2B** were isolated as colorless crystals; **2B** was obtained as pale-pink crystals. Preliminary studies on the occurrence of the thermochromism phenomenon of the macroscopic samples revealed that complexes **1A** and **2A** (based on racemic ligand **A**) did not change their coloring under cooling in liquid nitrogen. Meanwhile, complexes based on homochiral ligand **B** changed their color under cooling: product **1B·solv** crystallized from acetonitrile solution changed its color to dark violet under cooling in liquid nitrogen, while product **2B** obtained from propionitrile solution changed its color to dark pink under slight cooling (≈−20 °C). The appearance of violet color results from ^1^A_1_ → ^1^T_1_ transition, which indicates thermally induced SCO.

### Magnetic and Photomagnetic Studies

**Figure 1:**
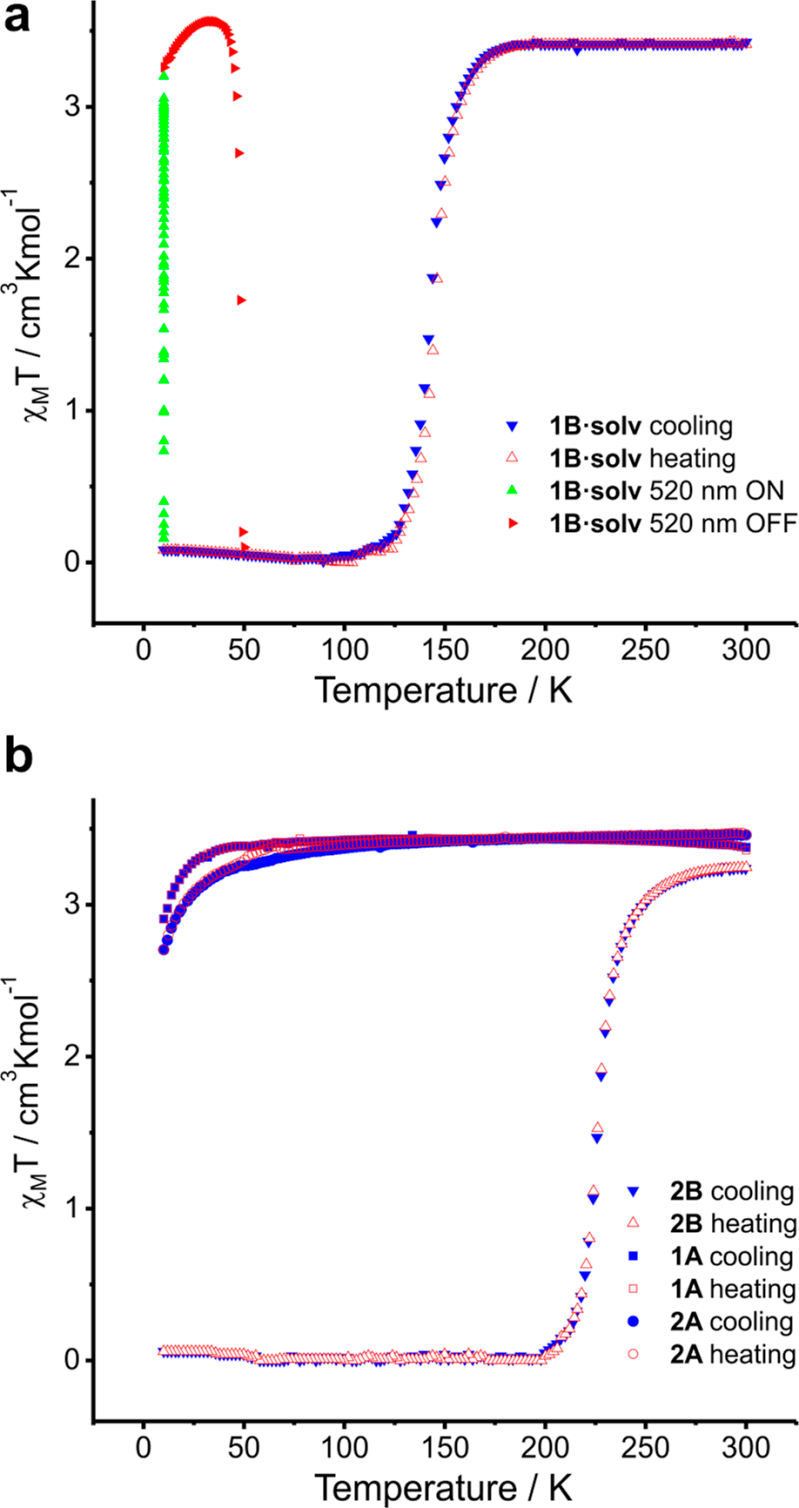
χ_*M*_*T*(*T*) dependence of the studied coordination compounds.

Temperature-dependent measurements of the magnetic susceptibility (Figure 1) confirmed the above mentioned: the value of χ_*M*_*T* for the heterochiral compounds **1A** and **2A** is equal to 3.46 cm^3^ K mol^–1^ at RT [which is a characteristic value for the HS state of the Fe(II) complexes] and did not change under cooling down to 10 K. Meanwhile, homochiral compounds **1B·solv** and **2B** exhibit SCO induced by cooling of the sample. In **2B**, spin transition takes place slightly below RT (*T*_1/2_ = 228 K) and is not accompanied by a hysteresis loop, while in **1B·solv**, SCO transition is more abrupt and shifted toward significantly lower temperatures (*T*_1/2_ = 144 K). Irradiation of LS form of the **1B·solv** sample at 10 K with a green laser (520 nm) leads to the generation of the metastable HS state (LIESST phenomenon).^27^ This photoinduced state of **1B·solv** is stable up to about 40 K, and further heating of the sample leads to its relaxation to the thermodynamically stable (at this temperature range) LS state. An attempt to generate a photoinduced LS phase at 10 K in heterochiral HS **1A** and **2A** by irradiation (reverse LIESST) of corresponding samples with a red laser (808 nm) has failed.

### SC-XRD Studies

**Figure 2:**
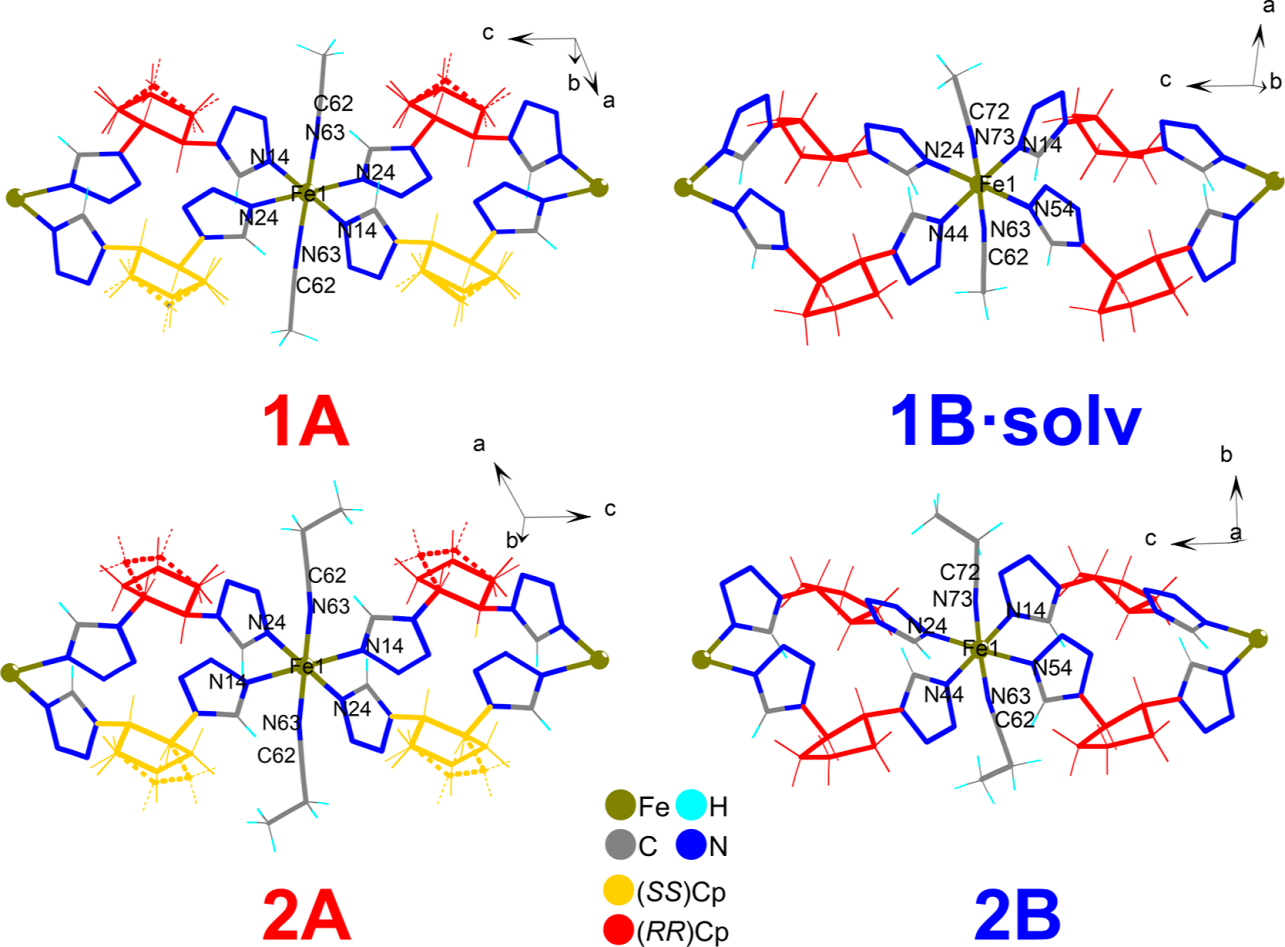
First coordination sphere of Fe(II) cations and bridging mode in homochiral and heterochiral coordination compounds. Noncoordinating solvent molecules and counterions are omitted for clarity. Red and yellow colors denote the *trans-*1,2-disubstituted cyclopentane rings with different absolute configurations (*RR* and *SS*, respectively). The minor disordered component is shown with a dashed line.

The results of the SC-XRD experiments performed on the obtained coordination compounds were surprising due to the apparently close structural similarities of these complexes being in contrast with the results of the magnetic studies. All four compounds belong to the family of 1D coordination polymers with a heteroleptic coordination environment. Iron(II) neighboring cations in these compounds are bridged by two ligand molecules (coordinated through nitrogen atom *N*4 of the corresponding 1-substituted tetrazole rings) forming a polycationic polymeric chain (see Figure 2), while axial coordination positions are occupied by nitrile molecules (acetonitrile in **1A** and **1B** and propionitrile in **2A** and **2B**, respectively) coordinating through a nitrogen atom.

It should be emphasized that 1-substituted tetrazoles usually form a homoleptic coordination environment around the Fe(II) center coordinating in monodentate mode through *exo* nitrogen atom *N*4. The coordination compounds of Fe(II) based on derivatives of 1-monosubstituted tetrazoles with heteroleptic coordination environment are very rare, yet not unknown. For example, Reedijk and Stierstorfer reported crystal structures, in which four 1-substituted tetrazole rings and two water molecules coordinate to the Fe(II) cation.^28^ Another example of a heteroleptic complex is the mononuclear system [Fe(*i*-ptz)_4_(SCN)_2_] [*i*-ptz = 1-isopropyltetrazole].^29^ Aforementioned complexes remain in stable HS form in the whole temperature range. Another heteroleptic complex is the one containing nitrile molecules as coligands. Recently, we reported Fe(II) coordination compounds containing acetonitrile^30^ or various dinitrile (adiponitrile,^24^ glutaronitrile, and suberonitrile^31^) molecules along with 1-substituted tetrazole donors. The last example of heteroleptic compounds was obtained by us in a reaction of Fe(ClO_4_)_2_·6H_2_O with bitopic 1-(tetrazol-1-yl)-3-(1,2,3-triazol-1-yl)propane and was characterized by the presence of 1,2,3-triazole and tetrazole rings in the first coordination sphere of Fe(II).^32^ Interestingly, all of the above-mentioned Fe(II) coordination polymers, reported by us previously, can undergo thermally induced SCO. That fact is in contrast to the heterochiral compounds **1A** and **2A** (which are presented in this work) that remain in the HS state.

**Scheme 2:**
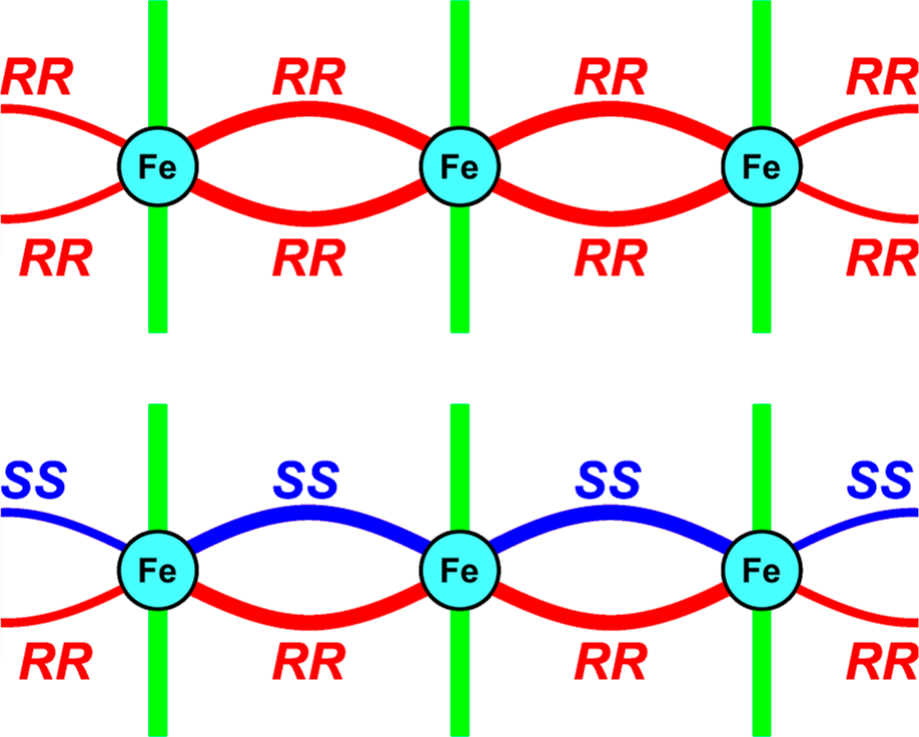
Schematic Presentation of Polymeric Unit Topology in Homo- (**1B·solv** and **2B**) and Heterochiral (**1A** and **2A**) Compounds. Curved Linkers Denote Bridging Ligands, whereas Straight Lines Coordinate Nitriles

According to expectations, the cyclopentane-based ligands act as bridging molecules. Two ligand molecules join two neighboring Fe(II) ions. This bridging mode leads to the formation of polymeric macrocations. The polymeric chains in these complexes are aligned with the [001] crystallographic direction. Polycationic chains in racemic-based **1A** and **2A** are formed by both optical isomers of the used ligand (Figure 2, left) bridging metal centers; thus, polymeric units can be denoted as heterochiral chains (Scheme 2). Apparently, polycationic chains in **1B·solv** and **2B** are homochiral (Figure 2, right).

Both **1A** and **2A** crystallize in the *P*2_1_/*n* space group. Iron(II) cations lie on the inversion center in these compounds (Figure S17). **1B·solv** crystallizes in the *P*1 space group and besides coordinated acetonitrile molecules, it also contains two noncoordinated solvent molecules (Figure S17) per one formal molecule. Interestingly, the other three coordination compounds, isostructural with **1B·solv**, are not solvated. **2B** crystallizes in the Sohncke *P*2_1_2_1_2_1_ space group: higher symmetry, comparing to **1B·solv**, can be explained by the lack of noncoordinated solvent molecules.

**Figure 3:**
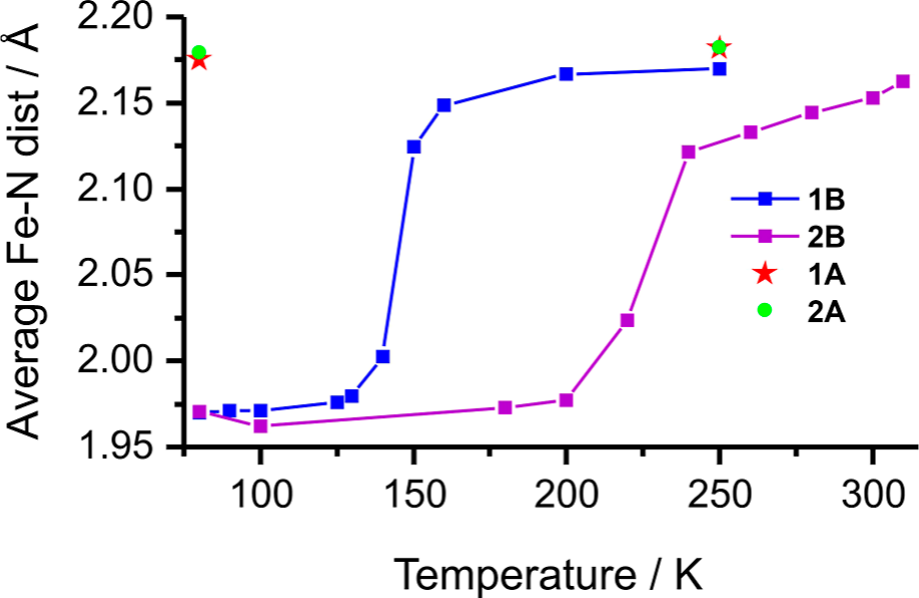
Average Fe–N distances in a function of temperature in crystal structures of coordination compounds.

In contrast to the aforementioned racemic-based compounds and variable-temperature SC-XRD, the average Fe–N distances determined at high temperatures (250 K for **1A**, **2A**, and **1B·solv** and 310 K for **2B**, respectively) are equal to 2.182(2) (**1A**), 2.183(4) (**2A**), 2.170(3) (**1B·solv**), and 2.163(5) (**2B**) Å (Table 1), which are typical bond lengths for the HS state of the [FeN_6_] chromophore. Cooling to 80 K did not induce significant changes in Fe–N distances in heterochiral **1A** and **2A**: the average values of Fe–N bonds in these compounds are equal to 2.175(1) and 2.172(3) Å, which are typical for the HS form of Fe(II) coordination compounds (see Table 1 and Figure 3).

**Table 1:**
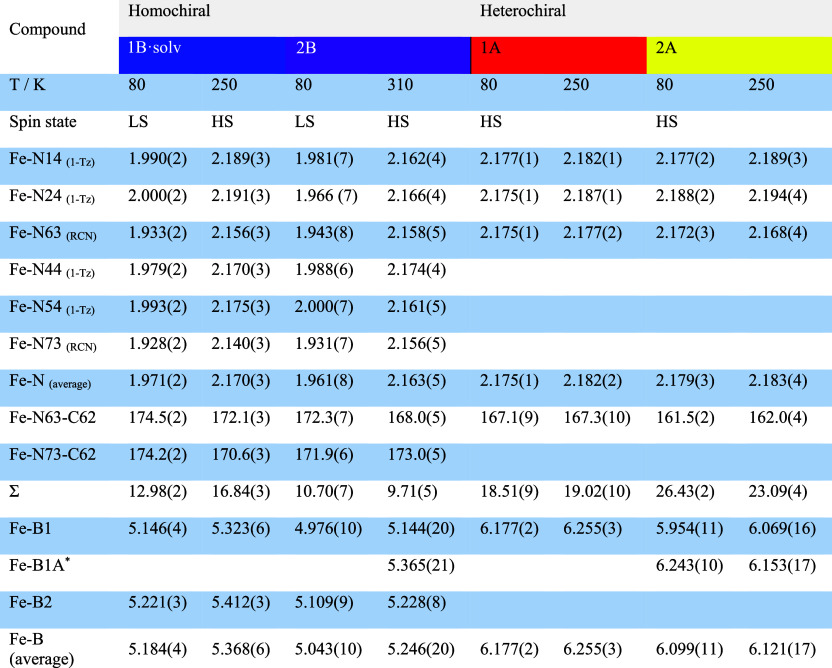
Selected Interatomic Distances and Angles in Crystal Structures Determined at 80 and 250 K (**1A**, **2A**, and **1B·solv**) or at 80 and 310 K (**2B**)^a^

a For homochiral compounds (**1B·solv** and **2B**), selected temperatures correspond to the different spin states. R = CH_3_ (**1A** and **1B·solv**), C_2_H_5_ (**2A and 2B**), 1-Tz = 1-substituted tetrazole ring. Σ is calculated according to , where α = 12 *cis*-N–Fe–N angles about the iron ion. * denotes a minor disordered component.

Measurements of homochiral-based **1B·solv** and **2B** (performed in the 80–250 K and 80–310 K temperature range, respectively) confirm the nature of the SCO curve in these compounds: average Fe–N distance as a function of temperature curves (see Table 1 and Figure 3) is in an excellent agreement with magnetic measurements of polycrystalline samples. The average values of Fe–N distances determined at 80 K are equal to 1.971(2) and 1.961(8) Å for **1B·solv** and **2B**, respectively, which are typical bond lengths for the LS form of Fe(II) coordination compounds (see Table 1 and Figure 3).

Besides the apparent similarity of the first coordination sphere of the Fe(II) cation in both homo- and heterochiral compounds, taking a closer look at the conformation of coordinated tetrazole rings in relation to the equatorial plane [defined by four nitrogen atoms of tetrazole rings and iron(II) cation] reveals the key difference between obtained compounds. Namely, the dihedral angles between the equatorial plane and the plane of the corresponding tetrazole ring in homochiral compound **1B·solv** are close to 87.0, 82.9, 74.8, and 83.0° at 80 K (Table 2). The corresponding angles in propionitrile-based **2B** are smaller (45.7, 67.9, 55.7, and 46.0°, respectively). At the same time, the analogous dihedral angles in heterochiral analogues **1A** and **2A** are close to 20 and 80° at 80 K, respectively.

**Table 2:**
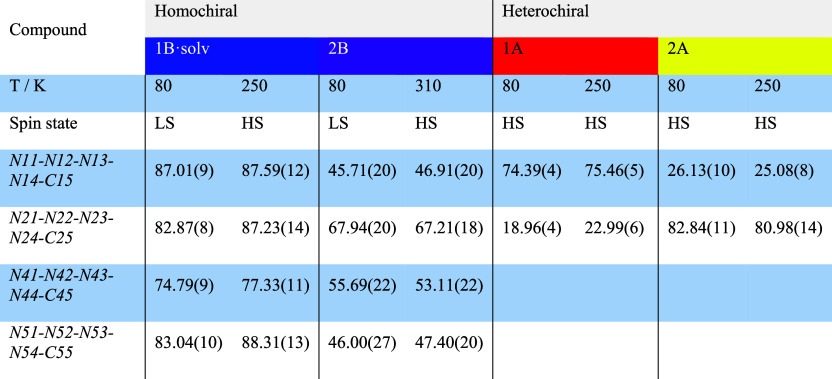
Dihedral Angles between the Equatorial Plane Defined by Tetrazole Nitrogen Atoms Coordinated to Fe(II) and the Plane of Corresponding Tetrazole Rings

**Figure 4:**
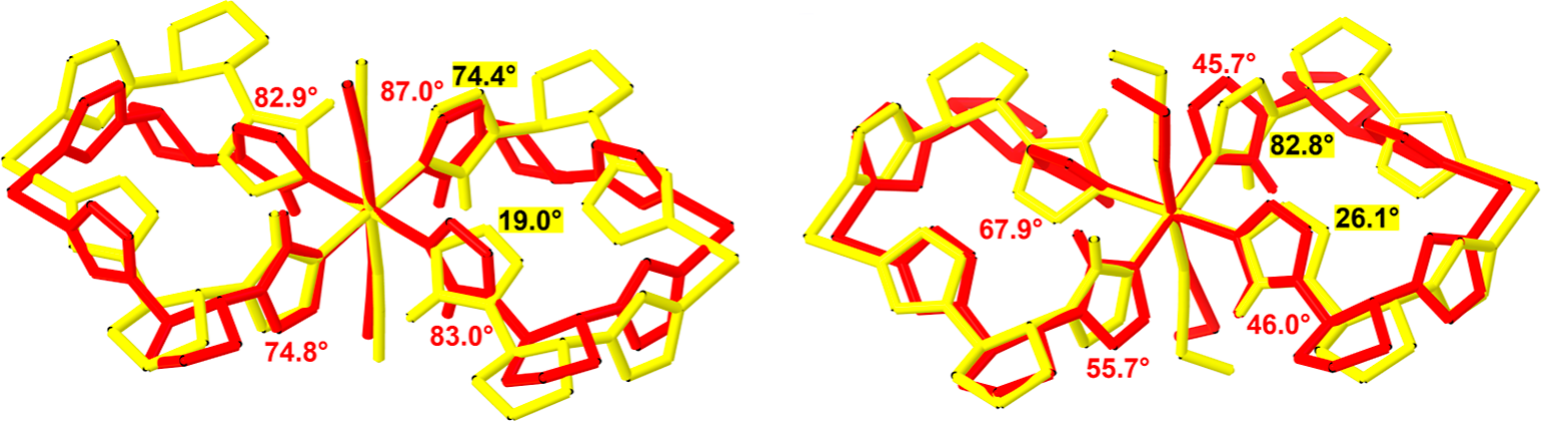
Differences between the conformation of the tetrazole rings in homochiral (shown with a red color) and heterochiral (yellow color) coordination compounds shown by overlaying fragments of the polycationic chain. Left: acetonitrile-based **1A** and **1B·solv**; right: propionitrile-based **2A** and **2B**. Hydrogen atoms (except for compared tetrazole groups), anions, and noncoordinated solvent molecules are omitted for clarity. Values for the dihedral angle between the equatorial coordination plane and plane of symmetry of the symmetry-independent tetrazole rings are presented.

This comparison leads to the conclusion that an exchange of one of the ligand molecules with its enantiomer (presynthetic “racemization” in a row **1B·solv** → **1A** and **2B** → **2A**) results in complete reorientation of one of the coordinated tetrazole rings with simultaneous retaining of the conformation by the rest of the ligand molecule (Figure 4). The rise of the temperature (which induces SCO in homochiral **1B·solv** and **2B**) does not lead to significant changes in values of the dihedral angles (Table 2).

**Figure 5:**
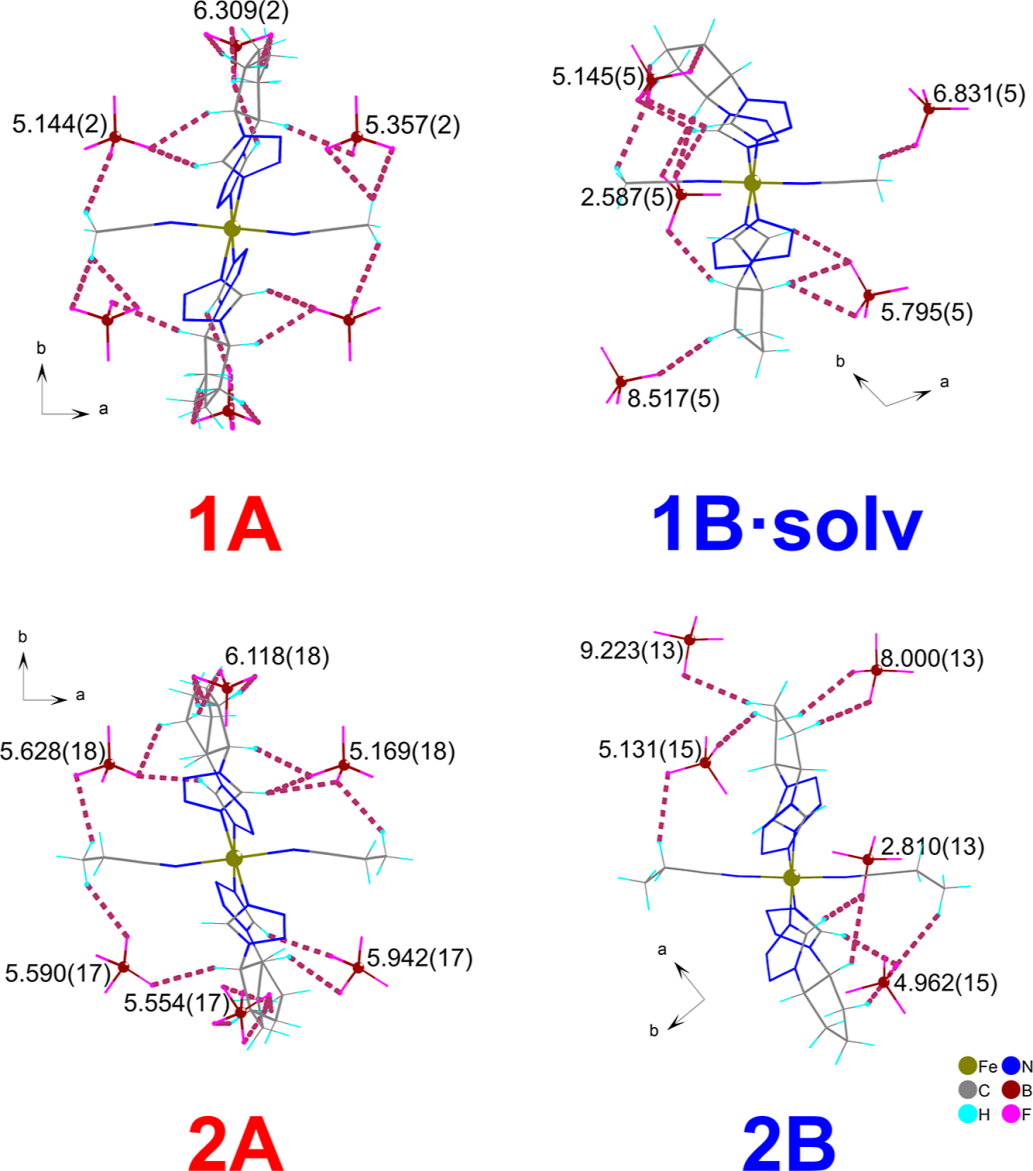
Comparison of the anion distribution around the polymeric unit in hetero- (**1A** and **2A**) and homochiral (**1B·solv** and **2B**) coordination compounds at 80 K, viewed along the polycationic chain ([001] direction). Distances are given in Å and were measured as the distance between the central boron atom of the corresponding anion and the “chain vector” defined by bridged iron(II) cations within the shown chain. Only anions forming weak direct contacts (shown with a thick plum dashed line) with polymeric units are taken into consideration. Noncoordinated acetonitrile molecules in the structure of **1B·solv** are omitted for clarity. The anions in the structure of **2A** are disordered between two positions; only half of them are shown for **2A** (half of the major and minor disordered anions, respectively) for clarity. Minor disordered fluorine atoms around common central boron atoms in the structure of **2B** are omitted for clarity.

“Racemization” of the double ligand bridge (Figure 2) by using heterochiral ligand **A** causes further consequences in the crystal structure of the obtained complexes than only the above-mentioned changes. Specifically, the distribution of noncoordinated tetrafluoroborate anions around polycationic chains strongly differs in homo- and heterochiral compounds (Figure 5).

Structural analysis can be started with a comparison of the distances between Fe(II) cations and the central atom of BF_4_^–^ anions. Table 1 presents the values of these distances determined for the asymmetric unit content. We noticed that the average distances between Fe(II) centers and boron atoms are shorter for the homochiral compounds (**1B·solv** and **2B**) exhibiting SCO in comparison to the racemic-based compounds (**1A** and **2A**) remaining in the HS form. Namely, at 80 K, the average Fe–B distances in SCO-active **1B·solv** and **2B** are equal to 5.184(4) and 5.043(10) Å, respectively, while in HS **1A** and **2A**, the corresponding values are equal to 6.177(2) and 6.099(11) Å, respectively. SCO phenomenon taking place in **1B·solv** and **2B** led to a significant increase in these distances, which are equal to 5.368(6) and 5.246(20) Å in the HS form of these compounds (determined in 250 and 310 K) for **1B·solv** and **2B**, respectively. Contrary to the aforementioned homochiral SCO-active complexes, the increase of temperature to 250 K did not cause considerable changes in these values in their heterochiral analogues **1A** and **2A**: the average Fe–B distances are equal to 6.255(3) and 6.121(17) Å, respectively (Table 1).

The next step of our structural analysis was quite obvious due to the 1D polymeric nature of the obtained coordination compounds. Namely, we compared the distribution of the BF_4_^–^ anions around the “chain vector” defined by bridged iron(II) cations (Figure 5). We considered only anions, which are located closest to the polycationic unit forming direct weak contacts with the chain (Tables S3–S6) and can be denoted as the “second coordination sphere”. The anions form numerous weak contacts with chains involving cyclopentane and tetrazole rings and methyl groups of coordinated nitrile molecules as donors of electron density. The characteristic distribution pattern was spotted by the naked eye when we considered the projection along the polycationic chain ([001] direction; Figure 5). Namely, the distribution of the counterions in racemic-based **1A** and **2A** can be referred to as “pseudohexagonal” (Figure 5, left), while in homochiral **1B·solv** and **2B**, the discussed anion distribution around the polymeric chain is quite irregular (Figure 5, right). These differences can be expressed by comparing distances between a central atom of BF_4_^–^ anions and chain vector at 80 K. Namely, for heterochiral compounds, these values lie in a range between 5.144(2) and 6.309(2) Å for **1A** and between 5.169(18) and 6.118(18) Å for **2A** (Figure 5, left). For homochiral complexes, the corresponding values at 80 K lie between 2.587(5) and 8.517(5) Å for **1B·solv** and 2.810(13) and 9.223(13) Å for **2B** (Figure 5, right). This quite big range underlines the irregular nature of anion distribution around polymeric units in homochiral complexes. Temperature-induced SCO (HS → LS) in these compounds leads to increasing of these distances.

It is supposed that SCO behavior is sensitive toward the electrostatic environment,^33,34^ so different anion arrangement in homo- and heterochiral systems can be an additional factor contributing to different properties.

In heterochiral systems **1A** and **2A**, all anions are engaged in the formation of a 3D network of intermolecular contacts. In the case of homochiral **1B·solv** and **2B**, only one of the crystallographically independent anions takes part in the formation of a 3D framework of weak intermolecular contacts, whereas the second one establishes weak interactions between 1D polymeric units extending into the 2D supramolecular structure. The detailed analysis of the weak interactions in the crystal structures of the complexes is presented in the Supporting Information.

### SCO in **1B**

Studies of the complexes revealed that the transition from hetero- to homochiral environments leads to serious reorganization of second coordination spheres. In other words, structural differences resulting from the presence of chiral or racemic molecules reflect themselves in crystal packing which involves a change of SCO properties in such a way that homochiral systems exhibit thermally induced SCO, whereas heterochiral ones remain in stable HS form. It concerns not only one pair (that is **2A** and **2B**) but also extends on acetonitrile derivatives **1A** and **1B·solv**. Thus, such a serious differentiation of SCO properties is more general and does not concern only one pair of compounds. In comparison with HS **1A** and **2A**, in which a pseudohexagonal arrangement of anions is observed, in SCO-active **1B·solv** and **2B**, the distribution of anions around the polymeric chains is very irregular. Such structural rebuilding also involves intermolecular interactions. There is no doubt that such differences may be responsible for the other properties. Nevertheless, there is a difference between **1B·solv** and **2B** depending on the presence of acetonitrile guest molecules in the former system, while the latter one does not contain solvent guest molecules. It is well known that the removal or exchange of guest molecules (that alters crystal packing) usually strongly affects SCO properties, and it often leads to the formation of SCO-inactive species. To verify whether the regularity of SCO property differentiation observed between homo- and heterochiral systems still exists for compounds of the same stoichiometry, we have decided to check the possibility to remove noncoordinated acetonitrile molecules from **1B·solv.**

**Figure 6:**
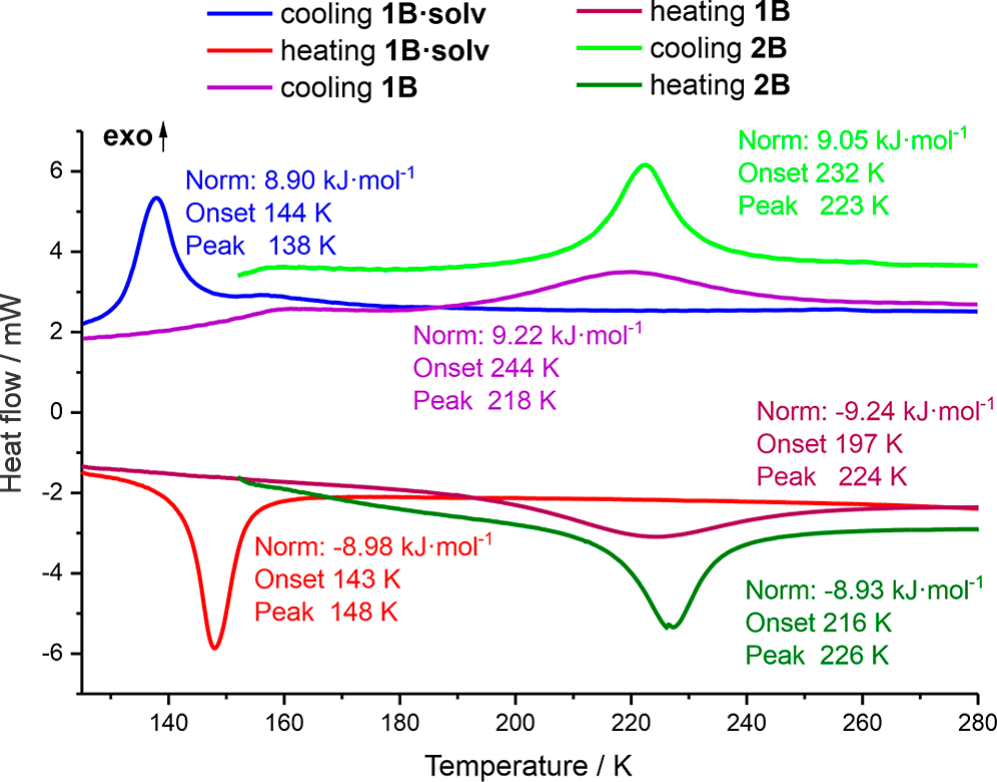
Thermograms recorded for **2B**, **1B·solv**, and **1B**.

Thermogravimetry analysis showed quite unexpected thermal stability of **1B·solv** because the removal of two acetonitrile guest molecules starts above 373 K (Figure S12). Just after leaving the acetonitrile molecules, subsequent heating triggers further degradation of the coordination compound. Therefore, to precisely undertake the process, we prepared a desolvated sample exploiting differential scanning calorimetry (DSC) monitoring. For this purpose, initial heat flow studies of **1B·solv** were carried out in the temperature range of 120–280 K (Figure 6) and revealed the presence of one well-developed peak in cooling and heating modes. Derived values of Δ*H* are in the expected range (10–20 kJ·mol^–1^) for Fe(II) SCO systems. In this temperature range, the liberation of noncoordinated solvent molecules does not occur.

Noteworthily, DSC studies of **2B** showed a similar picture; however, in comparison with **1B·solv**, peaks recorded in cooling and heating modes were shifted to higher temperatures remaining in agreement with the results of magnetic studies (Figure 1).

**Figure 7:**
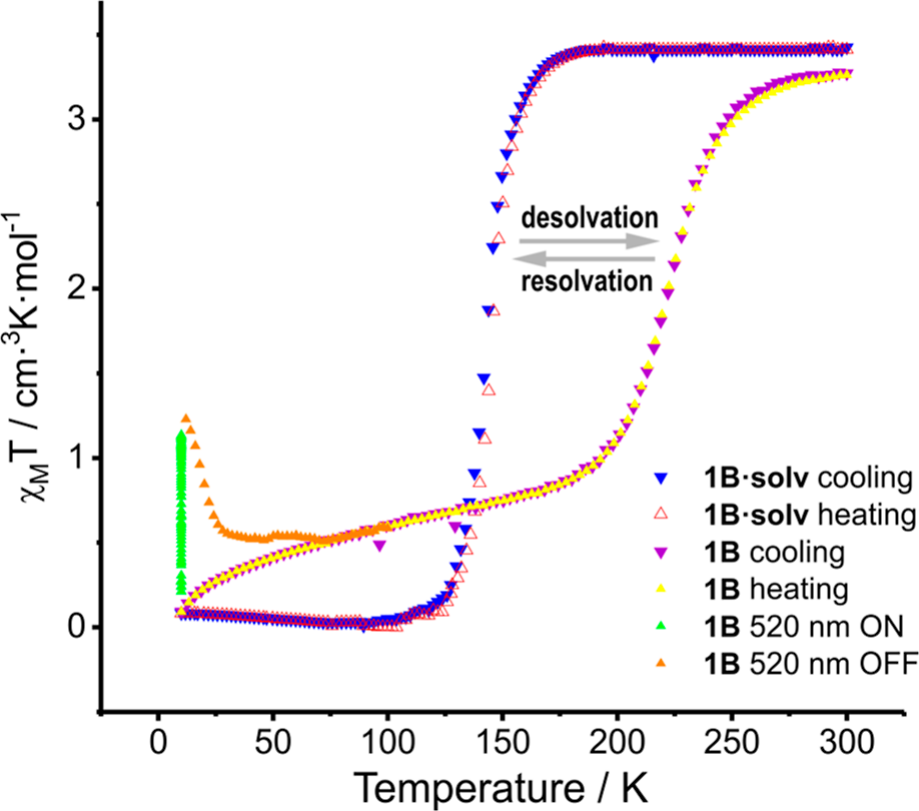
Changing of the χ_*M*_*T*(*T*) curve in **1B·solv** after desolvation. Resolvation of **1B** causes restoration of SCO properties (Figure S30) observed for **1B·solv**.

Several heating/cooling cycles were carried out, and in each subsequent cycle, the heating step temperature was increased by 10 °C in order to desolvate **1B·solv** (see the Supporting Information for the details). The procedure was repeated until the original peak disappeared. At the same time, the appearance of a novel peak was noticed in the temperature range of 220–240 K (Figures 6 and S13). To confirm an occurrence of SCO for samples prepared under DSC monitoring, magnetic studies of **1B** were carried out. Indeed, SCO occurs (Figure 7) in **1B**, which corresponds to noticed thermal anomaly recorded on DSC. Also, we have found the process of the desolvation to be reversible: wetting the desolvated sample of **1B** with acetonitrile restores the SCO curve (Figure S30) characteristic for the initial sample of **1B·solv**. Thus, the desolvated form **1B** also undergoes thermally induced SCO, which supports our speculation that strong differentiation of SCO properties between homo- and heterochiral systems results from structural differences enforced by “racemization”.

**Figure 8:**
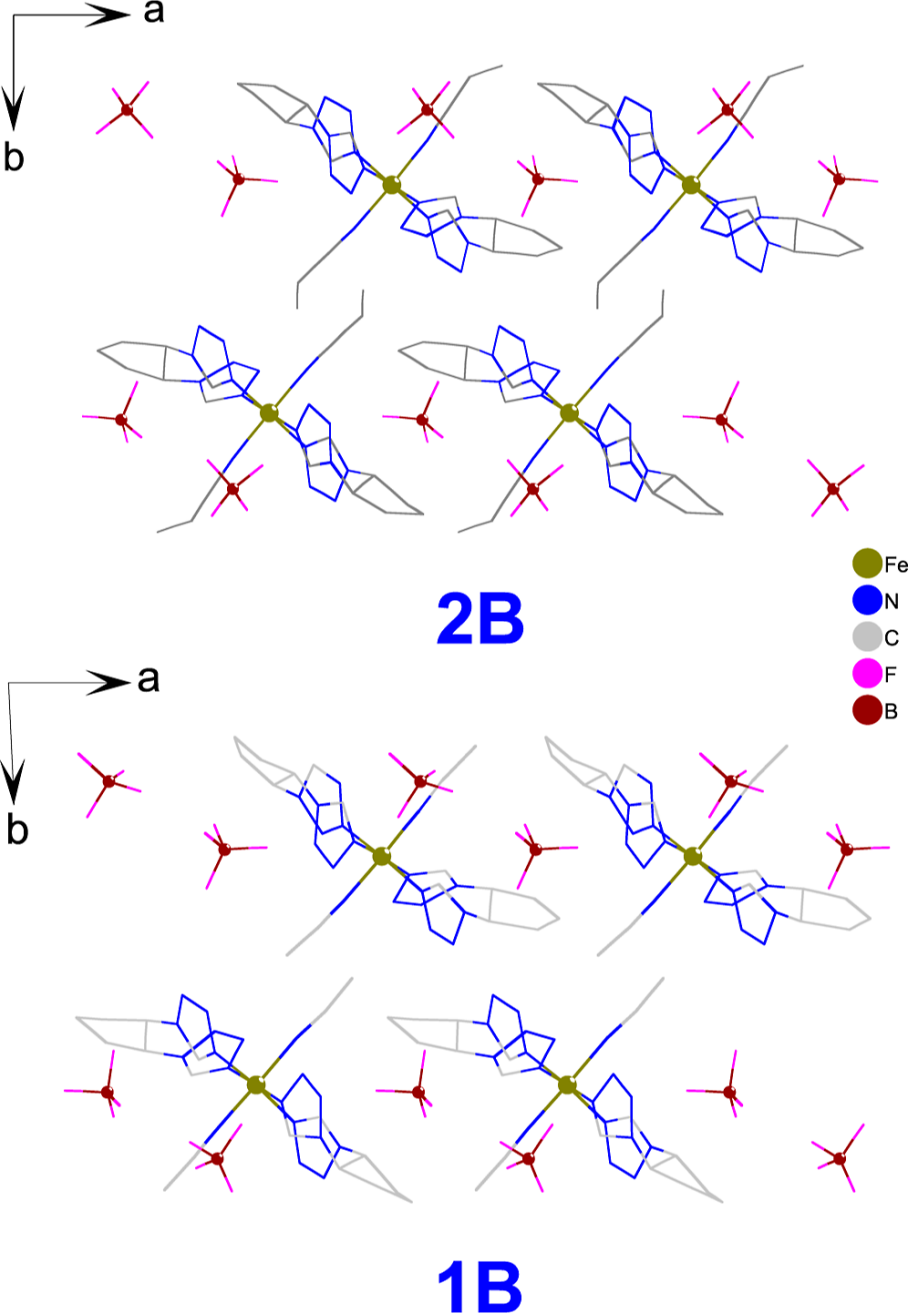
Comparison of crystal packing and anion arrangements in **2B** and **1B**.

Desolvation performed at a temperature of 375–380 K leads to powdering of the sample. However, careful examination of the desolvated sample revealed remnants of cracked and cloudy crystals. We were able to determine the crystal structure of **1B** only at 80 K (see the Supporting Information for more details). The collected diffraction data for the desolvated sample was quite poor and incomplete (because of the low quality of the desolvated crystals and their instability to the long exposure times of the X-ray radiation). However, we were able to solve and refine the tentative model for **1B** that clearly proved the lack of noncoordinated acetonitrile molecules in its crystal lattice. The space group did not change after the desolvation process (both **1B·solv** and **1B** crystallize in the *P*1 group); however, the volume corresponding to the one formal unit of the complex decreases by ca. 13% compared to the starting solvated form (see the Supporting Information for the cell parameter details), which is in good agreement with the nature of the desolvation process. It is worth noting that the irregular nature of the anion distribution around the polymeric unit observed for the homochiral **1B·solv** and **2B** (Figure 5) is preserved in the desolvated **1B**, too (Figure S14). Also, comparison of the crystal packing for **1B** and **2B** shows quite close resemblance (Figure 8).

It is interesting that besides **1B** and **2B** belonging to the different crystal systems (triclinic and orthorhombic, respectively), the unit cell parameters of the **1B** are close to the ones determined for **2B** at 80 K. Namely, the angles of the unit cell for **1B** are quite close to 90° [α = 87.63(2)°, β = 89.91(2)°, γ = 87.41(2)°], while the *a* and *c* vector lengths are very close to the corresponding values of **2B** [10.059(2) and 10.085(4), 8.736(1) and 8.763(4) Å, respectively; see the Supporting Information for details]. The length of the *b* vector of the **2B** unit cell vector is about two times longer than the corresponding vector of the **1B** unit cell [34.512(13) and 16.775(3) Å, respectively], which reflects the differences in symmetry of the corresponding space groups. Obviously, the unit cell parameters cannot have exactly the same values for the **1B** and **2B** unit cells because they contain different aliphatic groups attached to the nitrile donor (methyl and ethyl, respectively), but observed similarities only underline the similar nature of this pair of the complexes, which was discussed above.

Irradiation of **1B** with green light induces partial LS → HS switching; however, switching the light off involves immediately back to LS form (Figure 7). Greater stability of the metastable HS* state of **1B·solv** in relation to the desolvated form **1B** remains in agreement with the inverse energy gap law.^35^

## Summary

Optically pure (*RR*)-*trans*-1,2-di(tetrazol-1-yl)cyclopentane and a (*RR*/*SS*) racemic mixture (L) were obtained and used to prepare Fe(II) coordination compounds. In both cases, the complexes belong to 1D coordination polymers, in which neighboring Fe(II) ions are bridged by two ligand molecules. The compounds represent rare examples of metal complexes, in which the first coordination sphere of the central ion is formed not only by tetrazol-1-yl rings but also with axially coordinated nitrile molecules. It was established that “polymeric tecton” [Fe(L)_2_Fe]_*n*_^4+^ is stable toward nitrile exchange, which was exploited to perform comparative studies on structural aspects of thermally induced SCO in enantiomerically pure and racemic coordination compounds. It was found that an application of optically pure (*RR*)-*trans*-1,2-di(tetrazol-1-yl)cyclopentane or (*RR*/*SS*) racemic mixture allows manipulation of an ability to thermally induce crossover. Whereas homochiral 1D coordination polymer [Fe(L)_2_(C_2_H_5_CN)_2_](BF_4_)_2_ exhibits SCO, the heterochiral system remains in HS form down to 10 K. Such a fundamental differentiation is more general because an exchange of an axially coordinated propionitrile coligand with acetonitrile still results in complete property differentiation for both types of complexes. The source of this regularity is the different structures of the polymeric chains in the homo- and heterochiral systems, resulting in changes in the overall coordination environment. It turns out that replacing half of the *RR* molecules with their *SS* enantiomer enforces significant changes both in the first and further coordination spheres. In particular, this leads to a completely different arrangement of counterions around the polymeric unit and the rebuilding of the intermolecular interaction network. We suppose that an ability to preserve the structure and magnetic properties makes “polymeric tecton” [Fe(L)_2_Fe]_*n*_^4+^ a convenient platform for studies on the introduction of other functionalities sensitive to chirality and spin-state differentiation in the form of nitrile derivative coligands.

## Experimental Section

### Materials and Methods

Sodium azide (Fluka, >99%), acetic acid (Baker, 99–100%), methanol (Honeywell, HPLC ≥99.9%), acetonitrile (Baker, HPLC), methanol-d4 (Sigma-Aldrich, ≥ 99.8% atom D), and iron(II) tetrafluoroborate hexahydrate (Sigma-Aldrich) were used as delivered without further purification. Triethyl orthoformate (Riedel-de Haën, 99%) and CH_2_Cl_2_ (Baker) were distilled prior to use. Acetonitrile (Baker, HPLC grade) and propionitrile (Sigma-Aldrich, purum ≥99.0%) used in the syntheses of the coordination compounds were distilled over CaH_2_ prior to use. Syntheses of iron(II) complexes were carried out under a nitrogen atmosphere using the standard Schlenk technique. *Caution! Even though no problems were encountered, it is worth mentioning that complexes containing tetrazole derivatives are potentially explosive and should be synthesized in small scale.*

NMR spectra were recorded on a Bruker AVANCE III 500 MHz spectrometer at 298 K. Elemental analyses for carbon, hydrogen, and nitrogen were performed on a CHNS vario EL III (Elementar) analyzer. IR spectra of the ligands were recorded with an Alpha II FT-IR (Bruker) spectrometer with a diamond ATR crystal. IR spectra of the coordination compounds were recorded as Nujol mulls with the 5600 FT-IR (Jasco) spectrometer in the range of 400–4000 cm^–1^. Temperature-dependent measurements of the magnetic susceptibility of the complexes were carried out with a Quantum Design SQUID magnetometer (MPMS-XL-5) under a 1 T applied magnetic field. Measurements were carried out with a rate of 1 K min^–1^ (cooling and heating). Magnetic data were corrected for the signal of the glass tube and for the diamagnetic contributions of the sample, which were estimated from Pascal’s constants. DSC measurements were carried out with a Mettler Toledo DSC at 10 K min^–1^. Thermogravimetry analysis was performed using a SETARAM SETSYS 16/18 at 10 K min^–1^ under a N_2_ atmosphere.

### Ligand Preparation

2.00 g portion (11.6 mmol, 1.00 equiv) of racemic (*RR*/*SS*)-cyclopentane-1,2-diaminium dihydrochloride and 2.01 g (30.9 mmol, 2.66 equiv) of sodium azide were placed in a round-bottom flask (25 mL). Then, 8.20 mL (49.3 mmol, 4.25 equiv) of triethyl orthoformate and 6.0 mL of glacial acetic acid were added to the flask. The obtained white suspension was vigorously stirred at 35 °C for 42 h. Then, the mixture was heated at reflux for 24 h. After that, the reaction mixture was cooled to RT and 10 mL of concentrated hydrochloric acid was added dropwise. The white precipitate was filtered off under reduced pressure and washed with hot acetonitrile (2 × 10 mL). The filtrate was evaporated under reduced pressure affording 2.40 g of brown solid. Desired product (*RR*/*SS*)-1,2-di(tetrazol-1-yl)cyclopentane (**A**) was isolated from this solid using flash chromatography (silica gel 0.040–0.063 mm; CH_2_Cl_2_/CH_3_CN/CH_3_OH 10/1/0.3; *R*_f_ = 0.36) as colorless crystals. Yield: 1.40 g (58%). Elemental analysis CHN: calcd (C_7_H_10_N_8_) C (40.8%), N (54.3%), H (4.9%); found C (40.6%), N (54.4%), H (5.0%). ^1^H NMR (CD_3_OD, 500 MHz, 298 K) δ: 9.26 [s, 1H, Tz (C–*H*)], 5.63–5.57 [m, 2H, Cp (C*H*)], 2.74–2.66 [m, 2H, diaster. Cp (C*H*_2_)], 2.48–2.39 [m, 2H, diaster. Cp (C*H*_2_)], 2.30–2.23 [m, 2H, Cp (C*H*_2_)] ppm (Figure S1). ^13^C NMR (CD_3_OD, 126 MHz, 298 K) δ: 144.8 (*C*-H_Tz_), 66.0, 32.5, 23.0 ppm (Figure S2). FT-IR (ATR, 298 K) spectrum: see Figure S15.

The same procedure was exploited in the synthesis of an enantiomeric pure ligand starting from (*RR*)-cyclopentane-1,2-diaminium dihydrochloride. Yield of desired product (*RR*)-1,2-di(tetrazol-1-yl)cyclopentane (**B**): 1.15 g (48%). Elemental analysis CHN: calcd (C_7_H_10_N_8_) C (40.8%), N (54.3%), H (4.9%); found C (40.8%), N (54.1%), H (5.1%). ^1^H NMR (CD_3_OD, 500 MHz, 298 K) δ: 9.26 (s, 1H, C–*H*_Tz_), 5.63–5.57 [m, 2H, Cp (C*H*)], 2.74–2.66 [m, 2H, diaster. Cp (C*H*_2_)], 2.48–2.39 [m, 2H, diaster. Cp (C*H*_2_)], 2.30–2.23 [m, 2H, Cp (C*H*_2_)] ppm (Figure S3). ^13^C NMR (CD_3_OD, 126 MHz, 298 K) δ: 144.8 (*C*–H_Tz_), 66.0, 32.5, 23.0 ppm (Figure S4). FT-IR (ATR, 298 K) spectrum: see Figure S6.

### Coordination Compound Preparation

All syntheses of coordination compounds were performed using the standard Schlenk technique under a N_2_ atmosphere at room temperature.

#### Synthesis of **1A**

144 mg portion (0.700 mmol, 2.00 equiv) of racemic (*RR*/*SS*)-1,2-di(tetrazol-1-yl)cyclopentane was dissolved in 20 mL of acetonitrile. Then, 118 mg (0.350 mmol, 1.00 equiv) of Fe(BF_4_)_2_·6H_2_O was added affording a colorless solution. The next day, the volume of the obtained solution was reduced by ca. 30% under N_2_. After 10 days, the desired crystalline product was obtained in the form of colorless blocks. It was filtered off, washed with acetonitrile, and dried under N_2_. Yield: 145 mg (57%). Elemental analysis CHN: calcd (C_18_H_26_B_2_F_8_FeN_18_) C (29.9%), N (34.8%), H (3.6%); found C (29.8%), N (35.0%), H (3.4%). FT-IR (298 K) spectrum: see Figure S7. The same procedure was exploited in the synthesis of **1B·solv** using heterochiral (*RR*)-1,2-di(tetrazol-1-yl)cyclopentane, which was obtained in the form of colorless prisms. Yield: 76.3 mg (30%). Elemental analysis CHN: calcd (C_22_H_32_B_2_F_8_FeN_20_) C (32.8%), N (34.8%), H (4.0%); found C (32.8%), N (34.6%), H (4.3%). FT-IR (Nujol mull, KBr windows, 298 K) spectrum: see Figure S9.

**1B** was obtained by desolvation of **1B·solv**: 15.8 mg of **1B·solv** was slowly (1 K min^–1^) heated to 385 K starting from 293 K under a nitrogen atmosphere, and the sample was kept at 385 K for 10 min. After that, the desolvated sample was slowly (1 K min^–1^) cooled back to 293 K. The desired product was obtained as a pale-pink polycrystalline powder. Yield: 14.0 mg (99%). Elemental analysis CHN: calcd (C_18_H_26_B_2_F_8_FeN_18_) C (29.9%), N (34.8%), H (3.6%); found C (29.7%), N (34.9%), H (3.4%). FT-IR (Nujol mull, KBr windows, 298 K) spectrum: see Figure S10.

#### Synthesis of **2A**

41.2 mg portion (0.200 mmol, 2.00 equiv) of racemic (*RR*/*SS*)-1,2-di(tetrazol-1-yl)cyclopentane was dissolved in 4.0 mL of propionitrile. Then, 33.8 mg (0.100 mmol, 1.00 equiv) of Fe(BF_4_)_2_·6H_2_O dissolved in 4.0 mL of propionitrile was added, affording a colorless solution. The next day, the volume of the obtained solution was reduced by ca. 10% under N_2_. After 30 days, the desired crystalline product was obtained in the form of colorless blocks. It was filtered off, washed with propionitrile, and dried under N_2_. Yield: 38.4 mg (50%). Elemental analysis CHN: calcd (C_20_H_30_B_2_F_8_FeN_18_) C (31.9%), N (33.5%), H (4.0%); found C (31.7%), N (33.7%), H (3.9%). FT-IR (Nujol mull, KBr windows, 298 K) spectrum: see Figure S8. The same procedure was exploited in the synthesis of **2B** using heterochiral (*RR*)-1,2-di(tetrazol-1-yl)cyclopentane, which was obtained in the form of thin pale-pink needles. Yield: 16.9 mg (22%). Elemental analysis CHN: calculated (C_20_H_30_B_2_F_8_FeN_18_) C (31.9%), N (33.5%), H (4.0%); found C (32.0%), N (33.7%), H (4.3%). FT-IR (Nujol mull, KBr windows, 298 K) spectrum: see Figure S11.

### X-ray Data Collection and Structure Determination

Both ligands (**A** and **B**) were dissolved in CH_3_OH at RT, and X-ray quality crystals of the ligands were obtained after slow evaporation of the mother liquor at RT. The crystals of complexes **1A–2B** suitable for X-ray measurements were obtained directly from the syntheses of macroscopic samples and preselected using a stereoscopic microscope. The crystal of **1B** was selected from the desolvated macroscopic sample (see the Supporting Information for details). For the X-ray measurements, single crystals of the ligands or complexes were placed onto a glass capillary in the drop of oil. The crystallographic measurements were performed on an XtaLAB Synergy R DW (Rigaku) κ-geometry four-circle diffractometer equipped with a Hybrid Pixel Array Detector 150° (Rigaku) using CuKα radiation (λ = 1.5418 Å) or MoKα radiation (λ = 0.71073 Å). Low temperatures were achieved with a stream of cold nitrogen gas (Oxford Cryosystems cooling device); the temperature stability was 0.1 K. The crystallographic measurements for ligands **A** and **B** were performed at 100 K. Crystal structures of **1A** and **2A** were determined at 250 and 80 K. The crystal structure of **1B** was determined at 80 K. Variable-temperature SC-XRD measurements of **1B·solv** and **2B** were performed in a cooling (250 → 80 K and 310 → 80 K, respectively) mode. Data reduction and analysis including absorption corrections were performed with the CrysAlisPro^36^ software. The crystal structures were solved by dual-space recycling with the SHELXT-2018^37^ program. The refinements were carried out with the SHELXL-2018/3^38^ program using the full-matrix least-squares method. Hydrogen atoms were included from geometric considerations. The absolute configuration of the crystals of chiral compounds **B**, **1B·solv**, **1B**, and **2B** was determined based on the absolute configuration of the (*trans*)-1,2-disubstituted-cyclopentane moiety and confirmed by anomalous scattering and the Flack parameter values. In the final refinement stages, a riding model was used for C–H bonds. During the refinement of the disordered cyclopentane ring, tetrafluoroborate anion constraints on atomic displacement parameters (EADP instruction) and geometric restraints (SADI, SAME instructions) were used. The crystal structures of **1B·solv 1B** and **2B** were solved in a nonstandard setting, so that the unit cell vector [001] was aligned with the polymeric chains in all described structures. The crystal data and experimental and refinement details are given in the Supporting Information as well as in the crystallographic information file (CIF) deposited in the Cambridge Crystallographic Data Centre (CCDC 2364829–2364838). These data can be obtained free of charge from The Cambridge Crystallographic Data Centre via www.ccdc.cam.ac.uk/data_request/cif. Diamond^39^ and Olex^40^ programs were used to make the figures, and the Platon^41^ program was used for geometric analysis of the weak interactions. The structure of **1B** was not deposited in the CCDC due to the low quality of the starting data set. The structure of **1B** is attached as a Supporting Information file and should be treated only as a tentative model.

## Supporting Information Available

^1^H and ^13^C NMR and IR spectra of ligands; IR spectra of complexes; thermogravimetric analysis of complexes; DSC experiment for desolvation of **1B·solv**; detailed crystal structure information for ligands and complexes; detailed discussion concerning intermolecular contacts in structures of complexes; and comparison of χ_*M*_*T*(*T*) dependence for initial **1B·solv** and after wetting desolvated sample **1B** with acetonitrile (PDF)

## Abbreviations

**A**: racemic (*RR*/*SS*)-1,2-di(tetrazol-1-yl)cyclopentane
**B**: (*RR*)-1,2-di(tetrazol-1-yl)cyclopentane
**1A**: complex [Fe((*RR*/*SS*)-C_7_H_10_N_8_)_2_(CH_3_CN)_2_](BF_4_)_2_
**1B·solv**: complex [Fe((*RR*)-C_7_H_10_N_8_)_2_(CH_3_CN)_2_](BF_4_)_2_·2CH_3_CN
**1B**: complex [Fe((*RR*)-C_7_H_10_N_8_)_2_(CH_3_CN)_2_](BF_4_)_2_
**2A**: complex [Fe((*RR/SS*)-C_7_H_10_N_8_)_2_(C_2_H_5_CN)_2_](BF_4_)_2_
**2B**: complex [Fe((*RR*)-C_7_H_10_N_8_)_2_(C_2_H_5_CN)_2_](BF_4_)_2_
