# Supplementary material for: Spin Crossover Quenching by “Racemization” in a Family of trans-1,2-Di(tetrazol-1-yl)cyclopentane-Based Fe(II) 1D Coordination Polymers
Source: Inorg Chem. 2024 Sep 12;63(38):17762–73. doi: 10.1021/acs.inorgchem.4c02671 (PMC11423399; doi:10.1021/acs.inorgchem.4c02671)
Supplement: Supplementary file 1 — ic4c02671_si_001.pdf [file ic4c02671_si_001.pdf]

## Supplementary Information

# Spin crossover quenching by “racemization” in a family of *trans*-1,2-di(tetrazol-1-yl)cyclopentane-based Fe(II) 1D coordination polymers

*Vladyslav Maliuzhenko,[a]* Marek Weselski,[a] Janusz Gregoliński,[a]*
*Maria Książek,[b] Joachim Kusz,[b] Robert Bronisz[a]**

[a] *Faculty of Chemistry, University of Wrocław, F. Joliot-Curie 14, 50-383, Wrocław, Poland*

[b] *Institute of Physics, University of Silesia, 75 Pułku Piechoty 1, 41-500 Chorzów, Poland*

*vladyslav.maliuzhenko@uwr.edu.pl*

*robert.bronisz@uwr.edu.pl*

# Table of contents

NMR spectra of the obtained ligands ........................................................................ 3
IR spectra of the ligands and coordination compounds ....................................................... 7
Thermogravimetric analysis of the coordination compounds ............................................. 13
DSC monitoring of the desolvation process of 1**B·solv** ..................................................... 14
Crystal data and experimental and refinement details ........................................................ 16
Crystal data for **1B** and experimental and refinement details ............................................. 18
Geometric parameters of the weak interactions in the crystal structures of the coordination compounds ........................................................................................................ 21
General description of the crystal structures of the ligands ................................................ 26
General description of the crystal structures of the coordination compounds .................... 28
Detailed analysis of the weak interactions in the crystal structures of the coordination compounds ........................................................................................................ 30
Comparison of $\chi_M T(T)$ dependences for initial **1B·solv** and after wetting desolvated sample **1B** with acetonitrile ........................................................................................ 43

## NMR spectra of the obtained ligands

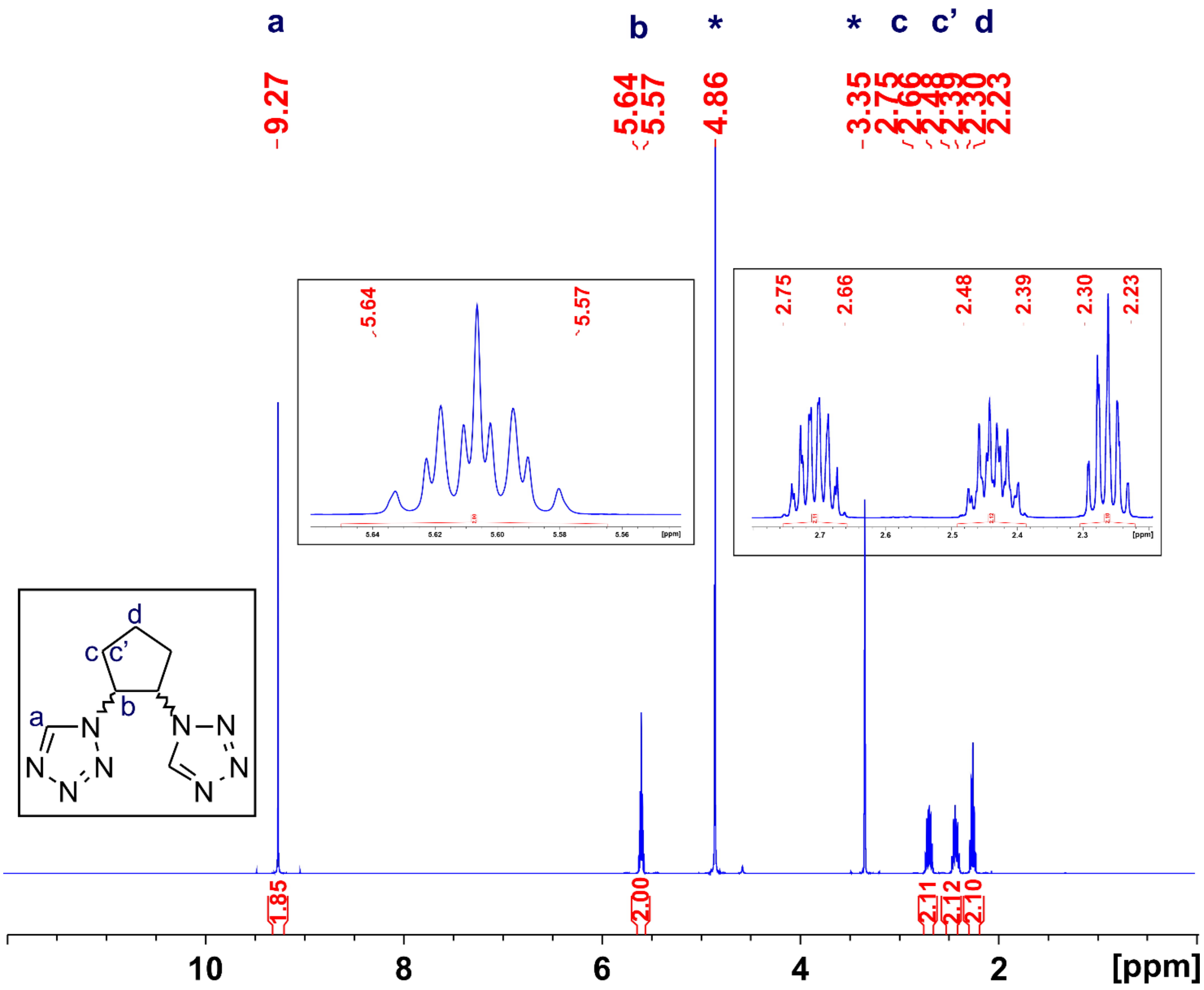


**Figure SI 1.** $^{1}H$ NMR ($CD_3OD$, 298 K, 500 MHz) spectrum recorded for the *trans*-(*RR*/*SS*)-1,2-di(tetrazol-1-yl)cyclopentane (racemic ligand **A**). Solvent residual signal is shown with *****.

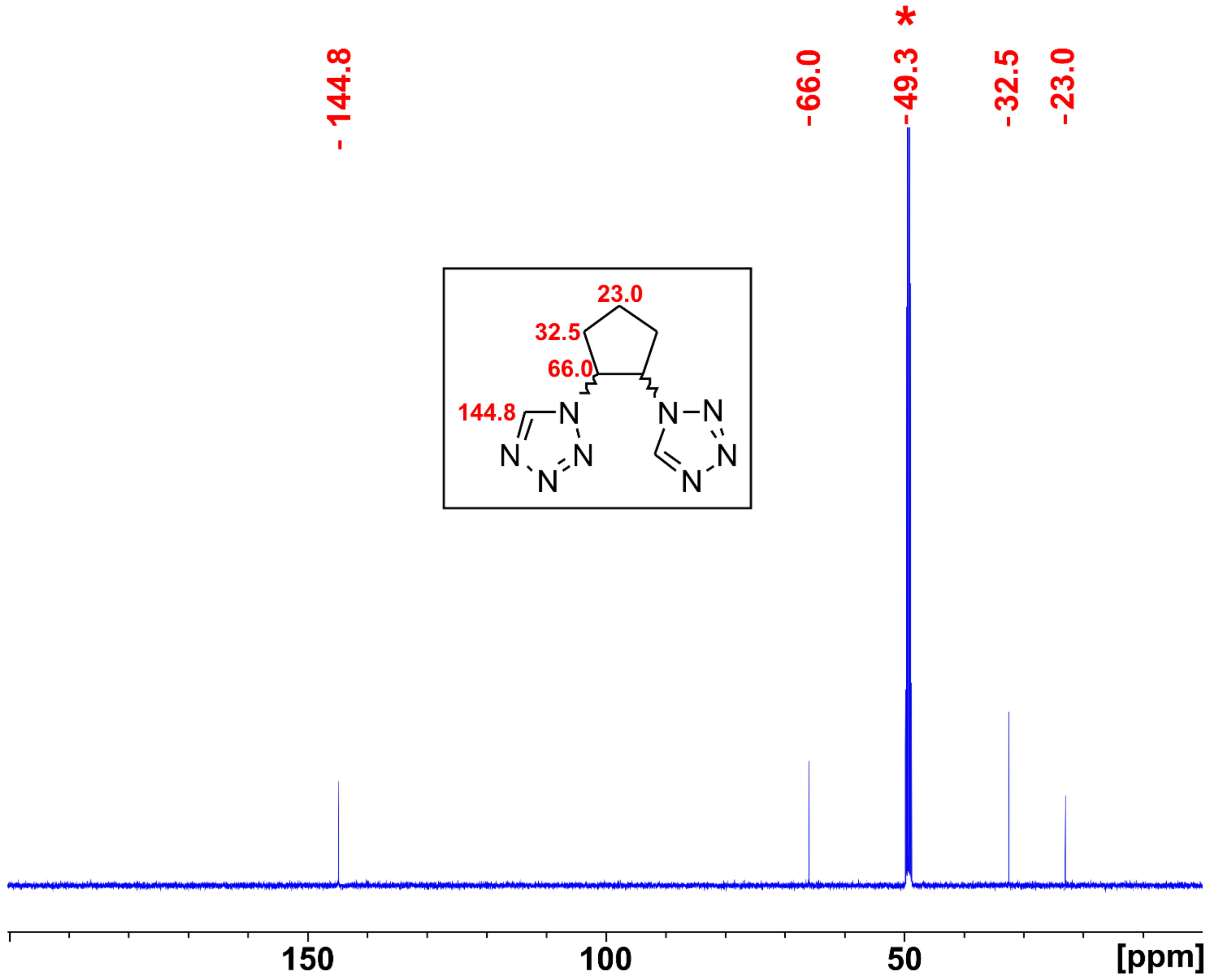


**Figure SI 2.** $^{13}C$ NMR ($CD_3OD$, 298 K, 126 MHz) spectrum recorded for the *trans*-(*RR*/*SS*)-1,2-di(tetrazol-1-yl)cyclopentane (racemic ligand **A**). Solvent residual signal is shown with *.

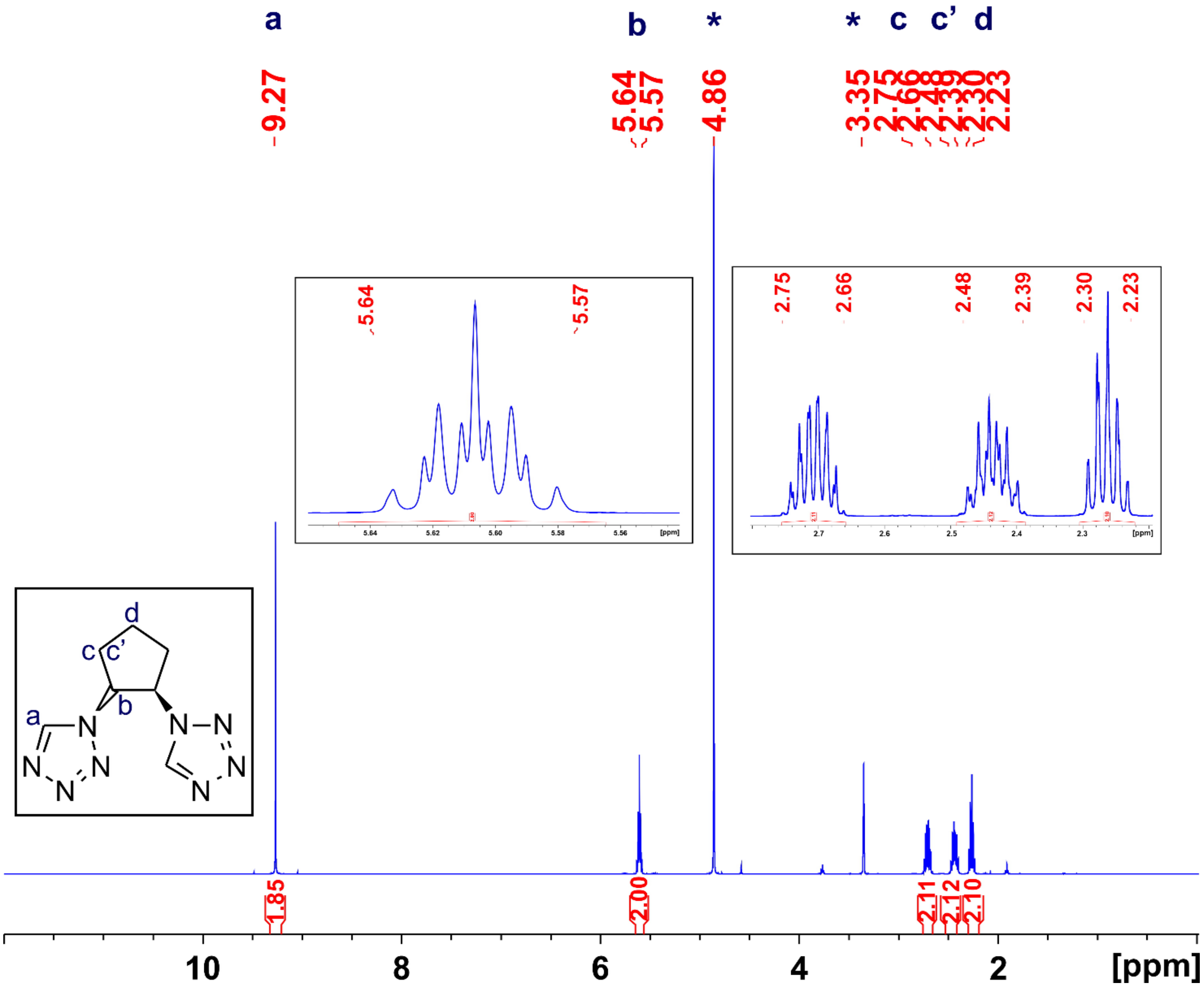


**Figure SI 3.** $^{1}$H NMR ($CD_3OD$, 298 K, 500 MHz) spectrum recorded for the *trans*-(*RR*)-1,2-di(tetrazol-1-yl)cyclopentane (homochiral ligand **B**). Solvent residual signal is shown with *****.

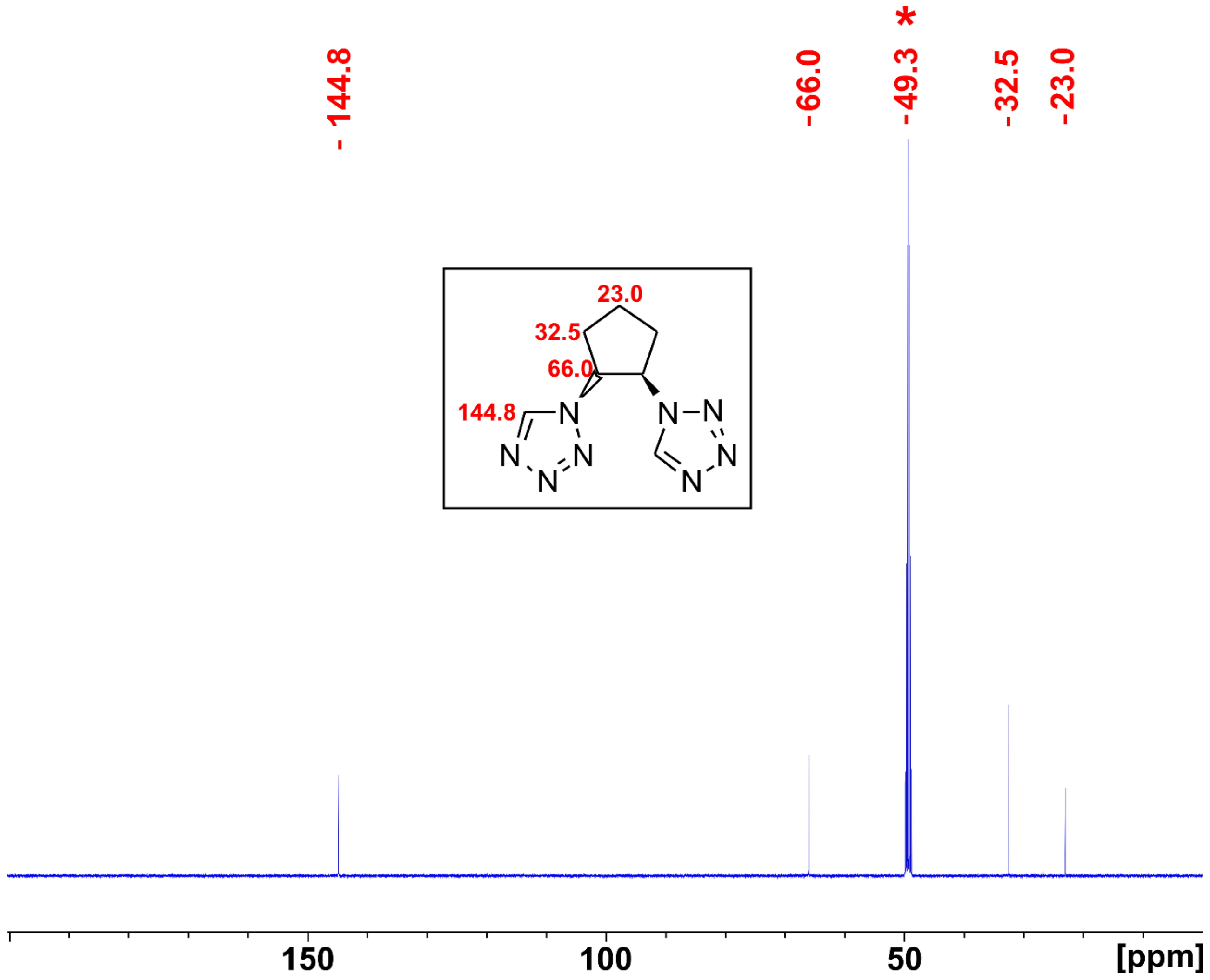


**Figure SI 4.** $^{13}C$ NMR ($CD_3OD$, 298 K, 126 MHz) spectrum recorded for the *trans*-(*RR*)-1,2-di(tetrazol-1-yl)cyclopentane (homochiral ligand **B**). Solvent residual signal is shown with *****.

# IR spectra of the ligands and coordination compounds

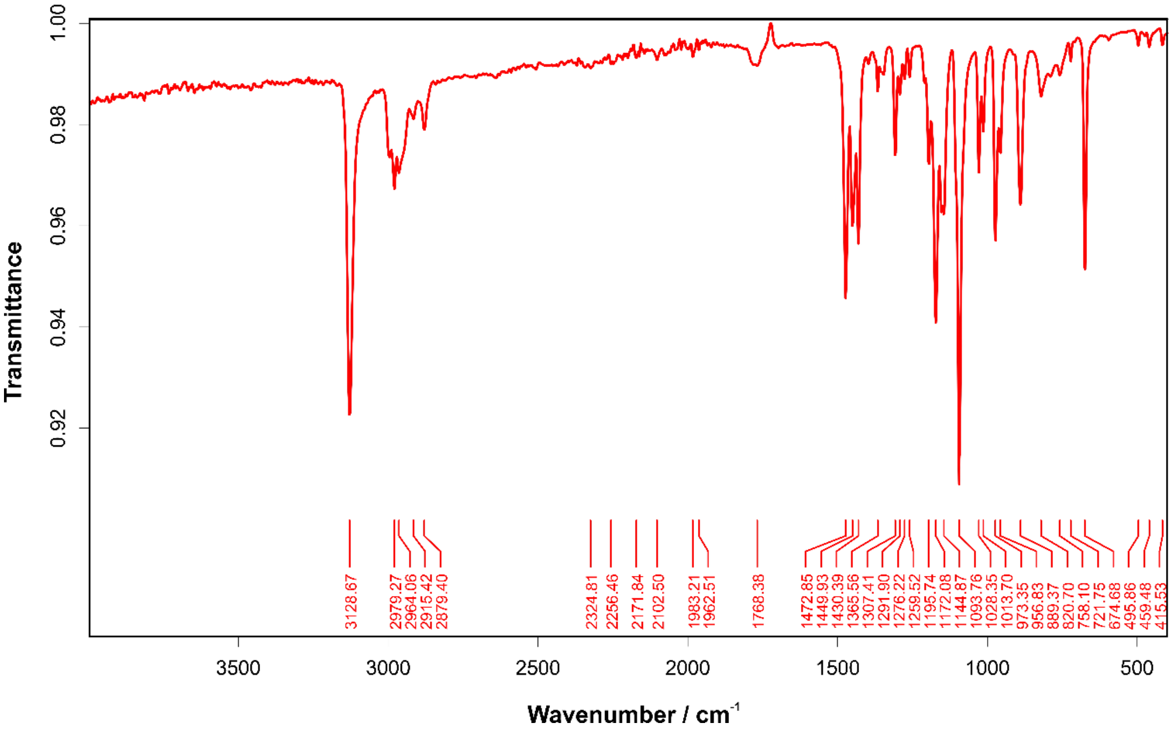


**Figure SI 5.** FT-IR (ATR; 298 K) spectrum recorded for the *trans*-(*RR*/*SS*)-1,2-di(tetrazol-1-yl)cyclopentane (racemic ligand **A**).

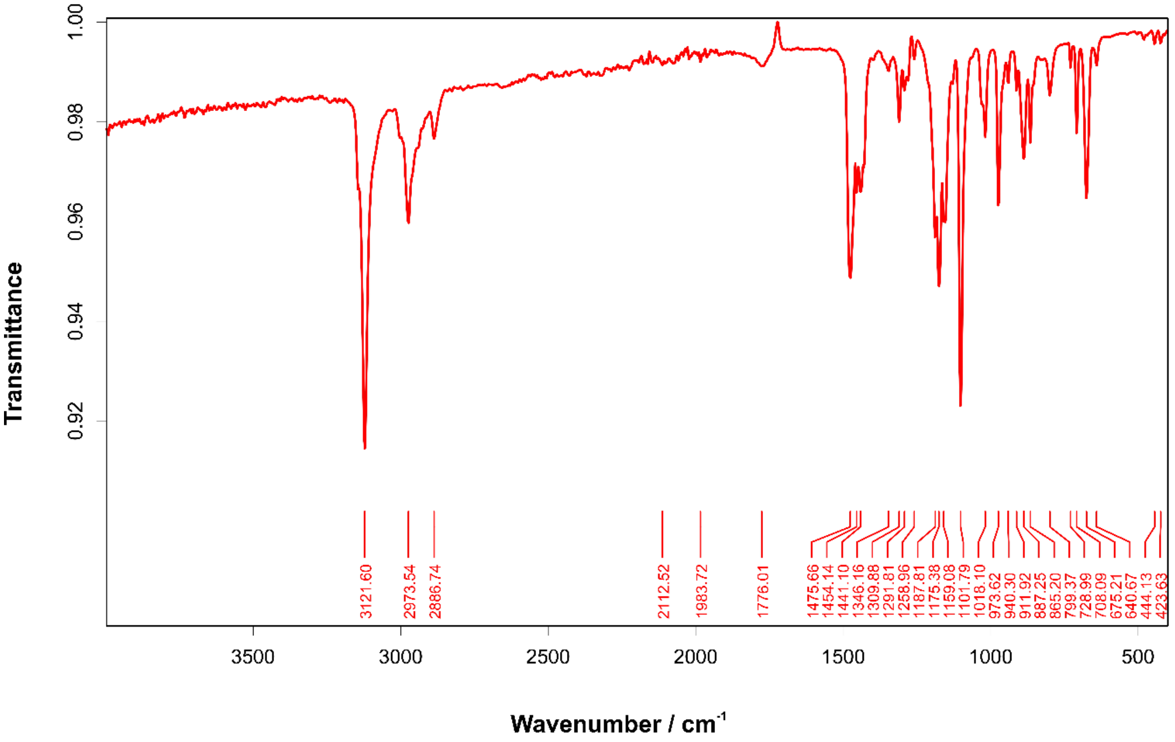


**Figure SI 6.** FT-IR (ATR; 298 K) spectrum recorded for the *trans*-(*RR*)-1,2-di(tetrazol-1-yl)cyclopentane (homochiral ligand **B**).

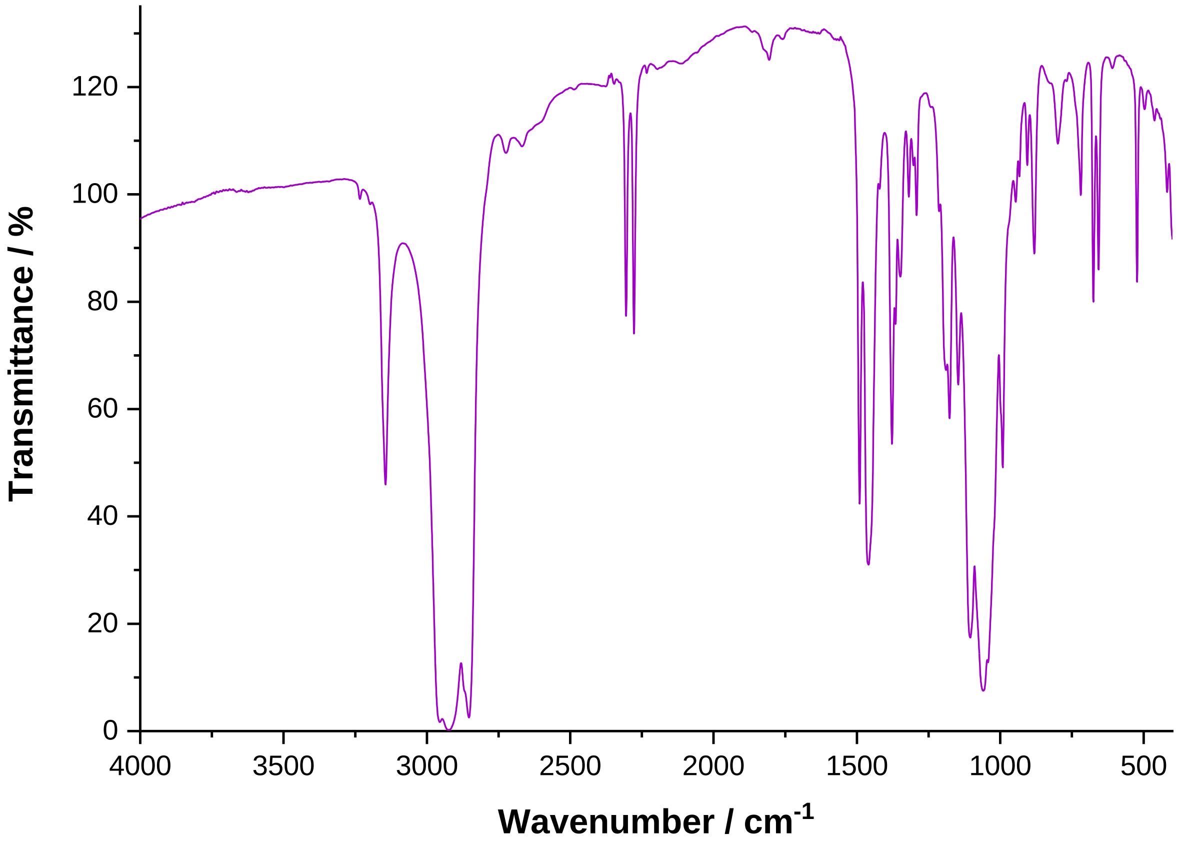


**Figure SI 7.** FT-IR (Nujol mull, KBr windows, 298 K) spectrum recorded for the heterochiral coordination compound **1A**.

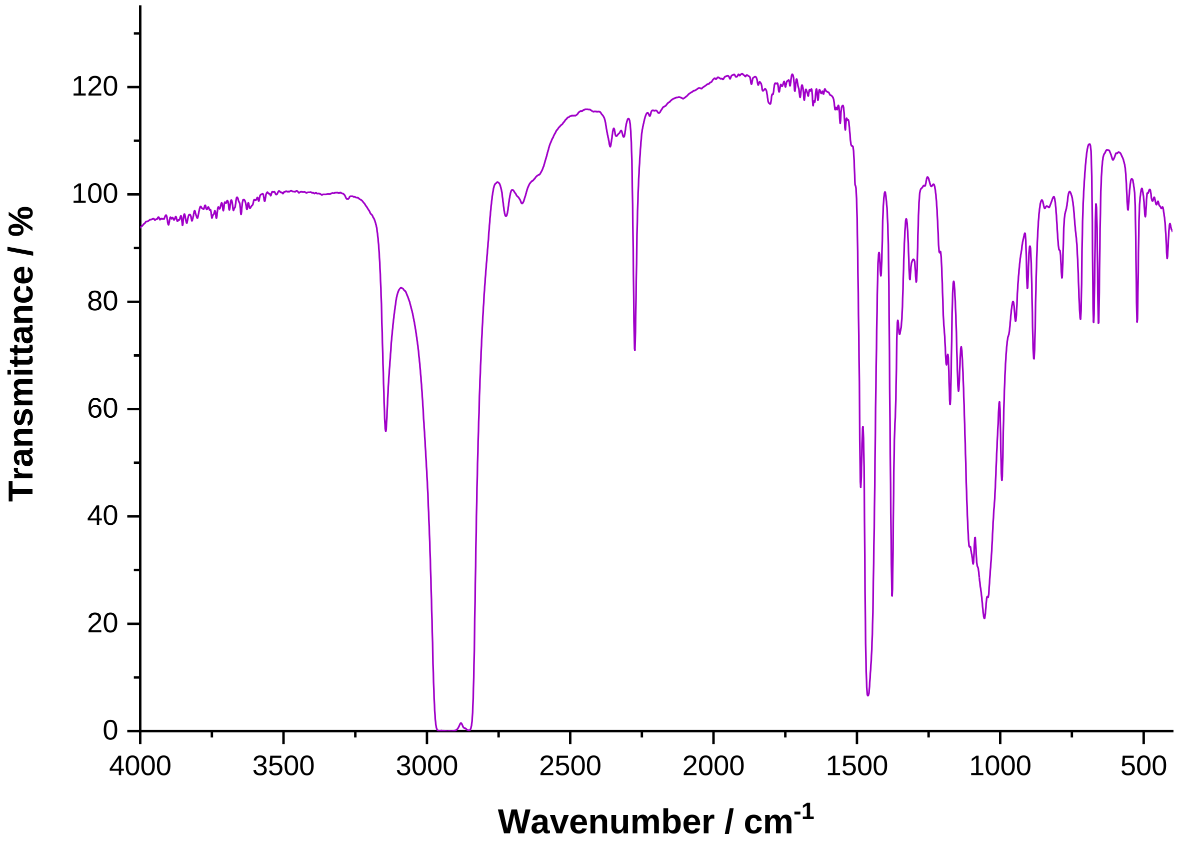


**Figure SI 8.** FT-IR (Nujol mull, KBr windows, 298 K) spectrum recorded for the heterochiral coordination compound **2A**.

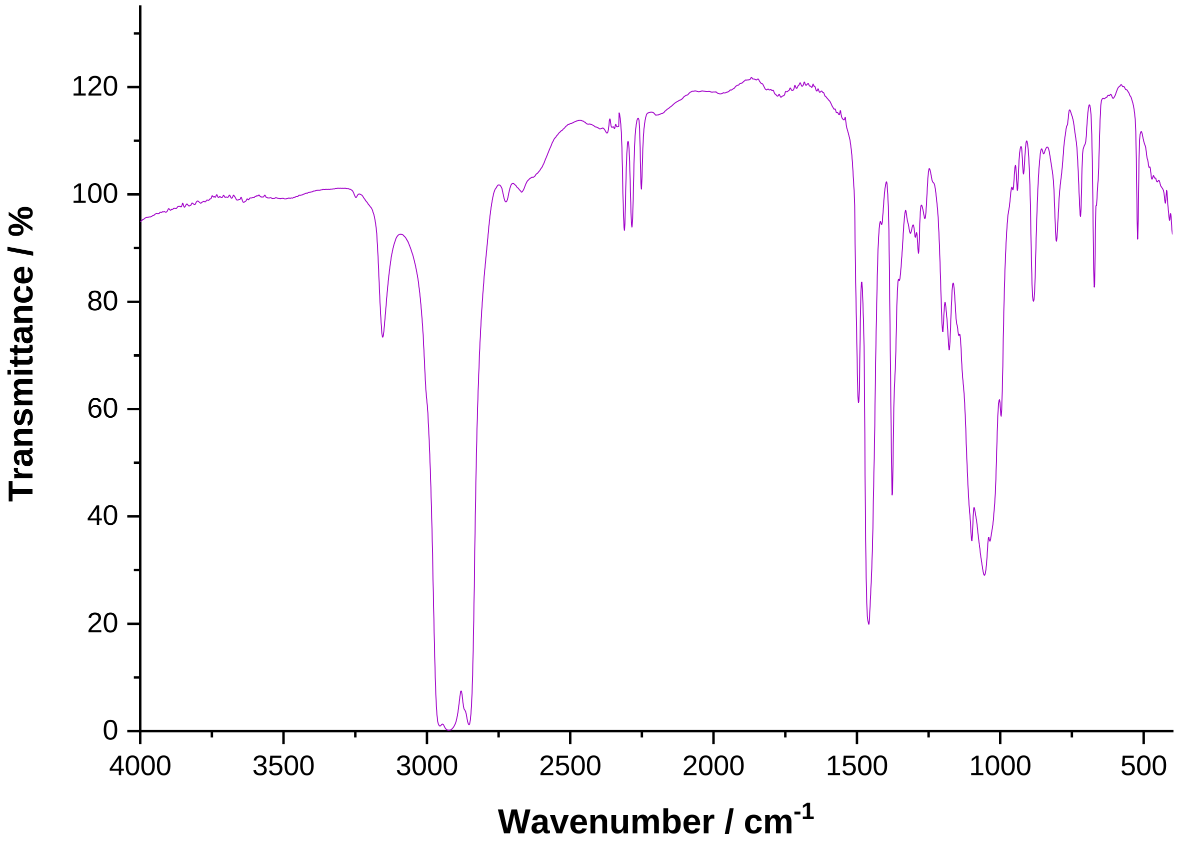


**Figure SI 9.** FT-IR (Nujol mull, KBr windows, 298 K) spectrum recorded for the homochiral coordination compound **1B·solv**.

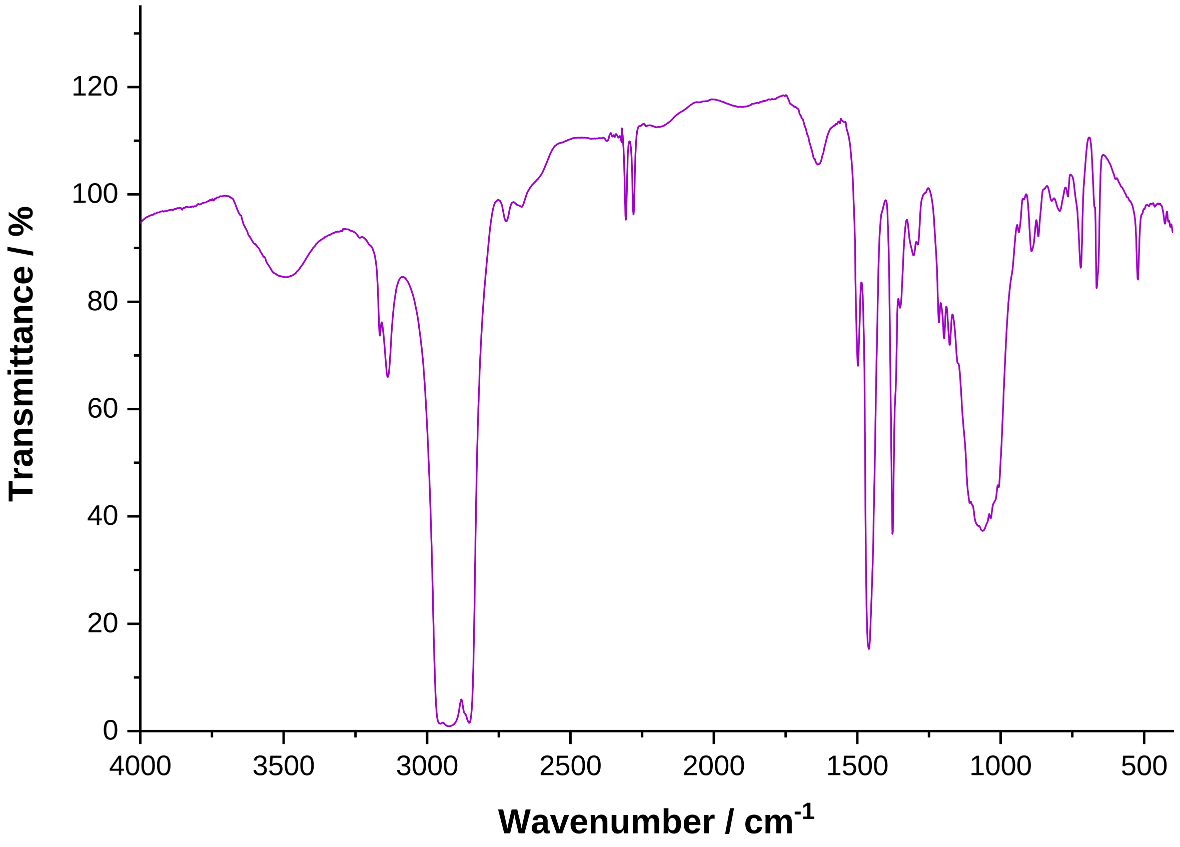


**Figure SI 10.** FT-IR (Nujol mull, KBr windows, 298 K) spectrum recorded for the homochiral coordination compound **1B**.

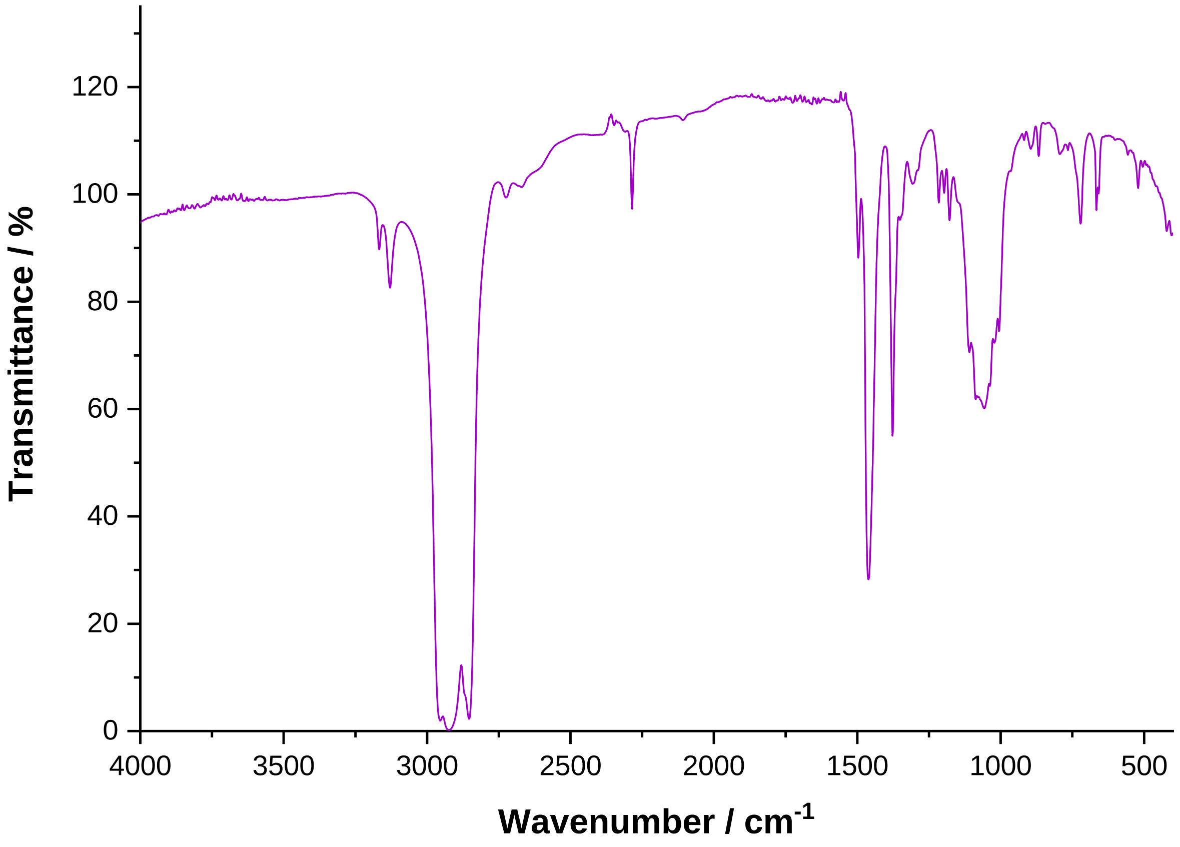


**Figure SI 11.** FT-IR (Nujol mull, KBr windows, 298 K) spectrum recorded for the homochiral coordination compound **2B**.

## Thermogravimetric analysis of the coordination compounds

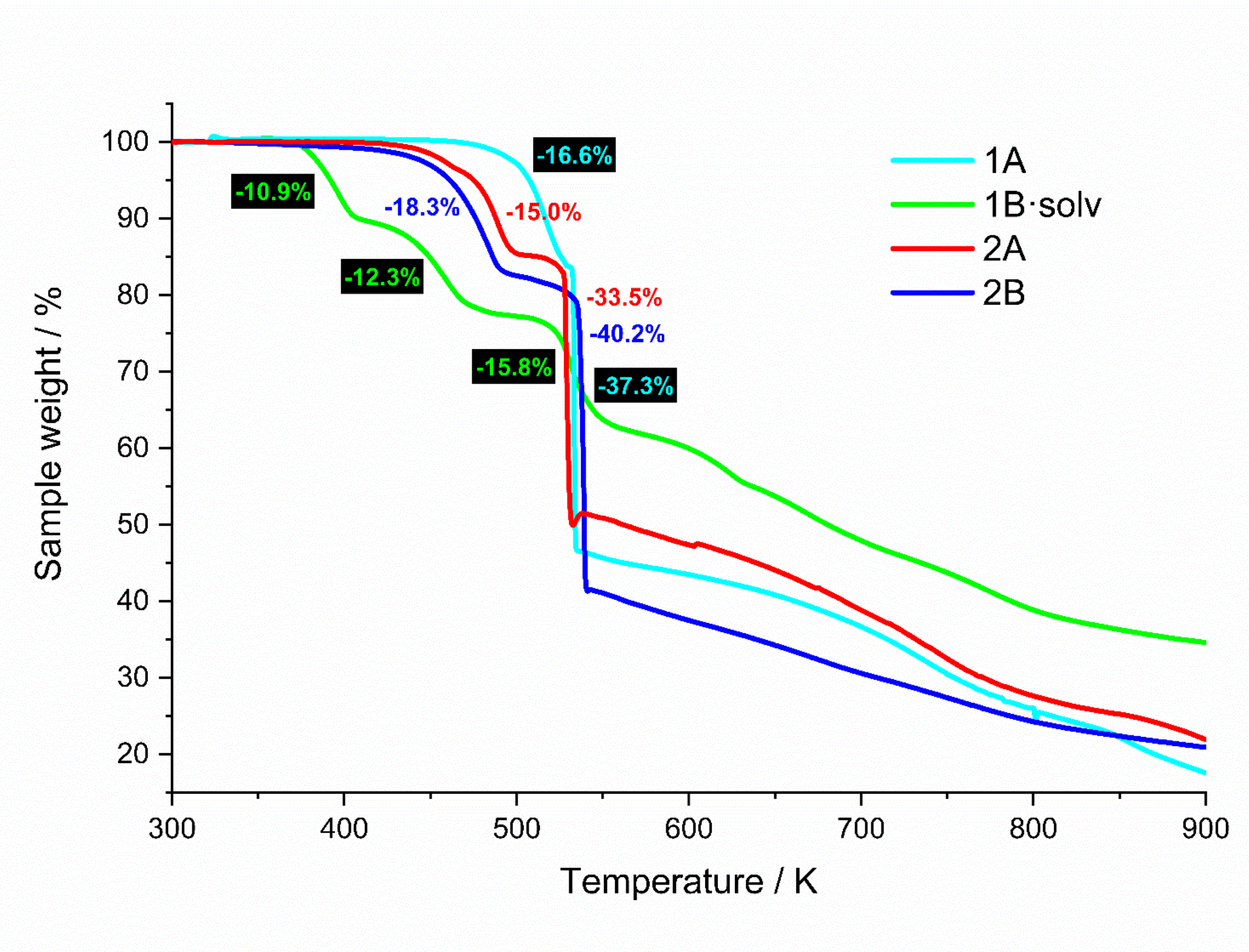


**Figure SI 12.** Results of the thermogravimetric analysis (10 K·min$^{-1}$, $N_2$ atmosphere) performed for the coordination compounds.

## DSC monitoring of the desolvation process of 1B·solv

In order to determine more precisely the lowest possible temperature, needed for the desolvation of **1B·solv**, we performed an experiment using DSC measurements for monitoring of the desolvation process. Namely, following sequence of cooling/heating of a sample was exploited:

1) <u>300 K → 120 K → 300 K (10 K·min$^{-1}$)</u>
2) 300 K → 350 K (20 K·min$^{-1}$) → 355 K (1 K·min$^{-1}$)
3) **355 K isotherm (5 min)** → 300 K (20 K·min$^{-1}$)
4) <u>300 K → 120 K → 300 K (10 K·min$^{-1}$)</u>
5) 300 K → 355 K (20 K·min$^{-1}$) → 360 K (1 K·min$^{-1}$)
6) **360 K isotherm (5 min)** → 300 K (20 K·min$^{-1}$)
7) <u>300 K → 120 K → 300 K (10 K·min$^{-1}$)</u>
8) 300 K → 365 K (20 K·min$^{-1}$) → 370 K (1 K·min$^{-1}$)
9) **370 K isotherm (5 min)** → 300 K (20 K·min$^{-1}$)
10) <u>300 K → 120 K → 300 K (10 K·min$^{-1}$)</u>
11) 300 K → 370 K (20 K·min$^{-1}$) → 375 K (1 K·min$^{-1}$)
12) **375 K isotherm (5 min)** → 300 K (20 K·min$^{-1}$)
13) <u>300 K → 120 K → 300 K (10 K·min$^{-1}$)</u>
14) 300 K → 375 K (20 K·min$^{-1}$) → 380 K (1 K·min$^{-1}$)
15) **380 K isotherm (5 min)** → 300 K (20 K·min$^{-1}$)
16) <u>300 K → 120 K → 300 K (10 K·min$^{-1}$)</u>

Repeated measurement cycle 300 K→120 K→300 K was used to monitor the disappearing/appearing of peaks, which correspond to **1B·solv** (at *ca* 145 K) / **1B** (at *ca* 225 K), respectively. Based on these results (presented in **Fig. SI 13**) we found out that at 355 K the desolvation process of **1B·solv** already starts. However, only above 370 K the process completes, i.e. the curves obtained for the sample after 375 K and 380 K isothermic heating are identical.

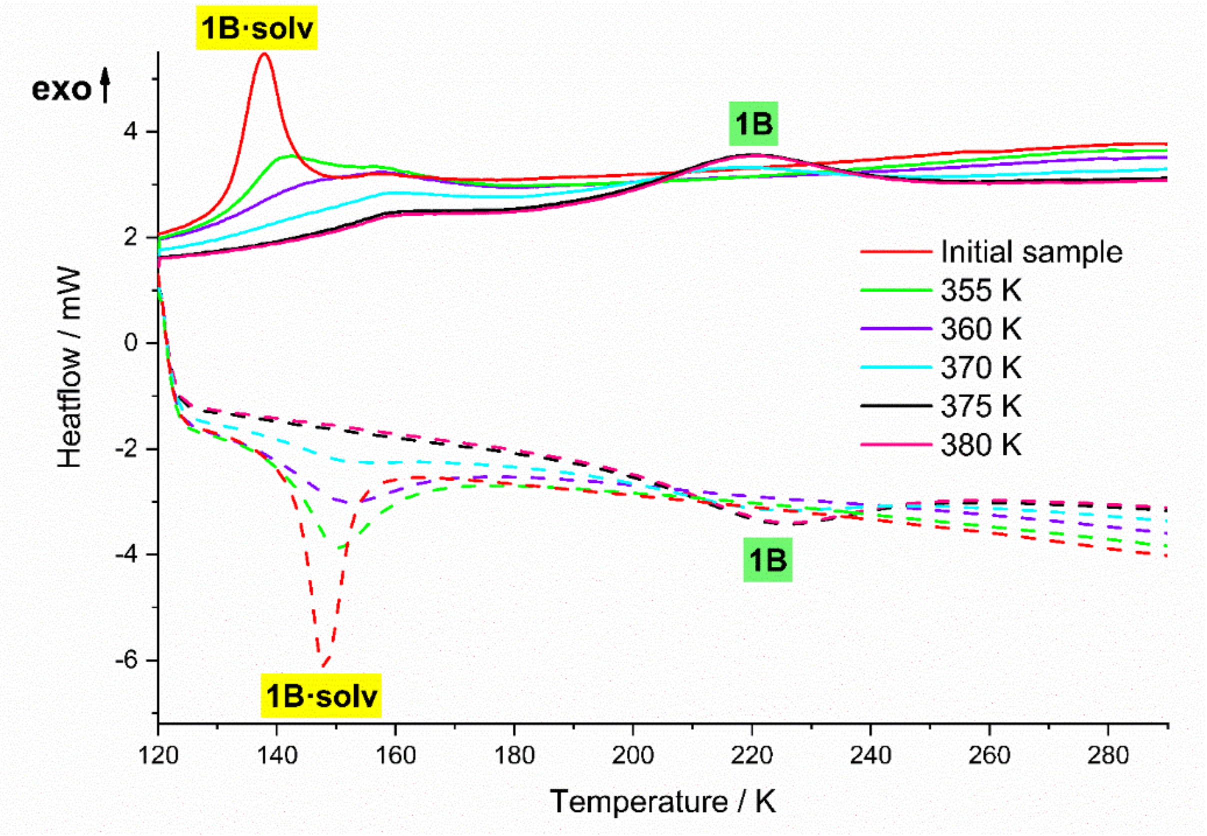


**Figure SI 13.** Monitoring of the desolvation process of **1B·solv** using DSC. Temperature values for different curves denote the curve path for a sample obtained after corresponding isothermic heating. Solid lines denote cooling mode, dashed lines – heating mode. Baseline was not corrected. A knee of a curve repeating in a cooling mode at *ca.* 165 K is a measurement artifact.

## Crystal data and experimental and refinement details

**Table SI 1.** Crystal data and experimental and refinement details for crystals of the ligands (racemic **A** and heterochiral **B**).

| **CCDC no.** | **2364829** | **2364830** |
|---|---|---|
| **Compound** | (*rac*)-1,2-di(1-Tz)Cp<br>**A** | (*1R,2R*)-1,2-di(1-Tz)Cp<br>**B** |
| **Molecular formula** | $C_7H_{10}N_8$ | $C_7H_{10}N_8$ |
| **$M_w$ / g·mol$^{-1}$** | 206.23 | 206.23 |
| **T / K** | 100(2) | 100(2) |
| **Crystal system** | monoclinic | orthorombic |
| **Space group** | $P2_1/n$ | $P2_12_12_1$ |
| **a / Å** | 9.970(1) | 5.346(1) |
| **b / Å** | 6.819(1) | 12.529(2) |
| **c / Å** | 14.101(2) | 28.033(6) |
| **α / °** | 90 | 90 |
| **β / °** | 103.56(1) | 90 |
| **γ / °** | 90 | 90 |
| **Volume / Å$^3$** | 931.9(2) | 1877.7(6) |
| **Z'** | 1 | 2 |
| **Z** | 4 | 8 |
| **$\rho_{calc}$ / g·cm$^{-3}$** | 1.470 | 1.459 |
| **μ / mm$^{-1}$** | 0.867 | 0.861 |
| **F(000)** | 432.0 | 864.0 |
| **Crystal size / mm$^3$** | 0.434 × 0.173 × 0.114 | 0.426 × 0.055 × 0.054 |
| **Radiation** | Cu Kα (λ = 1.54184 Å) | Cu Kα (λ = 1.54184 Å) |
| **2Θ range for data collection / °** | 9.864 to 149.692 | 6.306 to 151.18 |
| **Index ranges** | $-12 \le h \le 12$<br>$-8 \le k \le 7$<br>$-17 \le l \le 17$ | $-6 \le h \le 5$<br>$-15 \le k \le 15$<br>$-35 \le l \le 34$ |
| **Reflections collected** | 11820 | 24316 |
| **Independent reflections [$R_{int}$ / $R_{sigma}$]** | 1908<br>[$R_{int}$ = 0.0149 /<br>$R_{sigma}$ = 0.0089] | 3849<br>[$R_{int}$ = 0.0199 /<br>$R_{sigma}$ = 0.0121] |
| **Data/restraints/parameters** | 1908/5/143 | 3849/2/276 |
| **Goodness-of-fit on F$^2$** | 1.087 | 1.031 |
| **Final R indexes [I≥2σ (I)]** | $R_1$ = 0.0383<br>$wR_2$ = 0.0939 | $R_1$ = 0.0235<br>$wR_2$ = 0.0614 |
| **Final R indexes [all data]** | $R_1$ = 0.0388<br>$wR_2$ = 0.0942 | $R_1$ = 0.0240<br>$wR_2$ = 0.0617 |
| **Largest diff. peak/hole / e·Å$^{-3}$** | 0.27/-0.27 | 0.20/-0.13 |
| **Flack parameter** | N.A. | 0.00(6) |

**Table SI 2.** Crystal data and experimental and refinement details for crystals of the coordination compounds.

| **CCDC no.** | **2364831** | **2364832** | **2364833** | **2364834** | **2364835** | **2364836** | **2364837** | **2364838** |
|---|---|---|---|---|---|---|---|---|
| **Compound** | **1A** | | **1B·solv** | | **2A** | | **2B** | |
| **Molecular formula** | $[Fe(C_7H_{10}N_8)_2(CH_3CN)_2](BF_4)_2$ | | $[Fe(C_7H_{10}N_8)_2(CH_3CN)_2](BF_4)_2 \cdot 2CH_3CN$ | | $[Fe(C_7H_{10}N_8)_2(C_2H_5CN)_2](BF_4)_2$ | | $[Fe(C_7H_{10}N_8)_2(C_2H_5CN)_2](BF_4)_2$ | |
| **Empirical formula** | $C_{18}H_{26}B_2F_8FeN_{18}$ | | $C_{22}H_{32}B_2F_8FeN_{20}$ | | $C_{20}H_{30}B_2F_8FeN_{18}$ | | $C_{20}H_{30}B_2F_8FeN_{18}$ | |
| **$M_w$ / g·mol$^{-1}$** | 724.04 | | 806.15 | | 752.09 | | 752.09 | |
| **T / K** | **80** | **250** | **80** | **250** | **80** | **250** | **80** | **310** |
| **Spin state** | **HS** | | **LS** | **HS** | **HS** | | **LS** | **HS** |
| **Crystal system** | monoclinic | | triclinic | | monoclinic | | orthorombic | |
| **Space group** | $P2_1/n$ | | $P1$ | | $P2_1/n$ | | $P2_12_12_1$ | |
| **a / Å** | 8.887(2) | 9.066(3) | 9.424(3) | 9.747(3) | 9.293(3) | 9.381(4) | 10.085(4) | 10.476(2) |
| **b / Å** | 18.947(3) | 18.978(6) | 11.358(4) | 11.505(3) | 18.950(5) | 19.308(8) | 34.512(13) | 34.969(8) |
| **c / Å** | 9.334(2) | 9.427(3) | 9.147(3) | 9.416(3) | 9.492(3) | 9.529(4) | 8.763(4) | 9.027(3) |
| **α / °** | 90 | 90 | 105.61(3) | 105.85(2) | 90 | 90 | 90 | 90 |
| **β / °** | 109.51(2) | 110.39(3) | 96.12(3) | 97.06(2) | 114.77(4) | 114.60(2) | 90 | 90 |
| **γ / °** | 90 | 90 | 113.05(3) | 111.84(3) | 90 | 90 | 90 | 90 |
| **Volume / Å$^3$** | 1481.4(5) | 1520.3(9) | 842.5(5) | 912.6(5) | 1517.8(9) | 1569.3(12) | 3050(2) | 3307(2) |
| **Z** | 2 | 2 | 1 | 1 | 2 | 2 | 4 | 4 |
| **$\rho_{calc}$ / g·cm$^{-3}$** | 1.623 | 1.582 | 1.589 | 1.467 | 1.646 | 1.592 | 1.638 | 1.511 |
| **μ / mm$^{-1}$** | 0.606 | 0.591 | 0.544 | 0.502 | 4.875 | 4.715 | 4.852 | 4.467 |
| **F(000)** | 736.0 | 736.0 | 412.0 | 412.0 | 768.0 | 768.0 | 1536.0 | 1536.0 |
| **Crystal size / mm$^3$** | 0.24 × 0.18 × 0.11 | 0.24 × 0.18 × 0.11 | 0.43 × 0.10 × 0.08 | 0.43 × 0.10 × 0.08 | 0.08 × 0.06 × 0.04 | 0.11 × 0.06 × 0.04 | 0.45 × 0.04 × 0.04 | 0.3 × 0.03 × 0.02 |
| **Radiation** | Mo Kα (λ = 0.71073 Å) | | Mo Kα (λ = 0.71073 Å) | | Cu Kα (λ = 1.54184 Å) | | Cu Kα (λ = 1.54184 Å) | |
| **2Θ range for data collection / °** | 4.300 to 59.144 | 4.292 to 56.564 | 4.144 to 57.396 | 4.066 to 57.398 | 9.334 to 146.138 | 9.160 to 144.158 | 5.122 to 146.51 | 5.054 to 147.084 |
| **Index ranges** | -12 ≤ h ≤ 12<br>-26 ≤ k ≤ 26<br>-12 ≤ l ≤ 12 | -12 ≤ h ≤ 12<br>-25 ≤ k ≤ 25<br>-12 ≤ l ≤ 12 | -12 ≤ h ≤ 12<br>-15 ≤ k ≤ 15<br>-12 ≤ l ≤ 12 | -13 ≤ h ≤ 13<br>-15 ≤ k ≤ 15<br>-12 ≤ l ≤ 12 | -10 ≤ h ≤ 11<br>-23 ≤ k ≤ 20<br>-11 ≤ l ≤ 11 | -11 ≤ h ≤ 11<br>-23 ≤ k ≤ 23<br>-11 ≤ l ≤ 11 | -12 ≤ h ≤ 12<br>-42 ≤ k ≤ 40<br>-10 ≤ l ≤ 6 | -12 ≤ h ≤ 12<br>-39 ≤ k ≤ 43<br>-11 ≤ l ≤ 10 |
| **Reflections collected** | 30596 | 19909 | 19027 | 21268 | 9473 | 16703 | 14878 | 21539 |
| **Independent reflections [$R_{int}$/$R_{sigma}$]** | 4152<br>[$R_{int}$ = 0.0198/<br>$R_{sigma}$ = 0.0118] | 3922<br>[$R_{int}$ = 0.0168/<br>$R_{sigma}$ = 0.0132] | 8276<br>[$R_{int}$ = 0.0399/<br>$R_{sigma}$ = 0.0454] | 8882<br>[$R_{int}$ = 0.0286/<br>$R_{sigma}$ = 0.0431] | 2906<br>[$R_{int}$ = 0.0327/<br>$R_{sigma}$ = 0.0369] | 3072<br>[$R_{int}$ = 0.0739/<br>$R_{sigma}$ = 0.0574] | 5658<br>[$R_{int}$ = 0.0524/<br>$R_{sigma}$ = 0.0773] | 6210<br>[$R_{int}$ = 0.0402/<br>$R_{sigma}$ = 0.0462] |
| **Data/restraints/parameters** | 4152/2/219 | 3922/30/256 | 8276/3/482 | 8882/5/492 | 2906/12/277 | 3072/12/277 | 5658/15/463 | 6210/25/509 |
| **Goodness-of-fit on $F^2$** | 1.042 | 1.038 | 1.068 | 1.029 | 1.048 | 1.096 | 0.975 | 1.035 |
| **Final R indexes [I≥2σ (I)]** | $R_1$ = 0.0291<br>$wR_2$ = 0.0716 | $R_1$ = 0.0356<br>$wR_2$ = 0.0956 | $R_1$ = 0.0312<br>$wR_2$ = 0.0758 | $R_1$ = 0.0347<br>$wR_2$ = 0.0896 | $R_1$ = 0.0517<br>$wR_2$ = 0.1317 | $R_1$ = 0.0642<br>$wR_2$ = 0.1759 | $R_1$ = 0.0569<br>$wR_2$ = 0.1307 | $R_1$ = 0.0492<br>$wR_2$ = 0.1118 |
| **Final R indexes [all data]** | $R_1$ = 0.0322<br>$wR_2$ = 0.0730 | $R_1$ = 0.0428<br>$wR_2$ = 0.0995 | $R_1$ = 0.0329<br>$wR_2$ = 0.0765 | $R_1$ = 0.0381<br>$wR_2$ = 0.0919 | $R_1$ = 0.0641<br>$wR_2$ = 0.1401 | $R_1$ = 0.1035<br>$wR_2$ = 0.2020 | $R_1$ = 0.0893<br>$wR_2$ = 0.1465 | $R_1$ = 0.0764<br>$wR_2$ = 0.1331 |
| **Largest diff. peak/hole / e·Å$^{-3}$** | 0.51/-0.31 | 0.36/-0.22 | 0.51/-0.40 | 0.31/-0.23 | 0.50/-0.53 | 0.33/-0.42 | 0.41/-0.44 | 0.24/-0.18 |
| **Flack parameter** | N/A | | -0.005(6) | 0.012(6) | N/A | | -0.007(4) | -0.004(3) |

## Crystal data for 1B and experimental and refinement details

The desolvated sample **1B** was prepared as described in the main text (in the Experimental section). The careful microscope inspection of the macroscopic sample of **1B** showed that initial monocrystals severely cracked and mainly turned to polycrystalline powder, however, we were able to found a few fragments that preserved the morphology of the starting monocrystals besides numerous cracks. Diffraction pre-experiment conducted at 80 K showed that approximately half of the recorded reflections could be quite well indexed (obviously taking into consideration the overall very low quality of the observed diffraction pattern) allowing to perform standard data collection.

Nevertheless, the quite long exposure times led to the crystal degradation after about 4 hours that did not allow us to collect full data set. However, despite this fact and very low quality of the obtained data (**Table SI 3**), we were managed to determine parameters of the unit cell of **1B** and then solve and refine chemically reasonable tentative model of the crystal structure of the desolvated form. We refined this model mainly isotropically (only Fe atoms were refined anisotropically) due to the very limited quality of the diffraction data and used same constraints (*SAME*, *SADI* commands) to preserve chemically reasonable geometry of counterions and some ligand molecules. Values of the distances of the iron(II) – nitrogen atoms of the donor groups (which are equal or below 2.0 A) confirmed the LS state of **1B** at 80 K which is in an agreement with magnetic studies. Importantly, the obtained model confirmed the absence of the non-coordinated acetonitrile molecules in **1B**. Also, the irregular distribution of the anions around polymeric chain (which is characteristic feature for the chiral SCO-active systems that is discussed in the main text) is preserved in the structure of **1B**. Besides the presence of two crystallographically independent Fe(II) cations (and two crystallographically independent polymeric units, respectively) the irregular very similar distribution pattern is observed for both macrocations (**Fig. SI 14**).

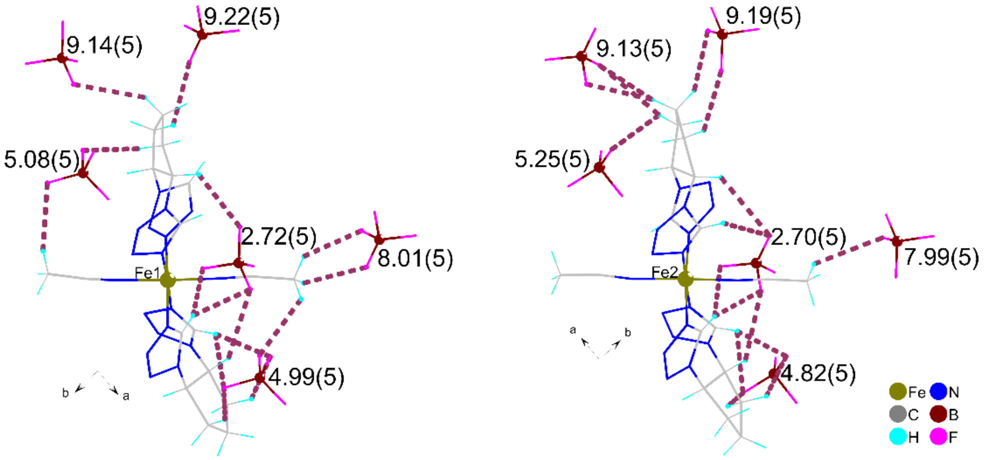


**Figure SI 14.** Distribution of anions around the polymeric units observed for the **1B** obtained *via* desolvation of **1B·solv**. Two crystallographically independent polymeric units are shown. Distances are given in Å and were measured as the distance at 80 K between the central boron atom of the corresponding anion and the "chain vector" defined by bridged iron(II) cations within shown chains. Only anions forming weak direct contacts (shown with a thick plum dashed line) with polymeric units are taken into consideration.

**Table SI 3.** Crystal data and experimental and refinement details for crystal of the **1B**.

| **Compound** | **1B** |
|---|---|
| **Molecular formula** | $[Fe(C_7H_{10}N_8)_2(CH_3CN)_2](BF_4)_2$ |
| **Empirical formula** | $C_{18}H_{26}B_2F_8FeN_{18}$ |
| **$M_w$ / g·mol$^{-1}$** | 724.04 |
| **T / K** | **80** |
| **Spin state** | **LS** |
| **Crystal system** | triclinic |
| **Space group** | *P1* |
| **a / Å** | 10.059(2) |
| **b / Å** | 16.775(3) |
| **c / Å** | 8.736(1) |
| **α / °** | 87.63(2) |
| **β / °** | 89.92(2) |
| **γ / °** | 87.41(2) |
| **Volume / Å$^3$** | 1471.3(4) |
| **Z** | 2 |
| **$\rho_{calc}$ / g·cm$^{-3}$** | 1.634 |
| **μ / mm$^{-1}$** | 5.004 |
| **F(000)** | 736.0 |
| **Crystal size / mm$^3$** | 0.19 × 0.07 × 0.04 |
| **Radiation** | Cu Kα (λ = 1.54184 Å) |
| **2Θ range for data collection / °** | 5.278 to 129.7 |
| **Index ranges** | -11 ≤ h ≤ 11, -18 ≤ k ≤ 17, -8 ≤ l ≤ 9 |
| **Reflections collected** | 6149 |
| **Independent reflections [$R_{int}$/$R_{sigma}$]** | 4642 [$R_{int}$ = 0.0740, $R_{sigma}$ = 0.0949] |
| **Data/restraints/parameters** | 4642/62/391 |
| **Goodness-of-fit on F$^2$** | 1.760 |
| **Final R indexes [I≥2σ (I)]** | $R_1$ = 0.2022, $wR_2$ = 0.4818 |
| **Final R indexes [all data]** | $R_1$ = 0.2930, $wR_2$ = 0.5332 |
| **Largest diff. peak/hole / e·Å$^{-3}$** | 1.31/-0.73 |
| **Flack parameter** | 0.07(3) |
| **Completeness** | 75.6% |

The refined model has not been deposited in the CCDC but we are attaching this structure as supplementary material. The checkCIF report of the final model contains 4 alerts of level A and 10 alerts of level B, which are mainly derived from uncomplete data set and very low data quality. It also has to be underlined that residual electron density does not derive from any unmodelled molecules in the crystal structure (it mainly focused near heavy iron cations and $BF_4^-$ anions which could be partly disordered).

Our discussion (presented on the next sections of the SI) concerning weak contacts in the structure of **1B** is quite speculative because of the high uncertainty of the bond lengths and angles values due to the restricted quality of the obtained structural model. However, we aim to underline the similarities between structures of **1B** and **2B**, which was discussed in the main text, and for this purpose the data quality is satisfactory.

## Geometric parameters of the weak interactions in the crystal structures of the coordination compounds

**Table SI 4.** Geometric parameters of the weak interactions in the crystal structure of **1A** at 80 K and 250 K. Disordered component appeared at 250 K is showed with *.

| D-H···A | H···A | D···A | D-H···A | H···A | D···A | D-H···A |
|---|---|---|---|---|---|---|
| Temperature | | 80 K | | | 250 K | |
| Spin state | | | HS | | | |
| **Polymeric chain – neighboring polymeric chain** | | | | | | |
| C61-H61B···N23[e] | 2.85 | 3.289(2) | 108.0 | 2.97 | 3.379(3) | 106.5 |
| **Supramolecular layer – neighboring supramolecular layer** | | | | | | |
| C5-H5BD···N13[b] | 2.59 | 3.509(2) | 154.2 | 2.65 | 3.590(3) | 160.1 |
| **Polymeric chain – non-coordinating $BF_4^-$** | | | | | | |
| C1-H1···F3[a] | 2.34 | 3.221(2) | 145.8 | 2.36 | 3.237(5) | 147.2 |
| C1-H1···F3A[a]* | | | | 2.63 | 3.472(12) | 142.7 |
| C2-H2···F2 | 2.48 | 3.436(2) | 159.2 | 2.38 | 3.319(4) | 158.7 |
| C3-H3AA···F1* | | | | 2.62 | 3.377(5) | 134.0 |
| C3-H3AB···F2[b] | 2.46 | 3.028(2) | 115.7 | 2.45 | 3.090(6) | 122.6 |
| C5-H5BC···F3[c] | 2.43 | 3.329(2) | 150.7 | | | |
| C5-H5AB···F4[c] | 2.54 | 3.248(2) | 128.5 | 2.71 | 3.403(7) | 127.9 |
| C15-H15...F2A[a]* | | | | 2.60 | 3.484(7) | 156.2 |
| C15-H15···F3[a] | 2.28 | 3.048(2) | 137.8 | 2.32 | 3.083(5) | 138.2 |
| C25-H25···F1[b] | 2.53 | 3.271(2) | 135.5 | 2.69 | 3.339(5) | 127.0 |
| C25-H25···F1[b]* | | | | 2.63 | 3.339(5) | 140.4 |
| C25-H25···F4A[b]* | | | | 2.53 | 2.983(10) | 110.2 |
| C61-H61A···F2[d] | 2.33 | 3.081(2) | 132.5 | | | |
| C61-H61C···F3 | 2.41 | 3.355(2) | 160.8 | | | |
| C61-H61C···F4 | 2.66 | 3.386(2) | 131.4 | | | |

[a] 1+x, y, z; [b] ½+x, -½-y, ½+z; [c] ½+x, -½-y, -½+z; [d] 1-x, -y, 1-z; [e] -1+x,+y,+z

**Table SI 5.** Geometric parameters of the weak interactions in the crystal structure of **2A** at 80 K and 250 K.

| **D-H···A** | **H···A** | **D···A** | **D-H···A** | **H···A** | **D···A** | **D-H···A** |
|---|---|---|---|---|---|---|
| **Temperature** | | **80 K** | | | **250 K** | |
| **Spin state** | | | **HS** | | | |
| **Polymeric chain – neighboring polymeric chain** | | | | | | |
| C60-H60B···N12[b] | 2.66 | 3.473(5) | 140.3 | 2.74 | 3.530(8) | 139.1 |
| C60-H60B···N13[b] | 2.74 | 3.473(5) | 132.0 | 2.75 | 3.503(8) | 135.0 |
| **Polymeric chain – non-coordinating $BF_4^-$** | | | | | | |
| C1-H1A..F3[a] | | | | 2.57 | 3.49(2) | 154.8 |
| C2-H2···F4 | 2.31 | 3.169(6) | 143.7 | 2.42 | 3.279(13) | 145.0 |
| C2-H2···F1A | 2.42 | 3.301(7) | 143.0 | 2.21 | 3.102(15) | 149.2 |
| C3-H3AB···F2[c] | 2.57 | 3.289(11) | 129.6 | | | |
| C3-H3AA···F4[c] | 2.48 | 3.221(6) | 131.5 | 2.58 | 3.343(17) | 134.7 |
| C3-H3BC···F1A[c] | 2.34 | 3.232(8) | 148.9 | 2.17 | 3.128(19) | 165.8 |
| C5A-H5AA···F2A[d] | 2.55 | 3.51(3) | 163.6 | 2.47 | 3.38(5) | 154.7 |
| C5A-H5AA···F3A[d] | 2.59 | 3.46(3) | 146.1 | | | |
| C15-H15···F2[a] | 2.28 | 2.883(10) | 120.7 | 2.33 | 2.921(14) | 120.5 |
| C15-H15···F4A[a] | 2.38 | 3.064(12) | 128.6 | 2.46 | 3.073(17) | 123.2 |
| C25-H25···F1A | | | | 2.64 | 3.313(19) | 129.4 |
| C25-H25···F2A | 2.56 | 3.477(15) | 163.2 | 2.61 | 3.51(3) | 161.0 |
| C25-H25···F4 | 2.17 | 2.937(5) | 137.1 | 2.26 | 3.044(13) | 140.7 |
| C60-H60C···F3 | 2.38 | 3.246(19) | 146.7 | 2.36 | 3.23(2) | 148.6 |
| C60-H60C···F2A | 2.41 | 3.215(15) | 138.8 | 2.44 | 3.28(3) | 144.8 |

[a] 1+x, y, z; [b] -1-x, -y, -1-z; [c] ½+x, 1/2-y, ½+z; [d] ½+x, -½-y, -½+z

**Table SI 6.** Geometric parameters of the weak interactions in the crystal structure of **1B·solv** at 80 K and 250 K.

| **D-H···A** | **<H···A>** | **<D···A>** | **<D-H···A** | **<H···A>** | **<D···A>** | **<D-H···A** |
|---|---|---|---|---|---|---|
| **Temperature** | | **80 K** | | | **250 K** | |
| **Spin state** | | **LS** | | | **HS** | |
| **Polymeric chain – neighboring polymeric chain** | | | | | | |
| C61-H61B···N13[i] | 2.69 | 3.188(4) | 111.9 | | | |
| C71-H71B···N43[c] | 2.61 | 3.182(4) | 117.1 | 2.59 | 123.6 | 123.6 |
| **Polymeric chain – non-coordinating $BF_4^-$** | | | | | | |
| C2-H2···F22 | 2.65 | 3.300(3) | 122.5 | | | |
| C2-H2···F23 | 2.41 | 3.387(3) | 163.9 | 2.45 | 3.403(4) | 162.6 |
| C3-H3A···F14 | 2.58 | 3.380(3) | 137.7 | | | |
| C4-H4A···F13[d] | 2.54 | 3.354(3) | 139.1 | | | |
| C5-H5A···F11[d] | 2.59 | 3.560(3) | 167.5 | | | |
| C15-H15···F13[d] | 2.33 | 3.169(3) | 147.1 | 2.45 | 3.275(5) | 146.0 |
| C15-H15···F23 | 2.54 | 3.079(3) | 116.2 | 116.8 | 3.120(4) | 116.8 |
| C25-H25···F14 | 2.42 | 3.295(3) | 153.3 | 2.56 | 3.408(5) | 150.0 |
| C31-H31···F14[f] | 2.64 | 3.427(3) | 135.8 | | | |
| C32-H32···F24 | 2.34 | 3.235(3) | 147.9 | 2.48 | 3.354(5) | 146.9 |
| C33-H33B···F11[g] | 2.61 | 3.507(3) | 150.5 | | | |
| C45-H45···F12[f] | 2.34 | 3.075(3) | 134.3 | 2.32 | 3.120(5) | 143.2 |
| C61-H61C···F13 | 2.66 | 3.201(4) | 115.4 | | | |
| C71-H71A···F24[c] | 2.55 | 3.278(3) | 131.0 | 2.53 | 3.302(6) | 137.0 |
| **Polymeric chain – non-coordinating $CH_3CN$** | | | | | | |
| C1-H1···N87[a] | 2.72 | 3.520(4) | 137.2 | 2.66 | 3.488(6) | 140.8 |
| C4-H4B···N97[e] | 2.67 | 3.390(4) | 129.6 | | | |
| C55-H55···N97[h] | 2.50 | 3.354(4) | 149.6 | 2.65 | 3.449(9) | 143.4 |
| C61-H61B···N97[i] | 2.67 | 3.430(5) | 134.3 | | | |
| C71-H71B···N97[b] | 2.75 | 3.611(4) | 147.1 | | | |
| **$BF_4$ – non-coordinating $CH_3CN$** | | | | | | |
| C89-H89A···F13[f] | 2.62 | 3.405(4) | 137.6 | | | |
| C89-H89A···F23[j] | 2.40 | 3.137(4) | 131.6 | 2.52 | 3.179(7) | 125.4 |
| C99-H99B···F12[f] | 2.49 | 3.456(4) | 170.9 | 2.61 | 3.493(9) | 151.9 |
| C99-H99C···F24[c] | 2.64 | 3.192(4) | 116.2 | | | |

[a] 1+x, 1+y, 1+z; [b] x, y, -1+z; [c] 1+x, y, z; [d] x, y, 1+z; [e] x, 1+y, z;
[f] x, -1+y, z; [g] -1+x, -1+y, z; [h] -1+x, y, z; [i] -1+x, y, -1+z; [j] x, -1+y, -1+z

**Table SI 7.** Geometric parameters of the weak interactions in the crystal structure of **2B** at 80 K and 250 K. Disordered component appeared at 310 K is showed with *.

| **D-H···A** | **<H···A>** | **<D···A>** | **<D-H···A** | **<H···A>** | **<D···A>** | **<D-H···A** |
|---|---|---|---|---|---|---|
| **Temperature** | | **80 K** | | | **310 K** | |
| **Spin state** | | **LS** | | | **HS** | |
| **Polymeric chain – neighboring polymeric chain** | | | | | | |
| C1-H1···N12[d] | 2.59 | 3.454(9) | 144.8 | 2.66 | 3.532(6) | 148.4 |
| C1-H1···N13[d] | 2.73 | 3.505(9) | 134.2 | 2.66 | 3.459(7) | 139.5 |
| C4-H4A···N52[e] | 2.53 | 3.513(11) | 169.6 | 2.61 | 3.579(10) | 174.9 |
| C70-H70B···N53[g] | 2.84 | 3.333(10) | 112.2 | 2.57 | 3.438(11) | 150.8 |
| C70-H70C···N22[g] | 2.68 | 3.458(10) | 136.7 | | | |
| **Supramolecular double layer – neighboring supramolecular double layer** | | | | | | |
| C61-H61B···N42[c] | 2.83 | 3.413(10) | 118.1 | 2.71 | 3.436(9) | 131.6 |
| **Polymeric chain – non-coordinating $BF_4^-$** | | | | | | |
| C2-H2···F23 | 2.45 | 3.158(9) | 127.3 | 2.50 | 3.243(9) | 132.6 |
| C3-H3B···F14 | 2.64 | 3.469(8) | 140.9 | | | |
| C5-H5B···F11[a] | 2.56 | 3.388(8) | 140.7 | | | |
| C15-H15···F23 | 2.12 | 3.052(9) | 168.1 | 2.18 | 3.100(8) | 170.0 |
| C25-H25···F12A* | | | | 2.57 | 3.47(4) | 161.5 |
| C25-H25···F14 | 2.30 | 3.217(9) | 162.0 | 2.25 | 3.166(14) | 166.7 |
| C33-H33A···F11A[j]* | | | | 2.54 | 3.487(19) | 164.1 |
| C33-H33B···F22[h] | 2.45 | 3.132(13) | 125.7 | 2.46 | 3.188(13) | 131.7 |
| C34-H34B···F23[f] | 2.50 | 3.390(10) | 150.1 | | | |
| C35-H35B···F24[h] | 2.43 | 3.325(10) | 149.9 | 2.56 | 3.388(10) | 143.3 |
| C60-H60B···F12A* | | | | 2.61 | 3.51(3) | 157.9 |
| C60-H60B···F14 | 2.37 | 3.281(9) | 153.6 | 2.50 | 3.364(15) | 149.8 |
| C71-H71B···F11[b] | 2.43 | 3.381(9) | 160.3 | 2.34 | 3.01(2) | 125.5 |

[a] x, y, 1+z; [b] 1+x, y, z; [c] -½ +x, ½-y, -z; [d] ½-x, 1-y, -½+z; [e] -1+x, y, z;
[f] 1+x, y, z; [g] 3/2-x, 1-y, -½+z; [h] ½+x, ½-y, -z+1; [i] x, y, -1+z; [j] 1+x, y, 1+z

**Table SI 8.** Geometric parameters of the weak interactions in the crystal structure of **1B** at 80 K..

| **D-H···A** | **<H···A>** | **<D···A>** | **<D-H···A** |
|---|---|---|---|
| **Temperature** | | **80 K** | |
| **Spin state** | | **LS** | |
| **Polymeric chain – neighboring polymeric chain** | | | |
| C34-H34A···N123[c] | 2.81 | 3.36(6) | 116.4 |
| C32-H32···N122[c] | 2.49 | 3.34(7) | 141.9 |
| C35-H35A···N122[c] | 2.81 | 3.45(8) | 123.3 |
| C114-H11E···N102[d] | 2.85 | 3.65(9) | 139.0 |
| C34-H34B···N12[c] | 2.42 | 3.39(6) | 166.4 |
| **Supramolecular double layer – neighboring supramolecular double layer** | | | |
| C4-H4A···N93[a] | 2.57 | 3.45(8) | 148.8 |
| C5-H5A···N92[a] | 2.84 | 3.65(9) | 139.4 |
| C84-H84B···N24[b] | 2.80 | 3.56(6) | 134.5 |
| **Polymeric chain – non-coordinating $BF_4^-$** | | | |
| C85-H85B ···F24[e] | 2.95 | 3.66(6) | 130.1 |
| C84-H84A ···F42[c] | 2.52 | 3.50(7) | 169.6 |
| C85-H85B ···F43[c] | 2.89 | 3.67(7) | 136.1 |
| C84-H84A ···F43[c] | 2.87 | 3.64(5) | 134.6 |
| C84-H84B ···F14[c] | 2.98 | 3.75(7) | 135.0 |
| C83-H83B ···F13[c] | 2.75 | 3.54(7) | 136.6 |
| C82-H82 ···F44 | 2.84 | 3.55(7) | 128.4 |
| C105-H105 ···F44 | 2.78 | 3.10(6) | 100.9 |
| C125-H125 ···F41 | 2.48 | 3.35(8) | 152.8 |
| C125-H125 ···F42 | 2.08 | 2.96(9) | 153.6 |
| C151-H15C ···F14 | 2.83 | 3.54(6) | 130.0 |
| C135-H135 ···F21[f] | 2.87 | 3.42(6) | 118.5 |
| C113-H11D ···F21[f] | 2.70 | 3.46(5) | 133.9 |
| C113-H11C ···F24[f] | 2.82 | 3.47(5) | 124.4 |
| C135-H135 ···F24[f] | 2.19 | 3.10(6) | 160.3 |
| C4-H4B ···F11[g] | 2.68 | 3.53(10) | 143.7 |
| C3-H3B ···F44[g] | 2.76 | 3.48(6) | 130.1 |
| C5-H5B ···F32[g] | 2.43 | 3.41(9) | 169.0 |
| C71-H71A ···F34[g] | 2.69 | 3.42(7) | 131.5 |
| C1-H1 ···F13[h] | 2.55 | 3.24(7) | 126.1 |
| C55-H55 ···F12_$9[i] | 2.49 | 3.40(6) | 161.3 |
| C55-H55 ···F11_$9[i] | 2.48 | 3.32(8) | 147.2 |
| C31-H31 ···F11_$9[i] | 2.74 | 3.57(8) | 140.8 |
| C61-H61A ···F43[j] | 2.36 | 3.08(6) | 129.8 |
| C61-H61C ···F44[j] | 2.57 | 3.26(7) | 127.7 |
| C61-H61B ···F32[k] | 2.63 | 3.27(7) | 122.7 |
| C45-H45 ···F33[c] | 2.36 | 3.29(5) | 164.5 |
| C33-H33A ···F34[k] | 2.48 | 3.29(6) | 138.8 |
| C45-H45 ···F34[c] | 2.71 | 3.35(6) | 125.1 |

[a] x, y-1, z-1; [b] x, y+1, z; [c] x+1, y, z; [d] x-1, y, z; [e] x+1, y, z+1; [f] x, y, z+1;
[g] x, y, z-1; [h] x, y-1, z; [i] x+1, y-1, z; [j] x+1, y-1, z-1; [k] x+1, y, z-1

## General description of the crystal structures of the ligands

Ligand **A** being a racemic mixture crystallizes in a monoclinic space group *$P2_1/c$* (see **Table SI 1** for the details). The asymmetric unit of ligand **A** consists (**Fig. SI 15**, **left**) of one independent ligand molecule lying on a general position. It should be noted that the same position in the unit cell is occupied by both optical isomers of the racemic ligand **A** that manifests as disordered optical centers (*C1*/*C1A* and *C2*/*C2A* atoms on **Fig. SI 15**, **left**, respectively) of the cyclopentane ring in the crystal structure with a site occupancy factors ratio 0.93/0.07. Homochiral ligand **B** crystallizes in orthorhombic space group *$P2_12_12_1$* (see **Table SI 1** for the details). The asymmetric unit of the ligand **B** consists (**Fig. SI 15**, **right**) of two crystallographically independent ligand molecules. The cyclopentane ring in one of these molecules adopts two slightly different conformations, which manifests as a disorder with a site occupancy factor ratio 0.51/0.49. **Fig. SI 16** represents crystal packing diagrams for ligands **A** and **B**.

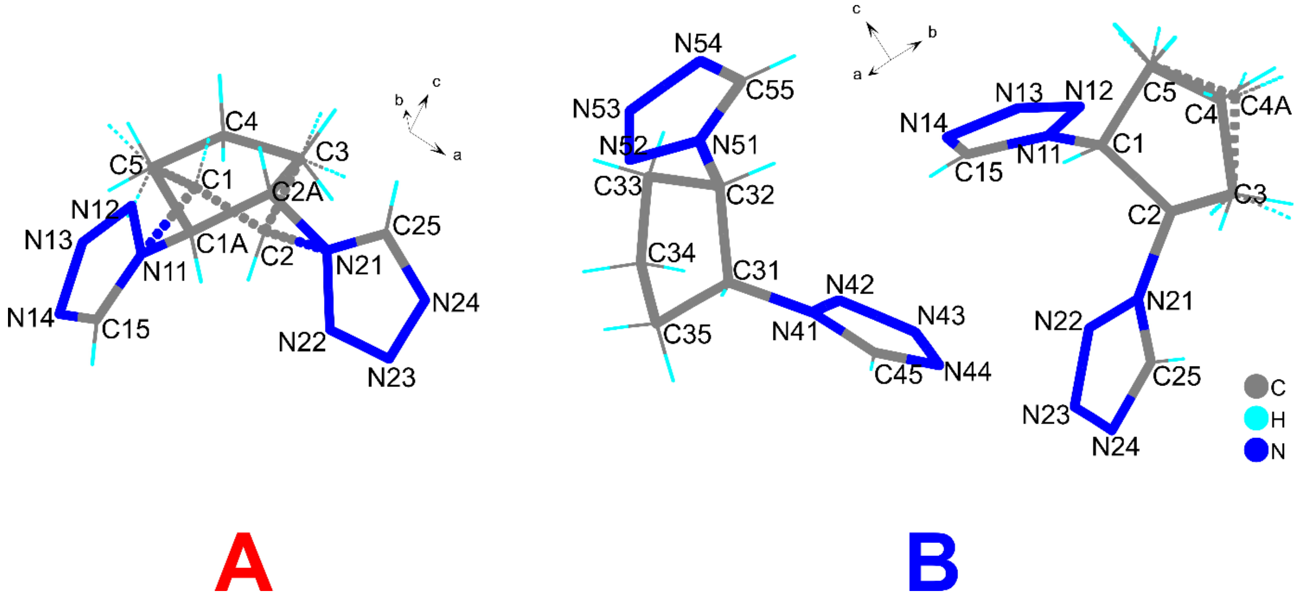


**Figure SI 15.** The asymmetric unit content of a unit cell of the crystal structure of the ligands with a non-hydrogen atom number scheme. The bonds involving atoms occupying minor disordered positions are represented with a dashed line. **A** represents structure of the racemic ligand, **B** – heterochiral ligand.

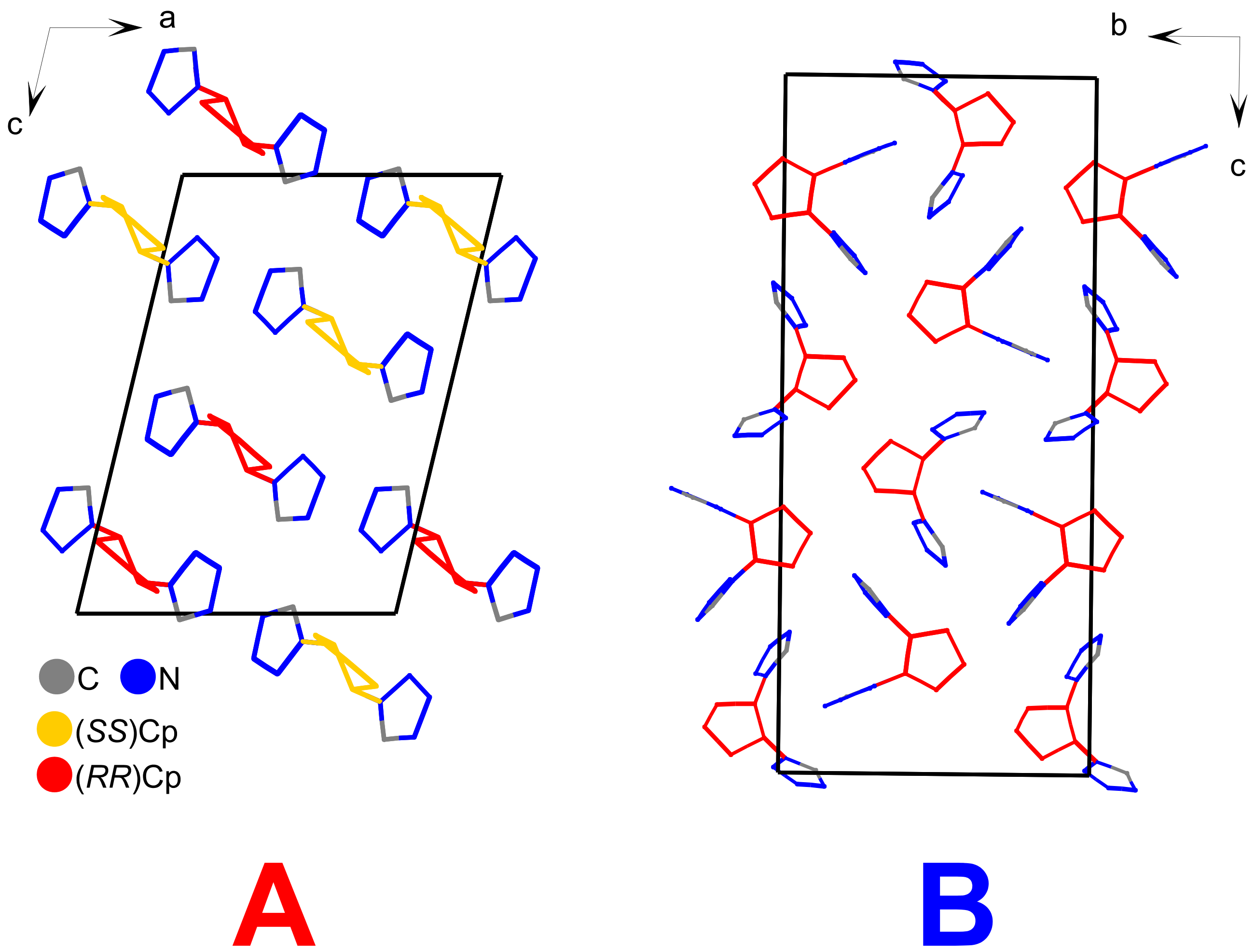


**Figure SI 16.** Crystal packing diagrams for the heterochiral (**A**) and homochiral (**B**) ligand. Hydrogen atoms and minor disorder components were omitted for clarity. Red and yellow colors denote the *trans*-1,2-disubstituted cyclopentane rings with different absolute configurations (*RR* and *SS*, respectively).

## General description of the crystal structures of the coordination compounds

The asymmetric unit of the heterochiral **1A** and **2A** (which are based on the ligand **A** being a racemic mixture) consists of iron(II) cation lying on the inversion center, one crystallographically independent ligand and nitrile (acetonitrile and propionitrile, respectively) molecule and one tetrafluoroborate counterion lying at the general position (**Fig. SI 17**, top). Anion is disordered in the crystal structure of **2A** over two general positions with site occupancy factor ratio 0.57/0.43 respectively. Interestingly, the disubstituted cyclopentane ring adopts slightly different conformations at both structures of **1A** and **2A**, which manifests as disordered atoms *C4*/*C4A* in **1A** and *C4*/*C4A*, *C5*/*C5A* in **2A** with site occupancy factor ratio 0.93/0.07 and 0.83/0.17 respectively. The asymmetric unit of the **1B·solv** and **2B** (which are based on the enantiomeric pure ligand **B**) consists of one crystallographically independent iron(II) cation, two crystallographically independent ligand molecules, two crystallographically independent nitrile (acetonitrile and propionitrile, respectively) molecules and two tetrafluoroborate counterions (**Fig. SI 17**, middle). **1B·solv**, being a solvate, contains also two crystallographically independent non-coordinated acetonitrile molecules. One of the $BF_4^-$ anions in the structure of **2B** is disordered over two positions with a site occupancy factor ratio 0.72/0.28. The asymmetric unit of the **1B** (**Fig. SI 17**, bottom) consists of two crystallographically independent iron(II) cations, four crystallographically independent ligand molecules, four crystallographically independent acetonitrile molecules and four tetrafluoroborate counterions

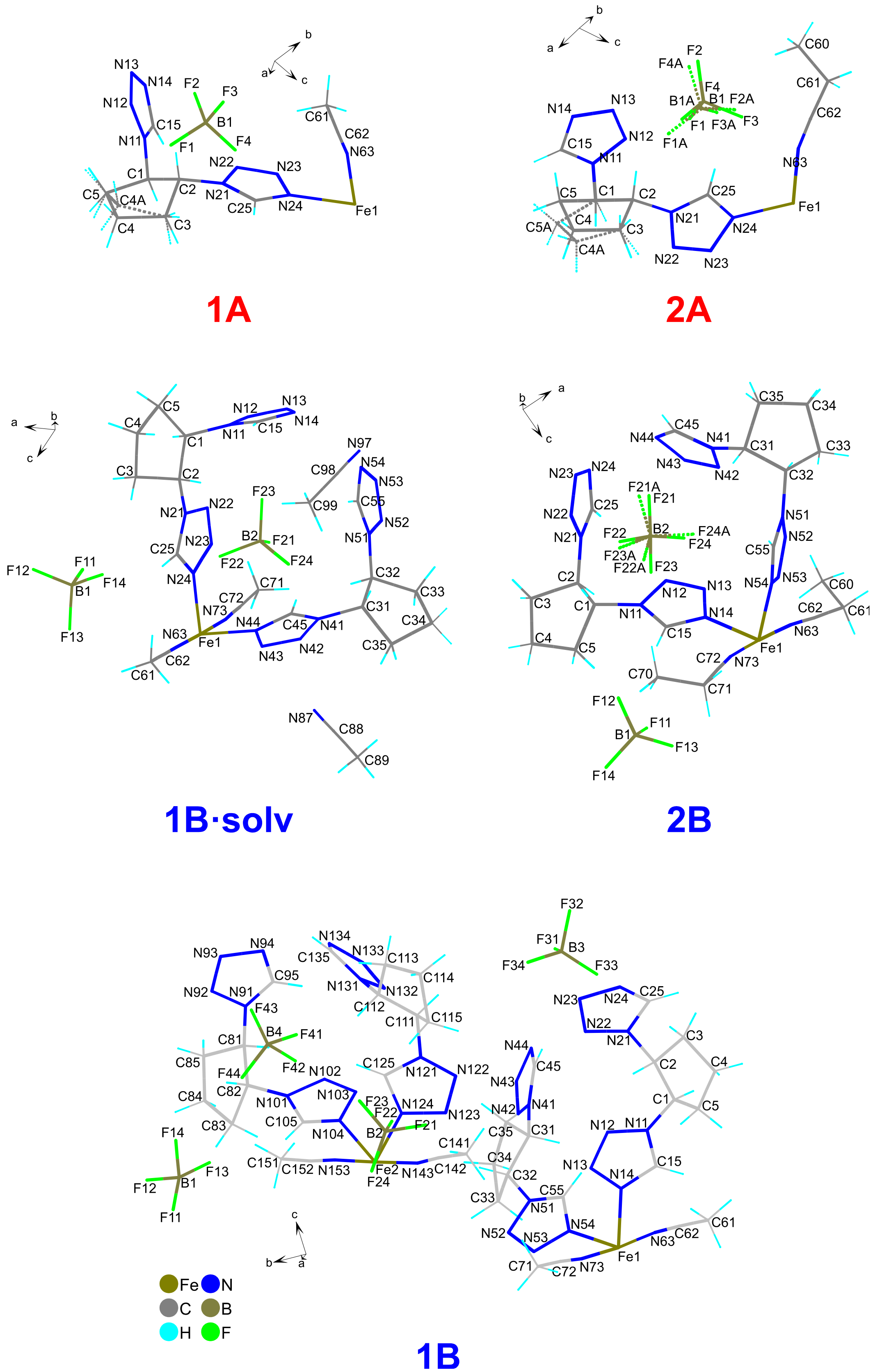


**Figure SI 17.** The asymmetric unit content of a unit cell of the crystal structures of the coordination compounds determined at 80 K with a non-hydrogen atom numbering scheme. The bonds involving atoms occupying minor disordered positions are represented with a dashed line.

## Detailed analysis of the weak interactions in the crystal structures of the coordination compounds

Analysis of the weak interactions presented in the crystal structures of the coordination compounds revealed a similar common pattern. Namely, in all compounds apart from **2B** and **1B** the neighboring polymeric units directly interact through weak contacts involving methyl groups of coordinated nitrile molecules (as donors of electron density) and tetrazole rings of neighboring polymeric chain (as acceptors of electron density). These contacts are propagating in *[100]* direction forming supramolecular layers that are parallel with *(010)* crystallographic plane (**Fig. SI 18-20**).

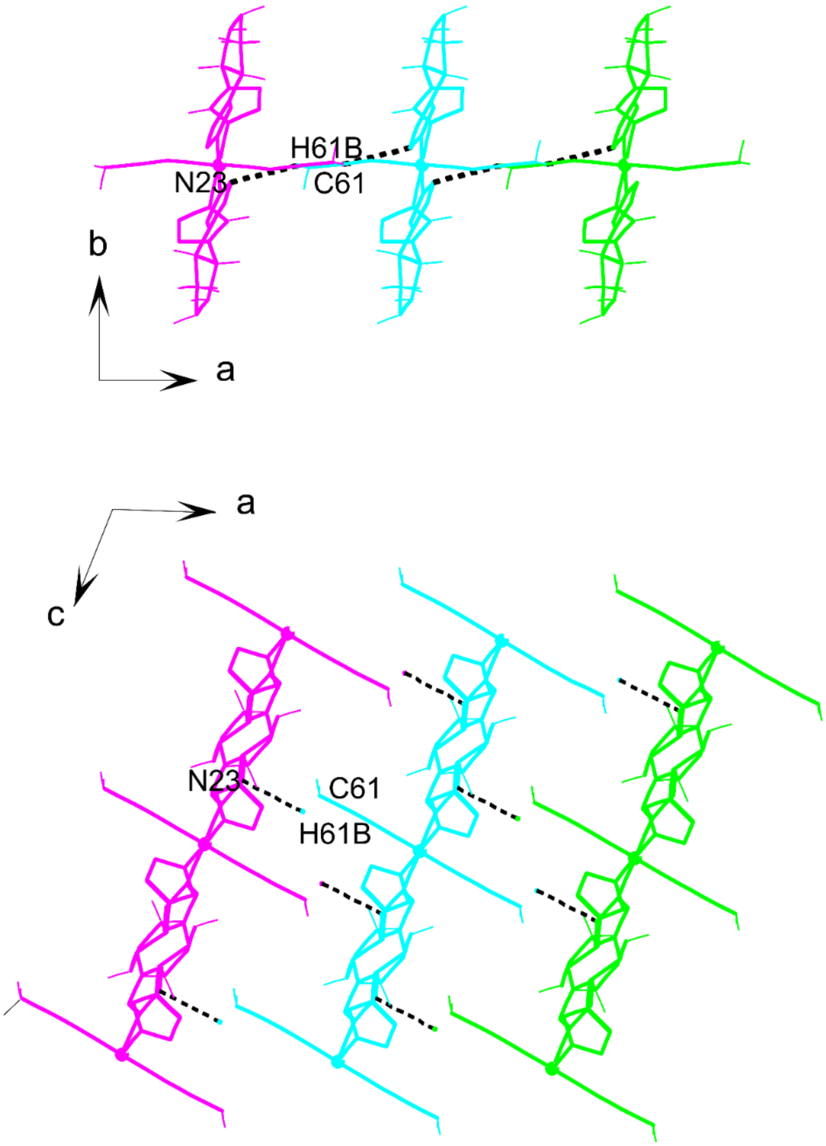


**Figure SI 18.** The supramolecular layer formed in **1A** formed through weak interactions (shown with a dashed black line) between polycationic chains. Each polymeric chain is shown with a different color (pink, turquoise, bright green). Fe(II) cations are represented with balls. Top: projection along polycationic unit (*[001]* direction). Bottom: projection perpendicular to formed layer (*(010)* plane).

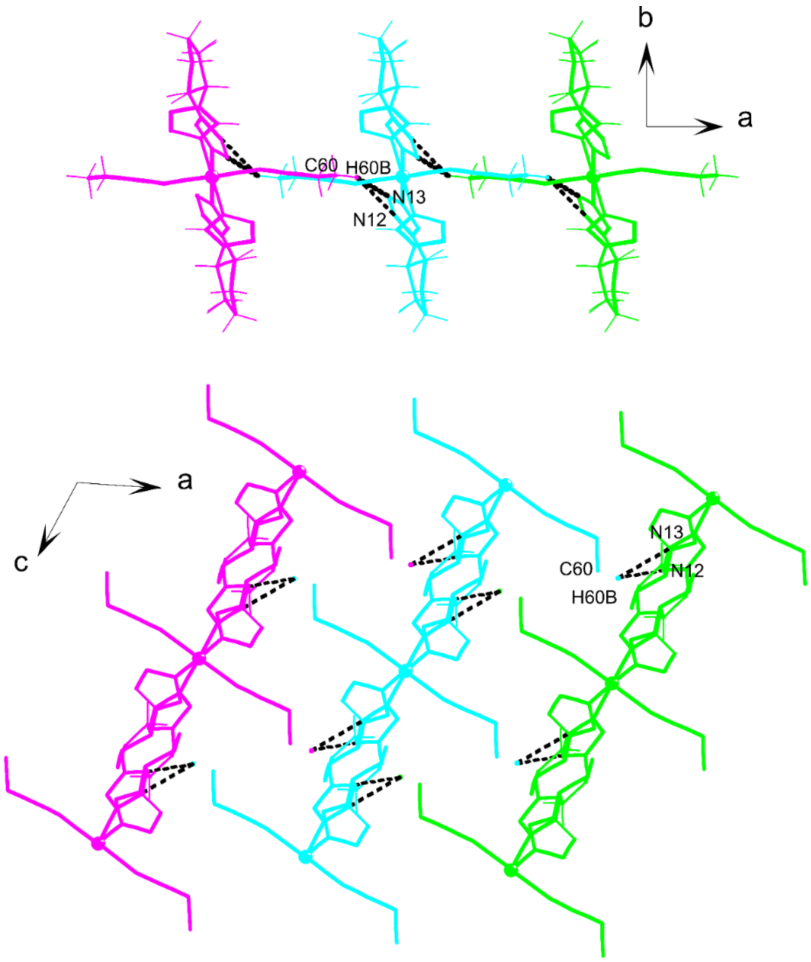


**Figure SI 19.** The supramolecular layer formed in **2A** formed through weak interactions (shown with dashed black line) between polycationic chains. Each polymeric chain is shown with different color (pink, turquoise, bright green). Fe(II) cations are represented with balls. Top: projection along polycationic unit (*[001]* direction). Bottom: projection perpendicular to formed layer (*(010)* plane).

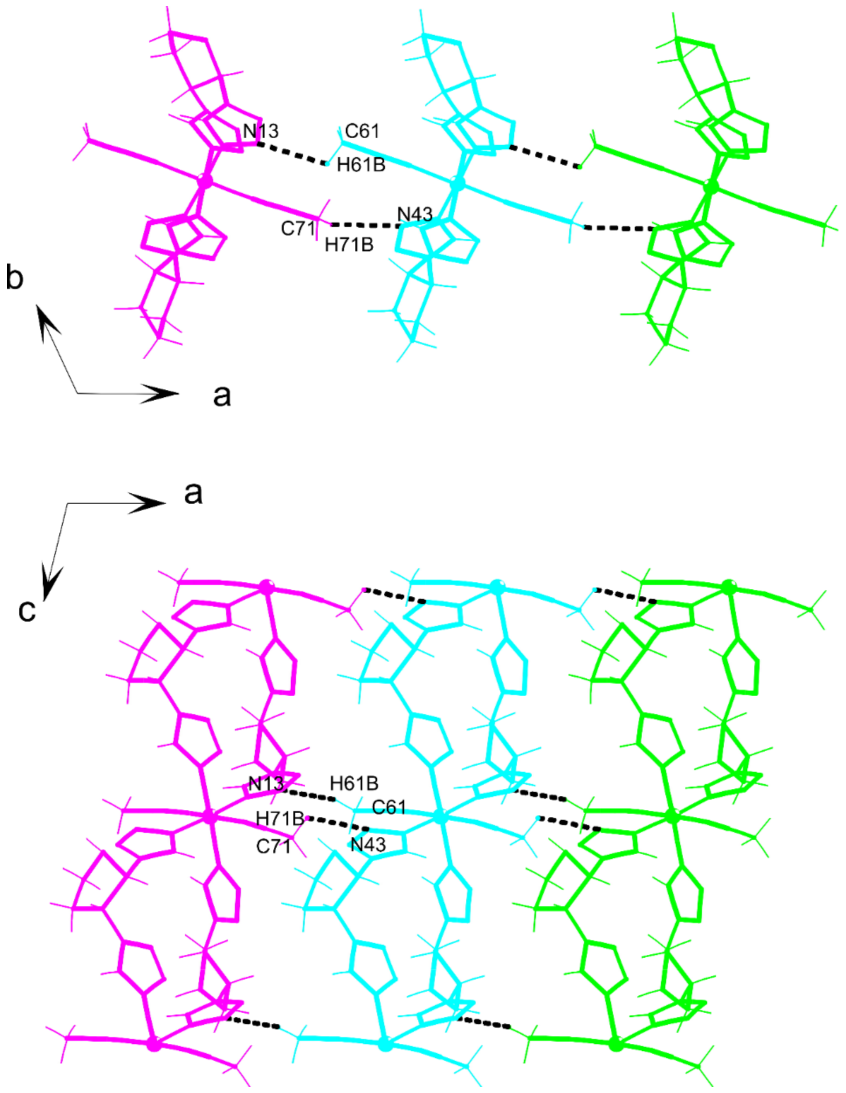


**Figure SI 20.** The supramolecular layer in **1B·solv** (80 K, LS state) formed through weak interactions (shown with a dashed black line) between polycationic chains. Each polymeric chain is shown with a different color (pink, turquoise, bright green). Fe(II) cations are represented with balls. Top: projection along polycationic unit (*[001]* direction). Bottom: projection perpendicular to formed layer (*(010)* plane).

A similar structural pattern is also observed in the structure of **2B**, however, the one is a little bit different and more complicated compared to the other three described complexes. Namely neighboring polymeric chains in **2B** interact not only through weak contacts involving methyl groups of one of the coordinated propionitrile molecules (*C70*) as donors of electron density and nitrogen atoms of tetrazole rings (*N12, N13, N52, N53, N22*) as acceptors (**Table SI 6**) but also exploiting atoms of cyclopentane rings (*C1, C4*). Contact *C4-H4A···N52* propagates in *[100]* direction and the other four contacts – in *[010]* direction forming a "double" supramolecular layer (**Fig. 21**) that is aligned with *(010)* plane. In contrast, the formation of the "mono" supramolecular layers was observed in **1A**, **2A** and **1B·solv** (**Fig. SI 18-20**).

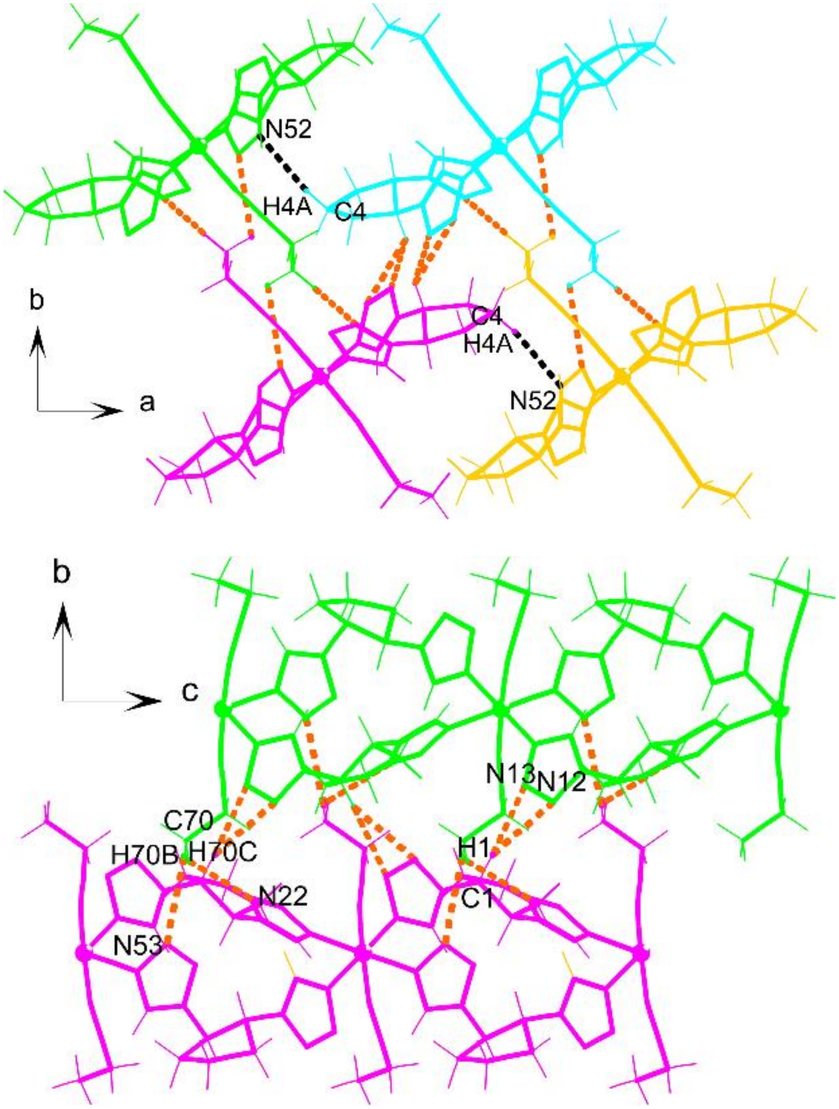


**Figure SI 21.** Forming of double supramolecular layers in **2B** at 80 K. Each polycationic unit is shown with common color (bright green, turquoise, pink, gold). Weak contacts propagating in *[100]* direction are shown with a black dashed line, the ones propagating in *[010]* direction – with an orange dashed line. Top: view along *[001]* direction; bottom: view along *[100]* direction. Iron(II) centers are represented with balls. Non-coordinated anions are omitted for clarity.

Formation of the pattern which is very close to the one observed in **2B** takes place in the structure of **1B** (**Fig. SI 22**), too.

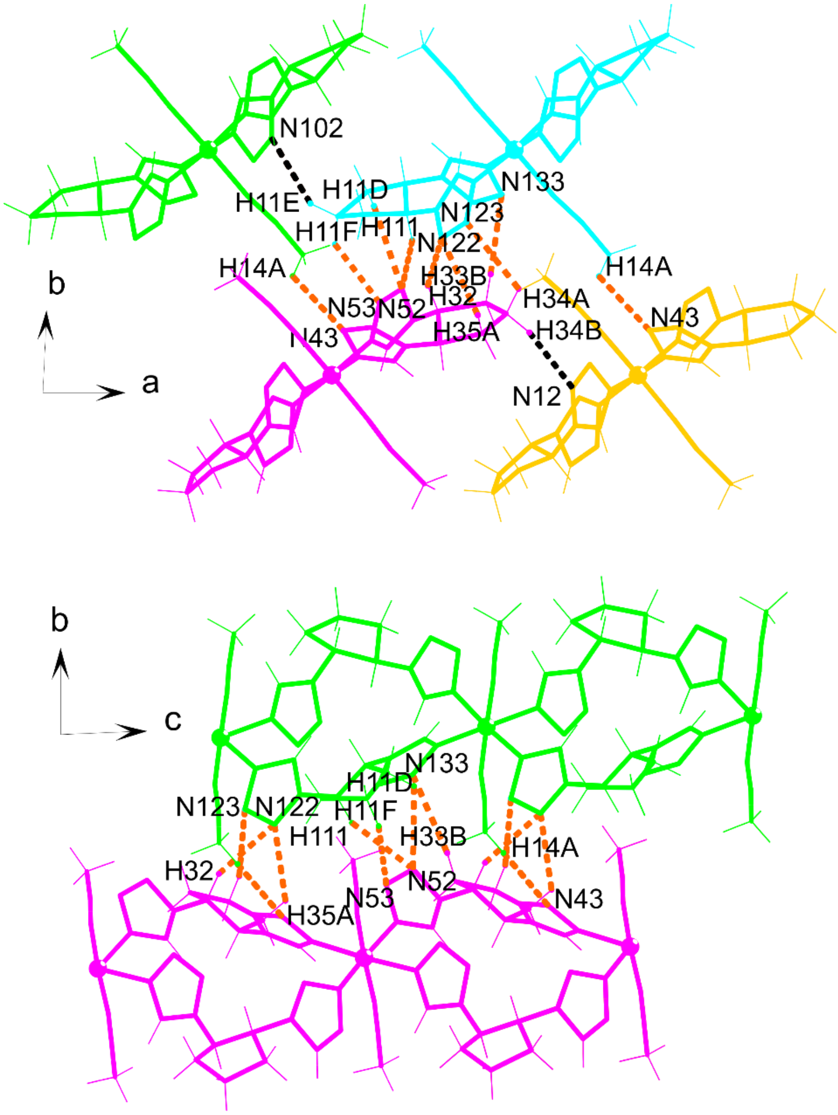


**Figure SI 22.** Forming of double supramolecular layers in **1B** at 80 K. Each polycationic unit is shown with common color (bright green, turquoise, pink, gold). Weak contacts propagating in *[100]* direction are shown with a black dashed line, the ones propagating in *[010]* direction – with an orange dashed line. Top: view along *[001]* direction; bottom: view along *[100]* direction. Iron(II) centers are represented with balls. Non-coordinated anions are omitted for clarity.

The described supramolecular layers directly interact in the **1A** (**Fig. SI 23**), **2B** (**Fig. SI 25**, **left**) and **1B** (**Fig. SI 26**) forming 3D network of weak contacts. Tetrafluoroborate anions interacting with supramolecular layers also contribute to the 3D network of the weak contacts in **1A** (**Fig. SI 24**) and **2B** (**Fig. SI 25**, **right**). Interestingly, one of the crystallographically independent anions in **2B** interacts only with one of the supramolecular layers (thus does not participate in the formation of a 3D network of the weak contacts), while the second one interacts with both layers (**Fig. SI 25**, **right**).

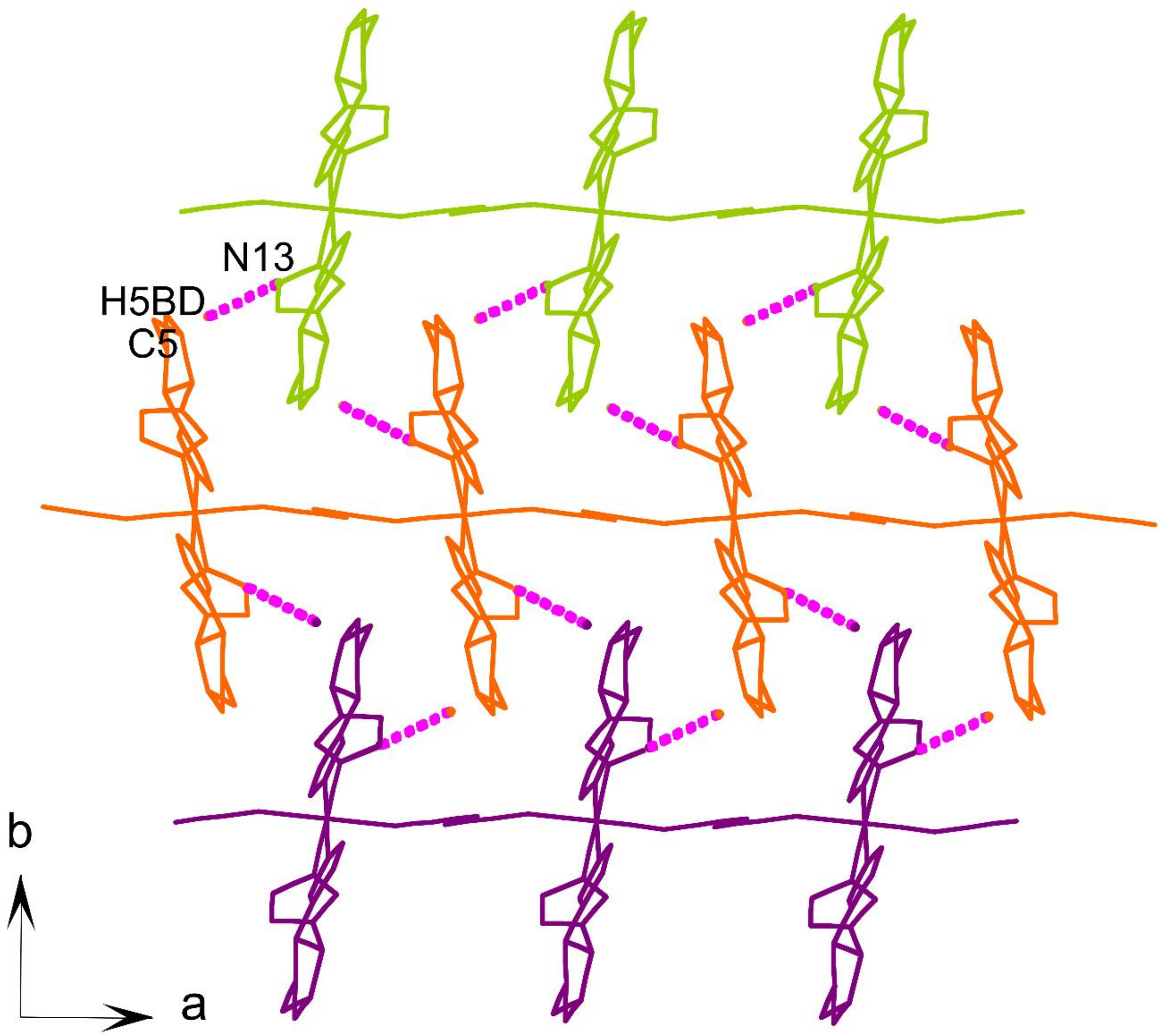


**Figure SI 23.** Direct interactions (shown with thick pink dashed line) of the supramolecular layers in **1A**. Polycationic chains forming a supramolecular layer are shown with common color (lime, orange, violet). The minor component of disordered cyclopentane ring, *C-H* bonds and anions are not shown for clarity. Direct interactions between chains (propagating in *[100]* direction) leading to the formation of a supramolecular layer are also omitted.

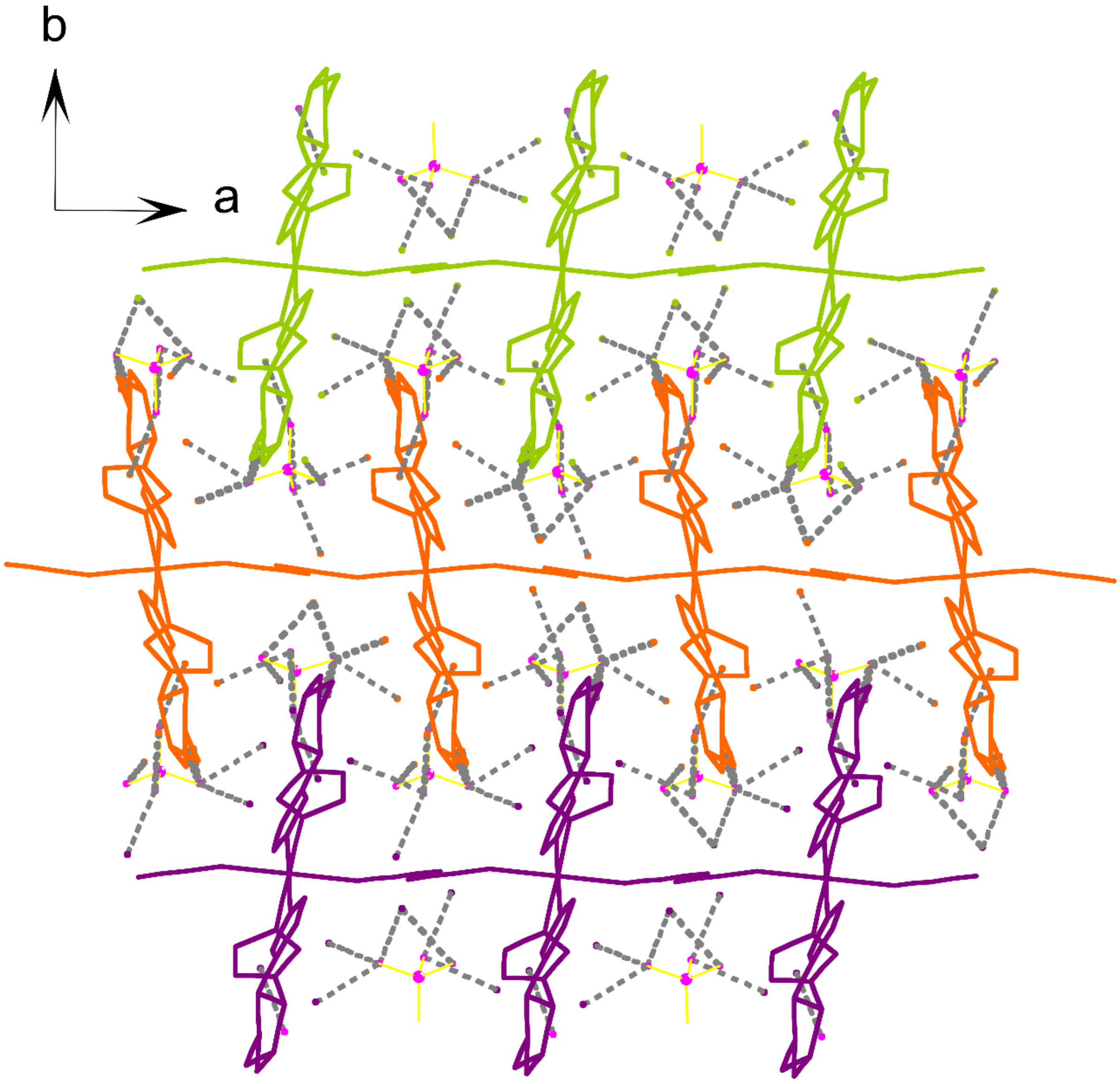


**Figure SI 24.** Contribution of anions to the 3D network of weak contacts (shown with grey dashed line) between supramolecular layers at 80 K in **1A**. Polycationic chains belonging to separate layers are shown with common color (lime, orange, violet). Fluorine and boron atoms are shown with pink color; *C-F* bonds are shown with yellow color. *C-H* bonds and direct interactions between layers and chains are not shown for clarity.

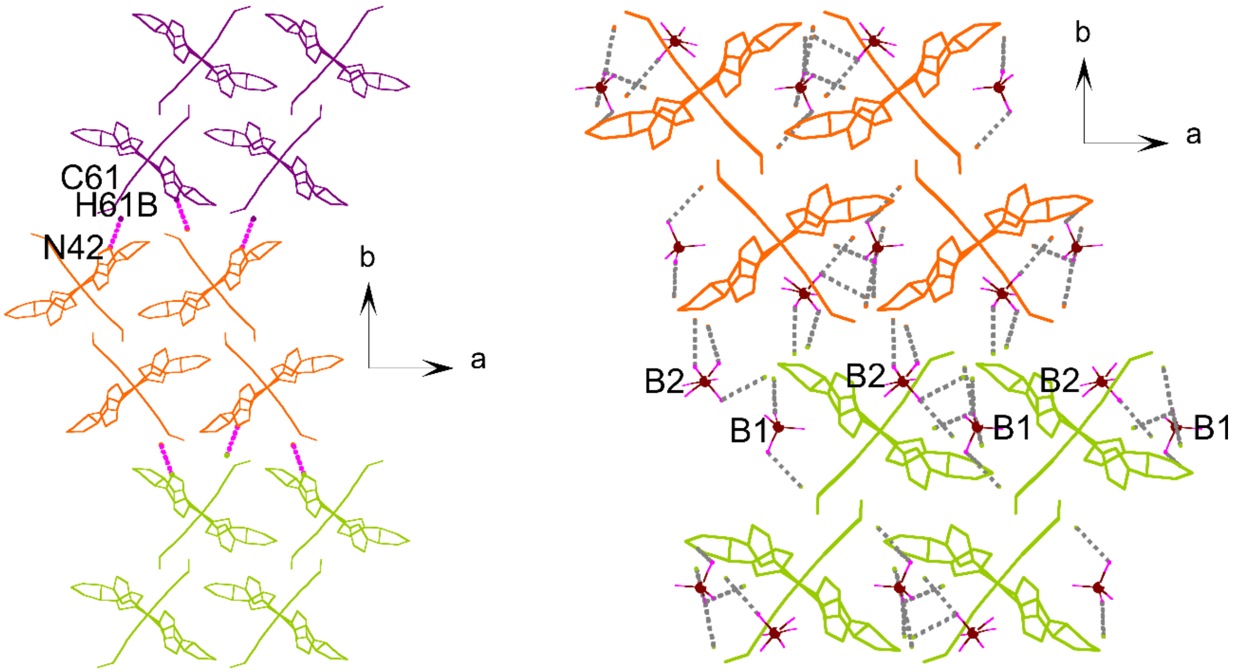


**Figure SI 25.** Interactions between double layers (chains forming the corresponding layer are shown with common color: violet, orange, lime) in **2B**. Left: direct interactions (shown with pink, thick dashed line) between layers, anions are omitted. Right: indirect interactions between layers (shown with grey thick dashed line) through anions, direct interactions between layers are omitted. Boron atoms are represented with dark red balls; *B-F* bonds are represented with a pink, thin line. *C-H* bonds are not shown for clarity. Interactions within double layers are omitted.

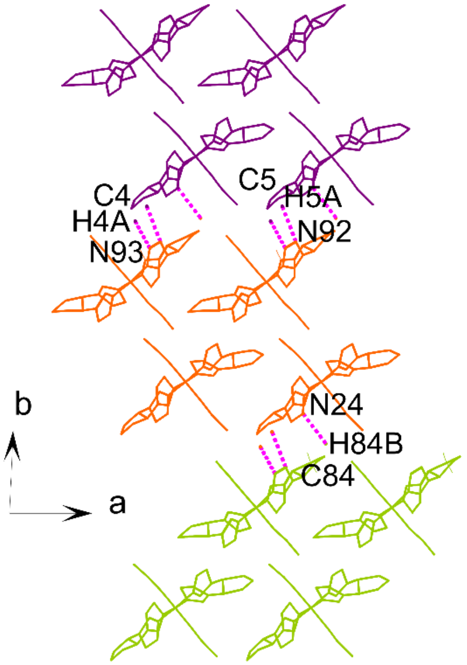


**Figure SI 26.** Interactions between double layers (chains forming the corresponding layer are shown with common color: violet, orange, lime) in **1B**. *C-H* bonds are not shown for clarity. Interactions within double layers are omitted.

In contrast to **1A** and **2B**, the supramolecular layers in the **2A** and **1B·solv** do not directly interact. The formation of the 3D network of the weak contacts in the corresponding crystal structures involves counterions (**Fig. SI 27-28**). Similar to **2B**, one of the crystallographically independent anions in **1B·solv** interacts only with one of the supramolecular layers, while the second counterion interacts with both layers (**Fig. SI 28**).

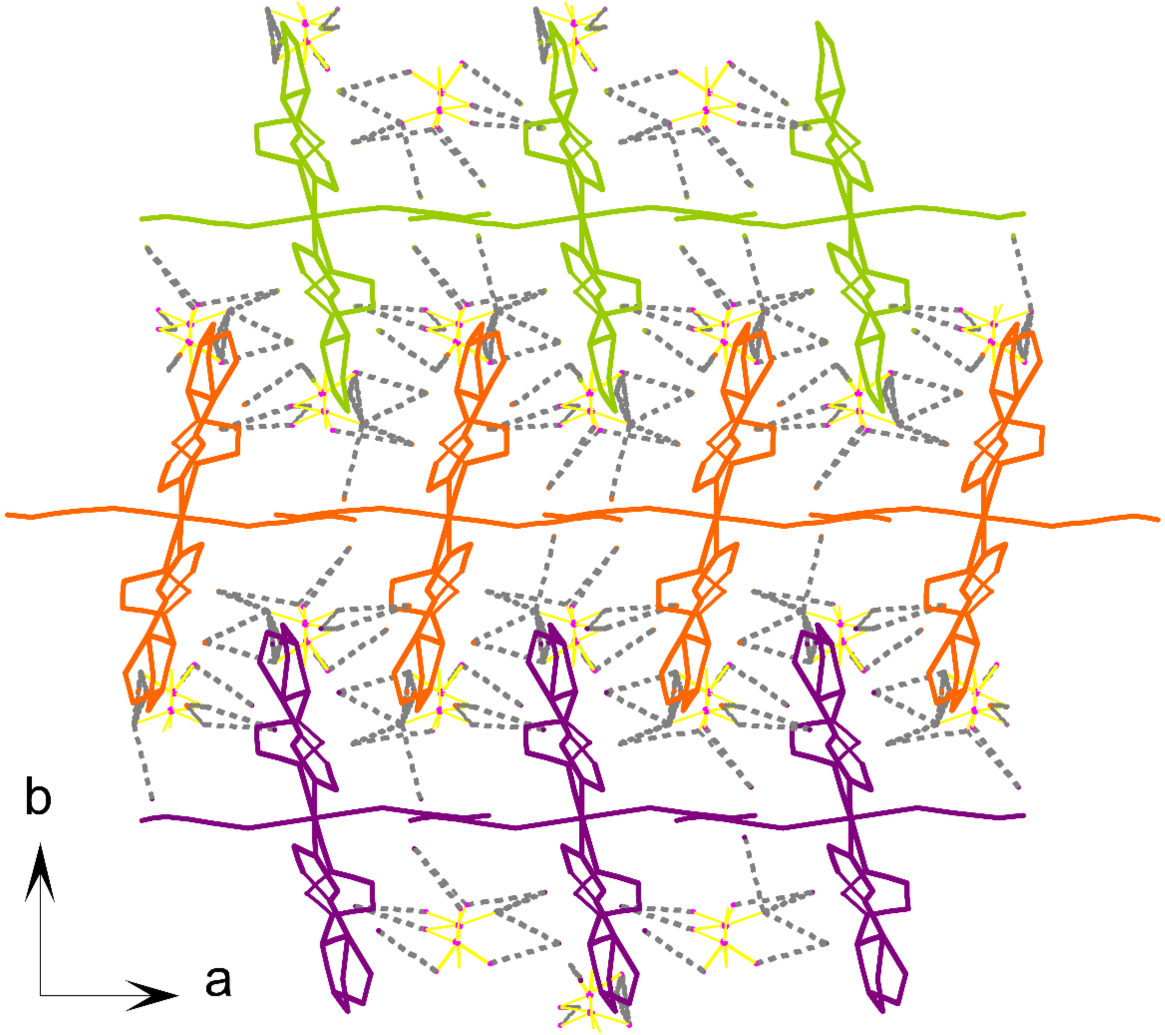


**Figure SI 27.** Forming of a 3D network of weak contacts (shown with grey dashed line) between supramolecular layers through interactions with $BF_4^-$ anions at 80 K in **2A**. Polycationic chains belonging to separate layers are shown with common color (lime, orange, violet). Fluorine and boron atoms are shown with pink color; *C-F* bonds are shown with yellow color. *C-H* bonds are not shown for clarity. Direct weak contacts between chains are omitted for clarity.

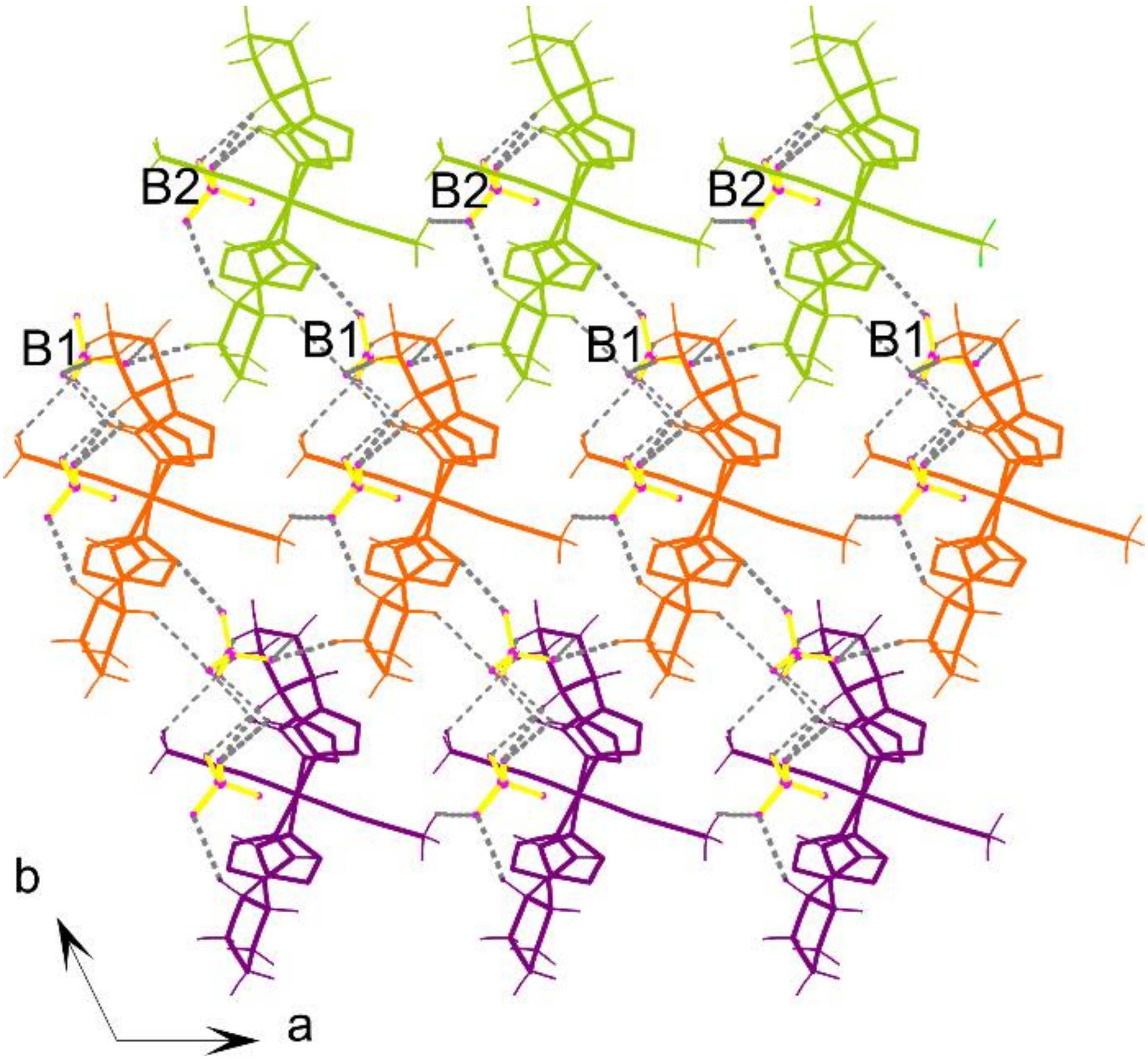


**Figure SI 28.** Forming of a 3D network of weak contacts (shown with grey dashed line) between supramolecular layers through interactions with $BF_4^-$ anions at 80 K in **1B·solv**. Polycationic chains belonging to separate layers are shown with common color (lime, orange, violet). Fluorine and boron atoms are shown with pink color; *C-F* bonds are shown with yellow color. $B(1)F_4^-$ anions interact with both layers, $B(2)F_4^-$ – with one layer. Direct weak contacts between chains are omitted for clarity.

**1B·solv** is distinguished from the other complexes by the presence of non-coordinated acetonitrile molecules. Guest acetonitrile molecules are engaged in forming weak contacts both with hydrogen atoms of supramolecular layers and fluorine atoms of anions (**Fig. SI 29**). It should be noted that non-coordinated solvent molecules manifest themselves as donors of electron density (through hydrogen atoms of methyl groups) in a relation to the fluorine atoms of the tetrafluoroborate anions and as acceptors in a relation to the atoms of polycationic chains (**Table SI 5**). One of the solvent molecules is involved in forming weak contacts only with one polymeric chain (through *N87* atom), while the other one forms weak contacts (through *N97* atom) with neighboring supramolecular layers, thus participating in interactions between layers at 80 K (corresponding the LS state of the Fe(II) cations). Both solvent molecules form contacts with both counterions.

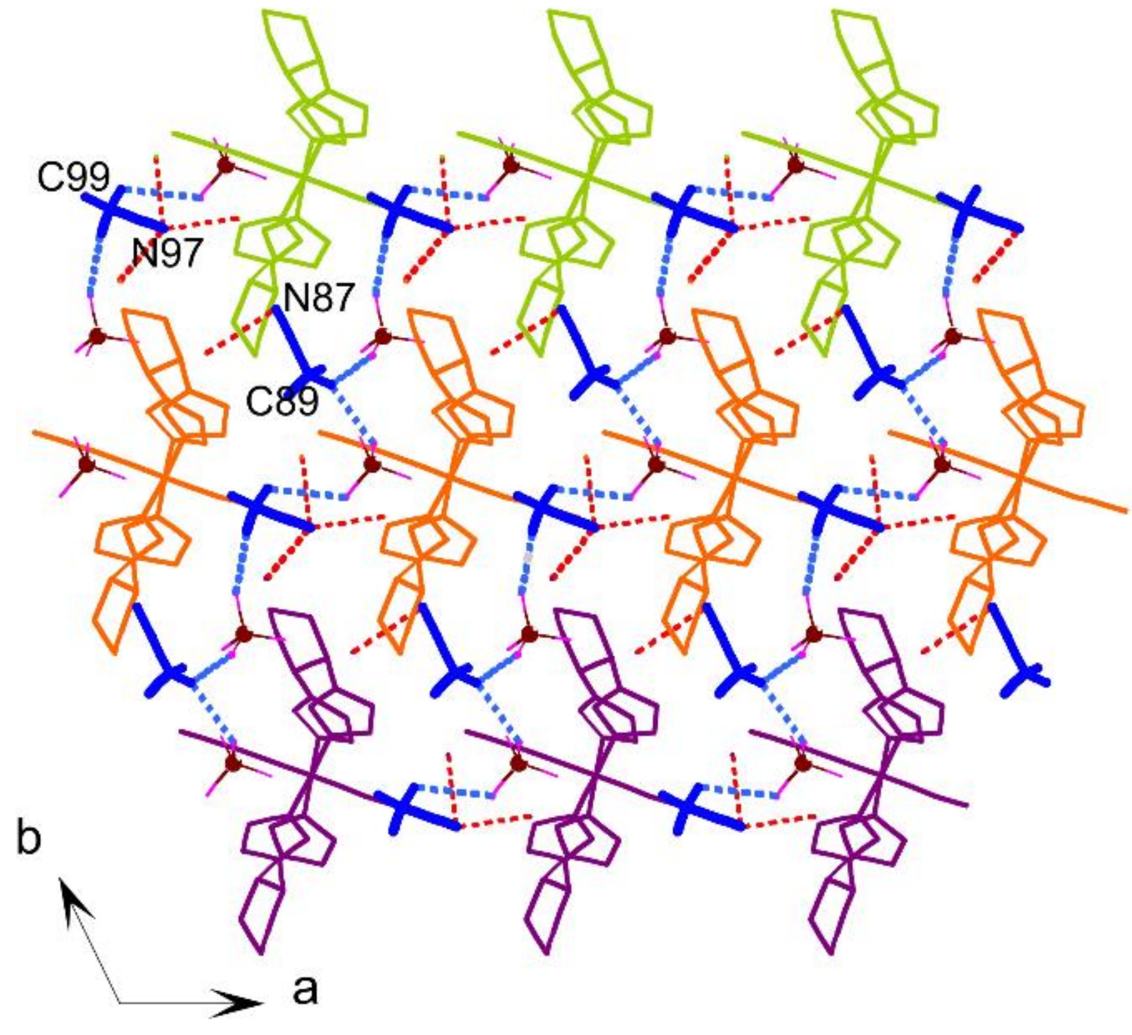


**Figure SI 29.** Weak interactions of non-coordinated acetonitrile molecules (represented with blue sticks) with polycationic chains and tetrafluoroborate anions at 80 K in **1B·solv**. Boron atoms are shown with dark red, fluorine atoms – with pink color. Polycationic chains belonging to separate layers are shown with common color (lime, orange, violet). $N_{solvent}\cdots H\text{-}C_{chain}$ contacts are shown with red color, $C\text{-}H_{solvent}\cdots F_{\text{anion}}$ – with light blue line. Direct weak contacts between chains and between chains and anions are omitted for clarity. *C-H* bonds in polymeric chains are not shown for clarity.

Thermally induced LS → HS transition on **1B·solv** and **2B** causes a decrease in a number of the weak interactions in the crystal structures (**Tables SI 5-6**). In **1B·solv** at 250 K (corresponding to the HS state of the compound) each of non-coordinated nitrile molecules interacts only with one polycation chain (namely through *C1-H1⋯N87* and *C55-H55⋯N97* contacts, respectively) and one $BF_4^-$ anion (through *C89-H89A⋯F23* and *C99-H99B⋯F12* contacts, respectively). Also one of the two weak contacts participating in the forming of supramolecular layers disappears at the HS state and the layers are formed *via* single *C71-H71B⋯N43* contact (**Table SI 5**). A similar change is observed in the structure of **2B**: at 310 K (corresponding the HS state) contact *C70-H70C⋯N22* (which is involved in the formation of double supramolecular layers) disappears (**Table SI 6**). The changes observed in the **1A** and **2A** (being SCO-inactive) due to the rise of temperature to 250 K are less significant. A little decrease in a number of contacts between anions and supramolecular layers (**Tables SI 3-4**) is observed due to the thermal expansion of the crystals of **1A** and **2A**, however, the abovementioned pattern of the weak contacts is preserved.

In order to sum up weak contacts analysis in the crystal structures of the complexes and to describe structural patterns more quantitatively we calculated distances between Fe(II) centers within polymeric chain and distances between unit vectors defined by bridged metal cations within interacting chains. Also, we defined planes through Fe(II) cations (that belong to the polymeric units, which are involved in the formation of the abovementioned supramolecular layers) and calculated distances between them, too. The results of these calculations are presented in **Table SI 8**.

**Table SI 8.**

| **Compound** | **Homochiral** | | | | | **Heterochiral** | | | |
|---|---|---|---|---|---|---|---|---|---|
| | **1B·solv** | | **2B** | | **1B** | **1A** | | **2A** | |
| **T / K** | **80** | **250** | **80** | **310** | **80** | **80** | **250** | **80** | **250** |
| **Spin state** | **LS** | **HS** | **LS** | **HS** | **LS** | **HS** | | **HS** | |
| Intra chain | 9.147(3) | 9.747(3) | 8.763(4) | 9.027(3) | 8.74(1) | 9.334(2) | 9.427(3) | 9.492(3) | 9.529(4) |
| Inter chain | 9.370(4) | 9.673(3) | 10.085(4) | 10.476(3) | 10.06(1) | 8.377(2) | 8.498(3) | 8.438(3) | 8.530(4) |
| Inter layer | 9.830(4) | 10.019(4) | 9.560(4) | 9.839(3) | 9.33(1) | 9.474(2) | 9.489(3) | 9.475(3) | 9.654(4) |
| Inter chain within double layer | N.A. | | 7.696(4) | 7.646(3) | 8.45(1) | N.A. | | | |
| Interactions between chains | indirect | | direct | | direct | direct | | indirect | |

The distances between bridged Fe(II) centers in all four compounds are quite close: at 80 K they are equal to 9.147(3), 8.763(4), 9.334(2), 9.492(3) and 8.74(1) Å in **1B·solv**, **2B**, **1A**, **2A** and **1B**, respectively. The temperature-induced HS $\rightarrow$ LS transition leads to the increase in these distances in **1B·solv** and **2B**. At HS state the corresponding distances are equal to 9.747(3) in **1B·solv** (at 250 K) and 9.027(3) Å in **2B** (at 310 K). The rise of temperature causes only a slight increase in these distances in **1A** and **2A** due to the lack of SCO in these compounds. At 250 K the corresponding distances are equal to 9.427(3) in **1A** and 9.529(4) Å in **2A**. The SCO phenomenon induces also greater rise of the inter-chain and inter-layer distances in SCO-active **1B·solv** and **2B** compared to the **1A** and **2A**. However, it should be mentioned that SCO almost has no effect on the distance between layers within "double layer", which is defined only for **2B** due to the structural features described above. Also, this fact additionally justifies our choice of the definition of this "double layer" because it is a quite obvious equivalent of the "mono" layers observed in other three compounds.

Comparing the distances between interacting chains and supramolecular layers we concluded that in heterochiral **1A** and **2A** they are shorter than the corresponding distances in SCO-active **1B·solv** and **2B**. It can be explained by the differences in anion distribution around polymeric units as we have discussed in the main text. We assume that the pseudohexagonal anion distribution observed in heterochiral **1A** and **2A** (**Fig. 5**, **left**) leads to the more effective inter chain interactions that manifests as shorter distances between them. While quite irregular anion distribution taking place in homochiral **1B·solv** and **2B** (**Fig. 5**, **right**) can decrease effectiveness in interactions between chains. Also, it has to be remembered that only half of the anions (one of the two crystallographically independent counterions) in homorochiral **1B·solv** and **2B** is involved in interactions between supramolecular layers (**Fig. SI 25**, **Fig. SI 28**), while in heterochiral **1A** and **2A** all anions are involved in these interactions. This fact is in favor of the pronounced more effective inter-chain interactions in heterochiral compounds.

**Comparison of $\chi_M T(T)$ dependences for initial 1B·solv and after wetting desolvated sample 1B with acetonitrile**

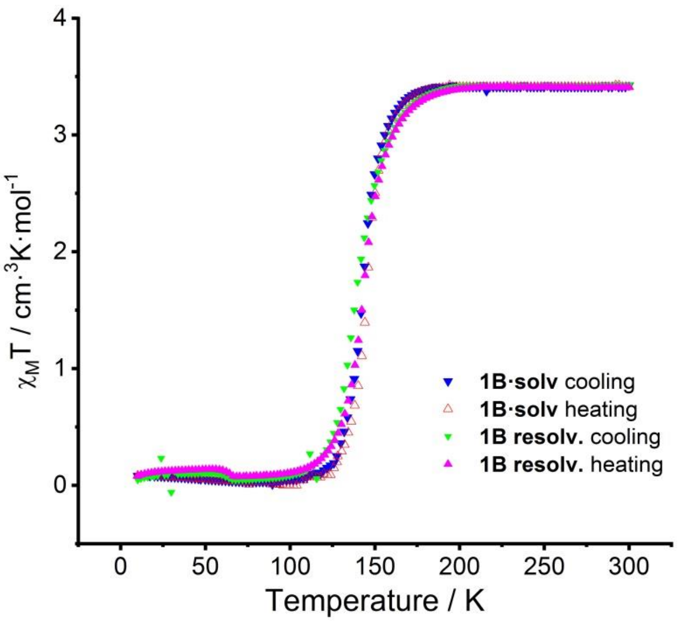


**Figure SI 30.** Comparison of $\chi_M T(T)$ dependences for initial **1B·solv** and after wetting desolvated sample **1B** with acetonitrile (resolvated).
